# Five genomic regions have a major impact on fat composition in Iberian pigs

**DOI:** 10.1038/s41598-019-38622-7

**Published:** 2019-02-14

**Authors:** R. N. Pena, J. L. Noguera, M. J. García-Santana, E. González, J. F. Tejeda, R. Ros-Freixedes, N. Ibáñez-Escriche

**Affiliations:** 1 Departament de Ciència Animal, Universitat de Lleida-Agrotecnio Center, 25198 Lleida, Spain; 2 IRTA, Genètica i Millora Animal, 25198 Lleida, Spain; 3 INGA FOOD S.A, 06200 Almendralejo, Spain; 4 Tecnología de los alimentos, Universidad de Extremadura, 06006 Badajoz, Spain; 5 The Roslin Institute, Edinburgh University, Easter Bush, EH25 9RG UK; 6 Institute for Animal Science and Technology, Universitat Politècnica de València, 46022 Valencia, Spain

## Abstract

The adipogenic nature of the Iberian pig defines many quality attributes of its fresh meat and dry-cured products. The distinct varieties of Iberian pig exhibit great variability in the genetic parameters for fat deposition and composition in muscle. The aim of this work is to identify common and distinct genomic regions related to fatty acid composition in Retinto, Torbiscal, and Entrepelado Iberian varieties and their reciprocal crosses through a diallelic experiment. In this study, we performed GWAS using a high density SNP array on 382 pigs with the multimarker regression Bayes B method implemented in GenSel. A number of genomic regions showed strong associations with the percentage of saturated and unsaturated fatty acid in intramuscular fat. In particular, five regions with Bayes Factor >100 (SSC2 and SSC7) or >50 (SSC2 and SSC12) explained an important fraction of the genetic variance for miristic, palmitoleic, monounsaturated (>14%), oleic (>10%) and polyunsaturated (>5%) fatty acids. Six genes (*RXRB*, *PSMB8*, *CHGA*, *ACACA*, *PLIN4*, *PLIN5*) located in these regions have been investigated in relation to intramuscular composition variability in Iberian pigs, with two SNPs at the *RXRB* gene giving the most consistent results on oleic and monounsaturated fatty acid content.

## Introduction

The Iberian pig is a traditional breed native to the west and southwest of the Iberian Peninsula characterised by precocious development of fat depots and high deposition of intramuscular fat (IMF; ca. 8–12%)^1^, and small litter size^2^. The breed is mainly used for the production of high-quality dry-cured products, intended for niche, traditional or premium markets. In Iberian pigs, IMF is particularly rich in oleic acid (C18:1, n-9), a monounsaturated fatty acid that gives fat an oily aspect very appreciated by consumers. Pigs fed an acorn-based diet have around 55% of oleic acid content and relative low concentrations of linoleic and palmitic acids (around 8% and 20%, respectively)^3^. The breed is structured in 5 varieties (Ministry of Agriculture, Fishing and Food, Spain) which exhibit distinct growth, fattening and reproductive abilities. Pigs of the Torbiscal variety are characterised by a faster growing rate resulting in heavier carcasses and better noble cut yields than Entrepelado and Retinto^4^. On the other hand, the latter are more prolific than Torbical sows, their meat is darker and, although fattier, contains less saturated fat. These production differences indicate the segregation of variety-specific genetic variants, which, once known, could be combined to improve the productivity of the breed. Unlike most modern European breeds, the Iberian pigs do not show signs of admixture with Chinese breeds^5^, which emphasises the need to develop breeding strategies specific for the Iberian varieties. In a previous study^6^ we confirmed the phenotypic differences between these three varieties. We also showed that fat deposition and the fatty acid profile of intramuscular (IMF) muscle and subcutaneous fat in Iberian pigs are traits of moderate or high heritability, indicating that selective breeding can be profitably used for the enhancement of such characteristics. The aim of this study is to use a high-density SNP genotyping platform to identify variety-specific variants associated with IMF content and composition through the analysis of a complete diallelic cross of three Iberian varieties.

## Results

### Phenotype differences between diallelic crosses

A complete diallelic cross was set up by crossing pigs of three Iberian varieties, resulting in the generation of 9 different genotypes (Table 1). In this population we have previously shown that there is a genetic variety effect on the content of several IMF fatty acids^6^. In particular, the Torbiscal variety has more saturated fatty acids (SFA) and less monounsaturated fatty acids (MUFA) and MUFA/SFA ratio than Retinto pigs, while Entrepelado exhibits an intermediate phenotype (ref.^6^ and Supplementary Table S1). Depending on the trait, the most relevant role is played by additive, heterosis and maternal effects or a combination of them. Here we have performed a genome-wide association analysis in an effort to identify the sources of these differences, focussing on IMF content and concentration of main saturated (SFA), monounsaturated (MUFA), and polyunsaturated (PUFA) fatty acids in *longissimus thoracis* muscle. After filtering the genotyping data, only 20,580 SNPs (less than one third) were retained for further analysis. A large fraction of the discarded SNPs did not segregate in Iberian pigs, which emphasises the need to develop breed-specific markers.

**Table 1:** Distribution of the pigs used in this study according to the parental and maternal Iberian variety used in each cross.

Male Variety. Boars used^a^	Female Variety. Sows used^a^		
	TT (81)	EE (24)	RR (39)
TT (18)	TT (77)	TE (21)	TR (26)
EE (12)	ET (17)	EE (21)	ER (13)
RR (14)	RT (106)	RE (13)	RR (48)

Only the 342 offspring finally used in the GWAS analysis are indicated.

^a^Varieties: TT = Torbiscal; EE = Entrepelado; RR = Retinto; RT = Retinto × Entrepelado; TR = Torbiscal × Retinto; RE = Retinto × Entrepelado, ER = Entrepelado × Retinto, TE = Torbiscal × Entrepelado; ET = Entrepelado × Torbiscal.

### Population structure

**Figure 1:**
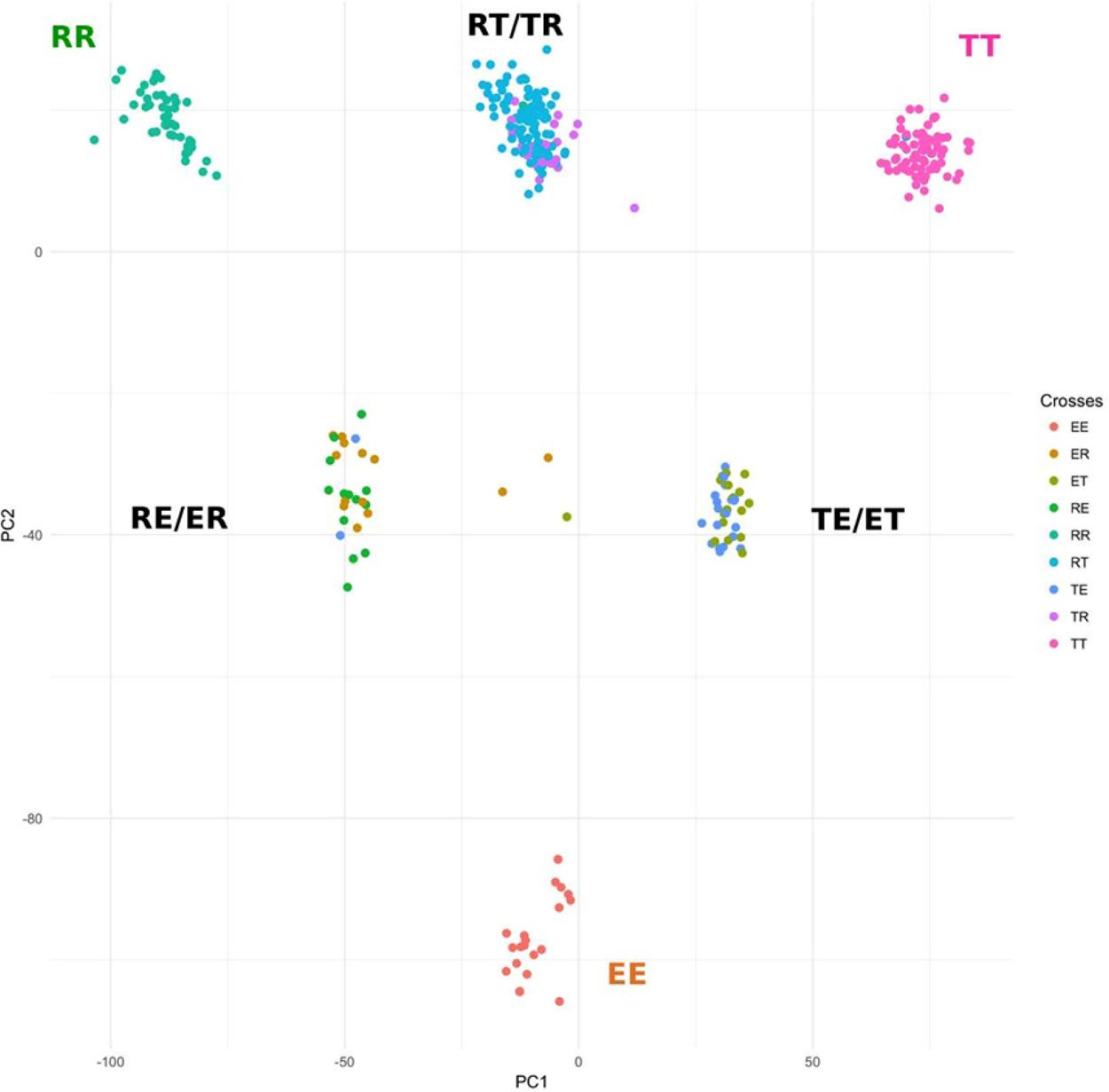
Population structure identified by principal component (PC) analysis. The three Iberian varieties (EE – Entrepelado; RR – Retinto; TT – Torbiscal) can be separated by the analysis, while the crossed pigs occupy intermediate positions.

Population stratification was assessed by a principal component analysis using the genotypic information of the 20,580 SNPs. The genetic diversity of the three pure varieties could be separated based on the PC results while the reciprocal crosses occupied intermediate positions between pure varieties following an expected pattern (Fig. 1). This information was used in the GWAS analysis to account for population stratification and control false positive results representing genetic differences from ancestry rather than genes associated with a trait^7^.

### GWAS results

We observed only one relevant association with IMF content at genomic level, located at pig chromosome (SSC) 14, 137.5 Mb, with a strong Bayer Factor (BF) of 16.52. This association explained a very minor fraction of the genetic variance of the trait (1.42%; Table 2). In addition, some suggestive regions were detected in SSC3 (44 Mb) and 9 (45 Mb) in relation to IMF content (Supplementary Fig. S1A). In contrast, 76 strong trait-SNP associations (Bayes Factor (BF) > 10) were found with at least one of the fat compositional traits. These involved 65 distinct SNPs that were associated with at least one of the fat compositional traits. In total, 32 distinct not overlapped regions (windows) distributed across 14 chromosomes were identified with BF > 10 (Supplementary Table S2), 15 of which explained at least 1% of the genetic variance for at least one trait (Table 2). Among them, there were two very strong association (BF > 100) involving four SNPs in SSC2 and SSC7. Additionally, two strong associations (BF > 50) were detected in SSC2 related to palmitoleic and PUFA content, plus nine markers associated with miristic acid content in SSC12. These five regions with the highest BF values also explained a large fraction of the genetic variance for miristic acid (SSC12), palmitoleic acid (SSC2, two regions), PUFA content (SSC2) and oleic acid and total MUFA content (SSC7), as detailed below.

**Table 2:** Genomic regions with strong associations with fatty acid content and compositional traits in *longissimus thoracis* muscle (Bayes factor (BF) > 10) and accounting for >1% genetic variance (%GV) in at least one trait.

SSC^a^	Region^b^	n. SNP	Marker with highest BF	Trait^c^	BF	%GV	Candidate genes^d^
1	173.2–173.8	4	ALGA0007029	C14:0	11.53	4.13	
2	20.0–20.9	1	ALGA0109169	PUFA	81.97	4.95	*HSD17B12*
	75.0–75.6	2	MARC0048160	C16:1	624.06	14.44	*PLIN3*, ***PLIN5***, ***PLIN4***, *PIAS4*, *PIP5K1C*, *S1PR4*, *GNA15*, *GADD45B*, *CSNK1G2*, *ATP8B3*, *NDUFS7*, *STK11*, *ABCA7*
	90.4–97.9	4	DRGA0003183	C16:1	28.36	2.21	*SERINC5*, *ACOT12*, *MEF2C*
				C18:1	10.97	1.34	
				MUFA	13.45	2.25	
	100.1–106	4	ALGA0014789	MUFA/SFA	24.32	3.30	*NR2F1*, *ARSK*
				C16:1	52.33	4.07	
				C18:1	10.7	1.08	
				MUFA	12.63	1.33	
	115.1–115.7	3	MARC0058544	C14:0	16.54	1.35	*STARD4*
4	123.0–127.9	2	MARC0014258	MUFA/SFA	31.00	1.94	*AGL14*, *ABCD3*, *ABCA4*, *GFI1*, *SLC44A3*
6	3.0–4.0	2	ALGA0103513	C16:1	23.91	1	*FOXC2*, *COX4I1*, *MBTPS1*
7	25.0–25.9	2	H3GA0020505	C18:1	20.12	2.10	*APOM*, *ABHD16A*, *CYP21A2*, *SLC44A4*, *PPT2*, ***PSMB8***, *HSD17B8*, ***RXRB***, *HMGCLL1*
	103.3–105.6	3	MARC0033066	C16:1	11.80	1.81	*TSHR*, *SEL1L*
	113.2–115.2	2	ASGA0036470	C18:1	117.66	10.40	*TTC7B*, *LGMN*, ***CHGA***, *COX8C*, *SERPINA12*, *SERPINA6*
				MUFA	174.95	15.48	
				MUFA/SFA	117.36	6.13	
8	13.7–17.4	2	DRGA0008365	PUFA	37.36	3.26	*GBA3*, *PPARGC1A*
12	36.9–38.9	9	ASGA0054436	C14:0	77.66	17.13	*MED13*, *PIGW*, ***ACACA***
14	137.5	1	DRGA001476	IMF	16.52	1.42	*EBF3*

^a^SSC – *Sus scrofa* chromosome. ^b^Region (in Mb) refer to pig genome assembly 11.1 coordinates. ^c^Traits: Fatty acids, in % of total fatty acids; SFA – saturated fatty acid content; MUFA – monounsaturated fatty acid content; MUFA – monounsaturated fatty acid content; MUFA/SFA – MUFA to SFA ratio. ^d^Positional candidate genes in bold have been investigated in this study. Gene full names in Supplementary Table S3.

In chromosome SSC2 we detected seven genomic regions strongly associated with fatty acid composition in the *longissimus thoracis* muscle (Supplementary Table S2). Three positional candidate genes, perilipin 3 (*PLIN3*), 4 (*PLIN4*) and 5 (*PLIN5*), map to the 75 Mb region, which showed the strongest association with palmitoleic acid content (BF = 624.1) and explained 14.44% of the genetic variance for this trait (Table 2). Two other regions in SSC2, at 90.4–97.9 Mb and 100.1–106 Mb (comprising 4 SNPs each), showed strong association with palmitoleic, oleic and MUFA content. The latter, in addition, was also associated to MUFA/SFA ratio. Given the relative close distance between these two regions, it cannot be ruled out that they might represent the same causal effect. Important genes in this region are *ACOT12* (89.6 Mb), a cytosolic thioesterase involved in the hydrolysis of medium- to long-chain fatty acyl-CoAs (C12–C18) to their free fatty acids, and *NR2F1* (100.4 Mb), a sterol-binding transcription factor (also known as COUP-TF1) involved in regulating the synthesis and transport of triglycerides in enterocytes^8^. Also of relevance in this chromosome is the association of the 20.0–20.9 Mb region with total PUFA content (BF = 81.97), which explained 4.95% of the genetic variance for this trait. A promising candidate gene in this region is *HSD17B12*. This gene encodes a 17 beta-hydroxysteroid dehydrogenase involved in estradiol synthesis in the ovary but also in fatty acid elongation in other tissues. The enzyme catalyses the second of the four reactions of the long-chain fatty acids elongation cycle towards very long-chain fatty acids, which are substrate of polyunsaturation events for instance by the fatty acid desaturases FADS2 and FADS3.

Two regions in SSC7, at positions 25.0–25.9 and 113.2–115.2 Mb, also displayed strong associations with more than one compositional trait (oleic acid and MUFA content and the MUFA/SFA ratio; Supplementary Table S2). Two SNPs at 113.2–115.2 Mb explained 10.40% and 15.48% of the genetic variance of oleic acid and MUFA content in intramuscular fat, respectively. Two interesting candidate genes are located at the 25 Mb region. The retinol-X-receptor beta (*RXRB*) gene is a strong functional candidate gene as it drives the lipogenic activities of retinoic acid, the bioactive vitamin A^9^. Another interesting gene is *PSMB8* which encodes a protease required for the differentiation of preadipocytes into adipocytes^10^ and which disruption causes lipodystrophy^11^. The catestatin hormone, a product of the chromogranin A (*CHGA*) gene, which maps to SSC7, 114.3 Mb, is involved in the mobilisation of fatty acids from the adipose tissue. This gene encodes also pancreastatin, a hormone that regulates the insulin:glucagon ratio released from pancreas^12^. Therefore, *CHGA* represents a potential physiological candidate gene in this MUFA-associated region.

Finally, among the results it is also noteworthy a group of 9 close markers in the 36.9–38.9 Mb region of SSC12, all of them associated with miristic acid content. This association accounted for 17.13% of the genetic variance for miristic acid content, which represents the greatest effect detected in this study for a trait. This region co-localises with the acetyl-coA carboxylase (*ACACA*) gene (38.5–38.9 Mb), a gene that has been studied before in relation to muscle fatty acid composition in Iberian and Duroc pigs^13–15^.

### Functional evaluation of GWAS regions co-localising genes

Genes mapping to 2-Mb windows around the 65 markers displaying strong associations (BF > 10) with one or more fatty acid compositional traits were catalogued using gene ontology terms and pathway participation in order to identify candidate genes whose function would be related (directly or indirectly) to fatty acid transport or metabolism. From the initial 974 genes, we identified 112 genes that participated in fatty acid biosynthesis, transport or catabolism, or that played a role in the differentiation and maturation of adipocytes (Supplementary Table S3). These constitute positional and functional candidate genes for the regions with BF > 10. Genes co-localising with regions explaining >1% of the genetic variance of at least one trait are indicated in Table 2. A subset of those genes were selected for further analysis considering if their molecular function was related to the trait affected in each region

### Characterisation of potential candidate genes

We intended to sequence the proximal promoter and full cDNA of a group of 6 selected candidate genes in 4 Entrepelado, 4 Retinto and 4 Torbiscal pigs in order to identify polymorphisms segregating in these Iberian varieties. This list included *PLIN4* and *PLIN5* as candidate genes for the SSC2 75.0–75.6 Mb region explaining 14.44% of the genetic variance for palmitoleic acid content; *PSMB8* and *RXRB*, for the SSC7 25–25.9 Mb region for oleic acid content; *CHGA* for the SSC7 113.2–115.2 Mb region that explained more than 10% of the genetic variance for oleic and total MUFA content; and *ACACA* for the SSC12 36.9–38.9 Mb region for miristic acid content. The sequencing targets included exonic sequence and regulatory regions (promoters or putative enhancers). Unfortunately, we were not able to amplify the promoter or the exonic sequences of the *PLIN4* gene, which was hampered by the repetitive nature of this gene sequence. From the rest of the genes, a subset of the polymorphisms identified by Sanger sequencing on *PLIN5*, *RXRB*, *PSMB8*, *CHGA* and *ACACA* were selected to be genotyped by high resolution melting (HRM) PCR in 208 purebred pigs (28 Entrepelado, 72 Retinto and 108 Torbiscal) which included all purebred pigs from the GWAS experiment (n = 160) plus 48 additional pigs.

The most relevant result was with the *RXRB* polymorphisms. The two promoter polymorphisms (rs327226101 and rs331605500) were completely linked and in ample linkage disequilibrium (LD) with the distal SNP on exon 9 (LD = 0.94). The SNP on exon 9 (rs80789331) was associated with IMF content, with AA pigs having lower IMF levels than both CA and CC pigs (Table 3). This was an unexpected result as no signal for IMF was detected in this chromosome in the GWAS. The effect was particularly evident in the Torbiscal pigs (Table 3) where CC and AA pigs differed in 2.15% IMF content. This effect could not be captured in the other two varieties, which could be due to the lower number of pigs or it could indicate a variety-specific effect, due to a linked mutation in Torbiscal. However, the most significant associations for this gene were with compositional traits such as palmitic and oleic fatty acids, and also SFA, MUFA and PUFA content (Tables 4 and S4). The alleles that increased oleic acid content, MUFA and PUFA had a negative effect on SFA content. When the effects were studied in the individual Iberian varieties, the same effects were reproduced in Retinto animals but not in the others, suggesting a linked causal mutation specific of this variety (Tables 4 and S5). The magnitude of the effects was relevant between alternative homozygotes. For instance, for the *RXRB* rs327226101 promoter SNP, there was a 2% difference in oleic acid content between Retinto GG and TT pigs (Table 4) while differences in SFA and MUFA content were close to 4% and 3%, respectively.

**Table 3:** Effect of the *RXRB* exon 9 SNP rs80789331 on the intramuscular fat (IMF) content in *longissimus thoracis* muscle of Iberian pigs.

Animals	RXRB rs80789331	Difference in			
	genotypes compared	IMF content, %	SE	95% CI	p-value
Whole purebred population	CC-AA	1.61	0.53	0.36, 2.86	**0.008**
	CA-AA	1.01	0.43	0.00, 2.02	**0.050**
Torbiscal variety	CC-AA	2.15	0.74	0.39, 3.91	**0.012**
	CA-AA	1.23	0.56	−0.11, 2.58	0.079

SE – standard error; CI – confidence interval.

**Table 4:** Effect of the *RXRB* promoter SNP rs327226101 on the intramuscular fat composition in *longissimus thoracis* muscle of Iberian pigs analysed together or by variety.

Animals	Trait^a^	Genotypes compared	Difference in trait content	SE	95% CI	p-value
Whole purebred population	C16:0	TT-GG	0.80	0.27	0.15, 1.44	**0**.**011**
		TT-GT	0.56	0.20	0.08, 1.04	**0**.**018**
	C18:0	TT-GG	0.67	0.28	0.01, 1.32	**0**.**045**
	C18:1	TT-GG	−0.85	0.40	−1.78, 0.09	0.086
		TT-GT	−0.84	0.29	−1.54, −0.14	**0**.**013**
	SFA	TT-GG	1.47	0.49	0.32, 2.63	**0**.**008**
		TT-GT	0.97	0.36	0.11, 1.83	**0**.**023**
	MUFA	TT-GG	−1.12	0.47	−2.22, −0.02	**0**.**046**
		TT-GT	−0.94	0.35	−1.77, −0.12	**0**.**020**
	PUFA	TT-GG	−0.37	0.15	−0.72, −0.03	**0**.**028**
		GT-GG	−0.35	0.13	−0.67, −0.03	**0**.**026**
	MUFA/SFA	TT-GG	−0.09	0.03	−0.16, −0.02	**0**.**013**
		TT-GT	−0.06	0.02	−0.12, −0.01	**0**.**014**
Retinto variety	C16:0	TT-GG	1.99	0.53	0.71, 3.28	**0**.**001**
		TT-GT	1.51	0.43	0.48, 2.54	**0**.**002**
	C16:1	TT-GG	−0.80	0.31	−1.53, −0.06	**0**.**032**
	C18:0	TT-GG	1.93	0.55	0.62, 3.25	**0**.**002**
		TT-GT	0.95	0.44	−0.11, 2.00	0.086
	C18:1	TT-GG	−2.28	0.78	−4.14, −0.41	**0**.**013**
		TT-GT	−1.98	0.62	−3.47, −0.48	**0**.**007**
	SFA	TT-GG	3.90	1.02	1.44, 6.36	**0**.**001**
		TT-GT	2.52	0.82	0.55, 4.49	**0**.**009**
	MUFA	TT-GG	−3.18	0.98	−5.53, −0.83	**0**.**005**
		TT-GT	−2.40	0.78	−4.28, −0.53	**0**.**009**
	PUFA	TT-GG	−0.75	0.21	−1.25, −0.25	**0**.**002**
		GT-GG	−0.62	0.19	−1.09, −0.16	**0**.**006**
	MUFA/SFA	TT-GG	−0.23	0.07	−0.39, −0.07	**0**.**002**
		TT-GT	−0.16	0.05	−0.29, −0.04	**0**.**008**

^a^All traits are expressed as % of total fatty acid content. SFA - saturated fatty acids; MUFA - monounstaturated fatty acids; PUFA - polyunsaturated fatty acids; SE - Standard error.

Results in other mutations were less consistent. For instance, the *PSMB8* rs80801731 promoter mutation was significantly associated with oleic acid content (Supplementary Table S4), a trait that was selected for using *PSMB8* as a candidate gene. However, this association was not detected in any of the three varieties when analysed separately, which might indicate that this is a spurious result. Of the two mutations of the *CHGA* gene, only the rs322866746 showed significant differences between AG and GG pigs on stearic acid, oleic acid, SFA and MUFA content (Supplementary Tables S4 and S5) when analysed in the whole purebred population and also in the Entrepelado variety. The magnitude of the effects in Entrepelado were lower than those described above for the *RXRB* SNPs. On the other hand, the effects of the *CHGA* rs318914549 SNP on IMF composition (oleic acid, SFA and MUFA) were only detected in Retinto animals, with CC and CT pigs having ~2.5% and ~3% more oleic acid and MUFA than TT pigs, respectively. The opposite effect was seen in SFA content, probably as an indirect effect on the percentage content. Finally, three SNPs in *ACACA* were selected as candidate genes for miristic acid content. However, none of them was associated with this trait. Instead, the 12:38727103 G > A mutation in a putative *ACACA* enhancer on intron 24 was associated with palmitic, oleic, SFA and MUFA content both in the whole population and in the Retinto variety (Supplementary Tables S4 and S5). In addition, no significant associations were found between a missense mutation in the exon 3 of the *PLIN5* gene (Supplementary Table S6) and any of the compositional traits.

## Discussion

We have performed a GWAS of three distinct Iberian pig varieties and their reciprocal crosses to identify relevant genomic regions associated with IMF content and composition in these particular varieties. This is the first GWAS performed in pure variety Iberian animals, which makes results very valuable for breeders working with this breed. Despite losing nearly 2/3 of the potential genotypes in the high-density panel due to lack of segregation of these SNPs, we were able to detect 32 genomic regions with strong associations with several fatty acid compositional traits. Out of these, two regions stand out by their ability to capture a very significant fraction of the genetic variance of monounsaturated fatty acids. These are the 75–75.6 Mb region in SSC2, which explains 14.4% of the genetic variance for palmitoleic acid content, the 113.2–115.2 Mb region in SSC7 (10% and 15% of the genetic variance of oleic acid and MUFA content, respectively). Two additional regions in SSC2 captured about 4–5% of the genetic variance for PUFA (20.0–20.9 Mb) and palmitoleic acid (100.1–106 Mb), while the SSC12 36.9–38.9 Mb region explained 17% of the genetic variance of miristic acid content.

Oleic and palmitoleic acid are the two main MUFA in pork. In Iberian meat, usual percentages are in the range of 45–55% and 4–5%, respectively. Monounsaturated fat represents a healthier alternative to the consumption of SFA, without the rancidity odour risk of PUFA^16^. In Iberian cured products the content of oleic acid contributes to the taste and visual aspect sought after by consumers which are willing to pay more for a high-quality product. This puts into context the relevance of identifying genomic regions which explain a large fraction of the genetic variance for the main MUFA in pork.

We have studied 6 genes located in some of these regions. All of them have a molecular function related to fatty acid metabolism (*RXRB*, *ACACA*), storage (*PLIN4*, *PLIN5*, *CHGA*) or adipogenesis (*PSMB8*). After sequencing coding and regulatory regions, a subset of SNP markers was selected to be genotyped in a group of 208 purebred Iberians from the Entrepelado, Retinto and Torbiscal varieties. The most consistent result was observed for three partially linked SNPs in the *RXRB* gene (two in the promoter and one at the end of the gene, on exon 9). Considering the rs80789331 SNP on exon 9, pigs of the CC genotype had more SFA and less MUFA and oleic acid than either AA or CA pigs. RXRB is an ubiquitously expressed transcription factor from the nuclear receptor superfamily also known as NR2B2. By binding 9-cis-retinoid acid, it can integrate a number of metabolic signalling pathways related to energy metabolism by forming homodimers or, more frequently, heterodimers with other nuclear receptor members such as RARα,β,γ, RXRα,γ or PPARα,β,δ/γ^17^. In liver, activation of RXRB increases the expression of stearoyl-coA destaturase (*SCD*) and *CD36* fatty acid translocase^18^ and *in vitro* adipocyte differentiation^17^. CD36 is a membrane transporter very abundant in skeletal muscle that participates in the uptake of long- and very long-chain fatty acids. The SCD enzyme, on the other hand, catalyses the limiting step in the synthesis of monounsaturated fatty acids (oleic and palmitoleic acids) from saturated substrates (stearic and palmitic acids). A mutation in the *SCD* proximal promoter has been associated to higher oleic acid content in Duroc pork without affecting the total fat content in muscle or backfat^19^. However, this mutation does not segregate in Iberian pigs (Estany *et al*.^19^ and own data on our Iberian resource population). It is likely that the effect of *RXRB* polymorphisms on MUFA content of the Iberian pigs is driven by differential expression on the *SCD* gene and/or other genes related to fatty acid elongation, desaturation or oxidation. However, one must be cautious with assigning functional relevance only based on the position of a mutation. The most proximal rs327226101 mutation lays −35bp from the transcriptional start site of *RXRB*, and could therefore affect change the functionality of the core promoter of this gene. However, on the other strand this mutation lays in first exon/intron of the two transcriptional variants of the *SLC39A7*, which encodes for a Zn^2+^ Golgi transporter. A simultaneous effect of the rs327226101 SNP on either or both genes cannot be ruled out. Future analysis of muscle transcriptomics will provide complementary information to the GWAS results.

The conjoint effect of the *RXRB* SNPs on SFA, MUFA and oleic acid content was also observed for the rs322866746 and rs318914549 mutations on exon 6 of the *CHGA* gene and for an intron 5 mutation in *ACACA*, and it is due to the high genetic correlation between these traits (positive between MUFA and oleic acid and negative between SFA and MUFA or oleic)^20^. Chromogranin A is a secretory proprotein expressed from the *CHGA* gene that is cleaved in several active peptidic hormones including pancreastatin, vasostatin, and catestatin^12^. The latter is a pleiotropic hormone that has effects on promoting angiogenesis, lowering blood pressure, immunomodulation, and also has a negative effect on fat deposition by increasing fatty acid oxidation^12^. Although this is a very interesting gene, the two missense mutations we found overlapped with none of the biologically active peptides, ruling out the causality of these mutations on the IMF composition. Also, a promising mutation in exon 3 of the *PLIN5* gene which promoted a non-conservative change at the protein sequence (Supplementary Table S6), was not associated with intramuscular fatty acid composition. Still, the relevance of this gene regarding monounsaturated fatty acid content has been highlighted recently, in relation to the activity of the hormone-sensitive lipase LIPE^21^. Therefore, this is a gene to be pursued in future experiments.

The acetyl-coA carboxylase alpha (*ACACA*) gene was selected as a physiological and positional candidate gene for the region on SSC12 (38 cM) for miristic acid content. The ACACA enzyme is a limiting factor in the synthesis of long chain fatty acids and plays an important role in the conversion of acetyl-CoA to malonyl-CoA, acting as a precursor of miristic and palmitate acids^13^. In addition, its activity slows the importation of fatty acids to the mitochondria for β-oxidation. Malonyl-CoA, as a product of the enzyme ACACA, is the intermediate substrate of other enzymes that will synthesize SFA and MUFA^15^. In pigs, *ACACA* is a 287 Kb-long gene structured in 56 exons, which is transcribed into five variants that differ in the use of certain exons and with a complex regulation from several promoters^15^. Several polymorphisms have been described in both coding and regulatory regions of this gene, some of which have been previously studied in Iberian pigs or in experimental crosses with Iberian pigs^15^. A synonymous polymorphism in exon 49 (c.5634 T > C; rs340781986), likely linked to a yet unidentified causal mutation, has been associated with the percentage of several fatty acids in crossed Iberian pigs^14,15^ and with lean and intramuscular fat content in Duroc^13^. We tested this mutation in our diallelic population along with two mutation in a putative enhancer in intron 24 of the gene (based on human regulatory data). In our Iberian population the synonymous rs340781986 SNP in exon 49 showed no effect on the fatty acid compositional data, confirming lack of causality. In contrast, one of the enhancer SNPs (12:38727103 G > A) was significantly associated with several SFA and MUFA fatty acids, but not with miristic content. Given the complexity of the gene, it might be challenging to demonstrate causality related to ACACA activity, and rule out other close polymorphisms in linkage disequilibrium with the tested mutations.

Taken together, the results presented here show that five genomic regions at SSC2, 7 and 12 regulate a relevant percentage of the genetic variance for fatty acid composition in Iberian pigs. It is relevant that most results presented here were specific of the Retinto variety (except the *CHGA* rs32866746 SNP, which was specific of Entrepelado). Retinto pigs represented 72 of the 208 pigs in the resource population and are characterised by an enhanced deposition of both IMF and backfat, which is higher in MUFA and oleic acid than in the other two varieties. These results most probably indicate the existence of Retinto/Entrepelado-specific causal mutations linked to the SNPs analysed here. This would agree with the segregation of genetic variety effects on IMF content and composition described in these Iberian varieties^6^. In the path to develop genetic markers useful for Iberian breeders, the results presented here support further study in the variety-specific genetic variability by means of targeted genome sequencing and global muscle transcriptomics.

## Materials and Methods

### Ethics approval

All animal procedures followed Spanish national guidelines and were approved by the Ethical Committee of Institut de Recerca i Tecnologia Agroalimentàries (IRTA). The animal experiments were carried out in accordance with the Guidelines for Animal Experiments.

### Experimental design

The animal material is described in detail by Ibáñez-Escriche *et al*.^6^. Briefly, a complete diallelic cross was generated at the Inga Food S.A. facilities involving three varieties of Iberian pigs (Torbiscal, Retinto, and Entrepelado) and their reciprocal crosses (Retinto × Entrepelado, Entrepelado × Retinto, Torbiscal × Retinto, Retinto × Torbiscal, Torbiscal × Entrepelado, and Entrepelado × Torbiscal). The 470 pigs generated were reared in standard intensive commercial conditions and had *ad libitum* access to typical commercial feed used in Iberian pig production^6^. The animals were slaughtered in a commercial slaughterhouse at an average of 340 days of age (SD 14.34) and 160.03 kg (SD 11.60) of live weight. At slaughter, a section of the *longissimus thoracis* muscle was collected for fatty acid content and composition determination. Samples of liver were also collected, snap-frozen in liquid nitrogen and stored at −80 °C for subsequent nucleic acid isolation.

### Fatty acid content and composition

For each animal, a sample of 200 g of *longissimus thoracis* muscle was minced and homogenized using a horizontal cutter mixer. Total IMF and its fatty acid profile were quantified according to the method described by O’Fallon^22^. Descriptive statistics and abbreviations for the traits analysed are included in Supplementary Table S1.

### DNA and RNA isolation

DNA was isolated from liver samples using standard protocols as described by Ros-Freixedes *et al*.^23^. RNA was isolated from liver and muscle samples from a subset of the pigs in the experiment by the acid phenol method^24^ using TRI-reagent (Sigma-Aldrich) and following the manufacturer’s instructions.

### Principal Components Analysis

Principal Components Analysis (PCA) with the SNP genotypes of the individuals passing the genotyping quality control was analysed witht *prcomp()* base function implemented in R and used to infer population structure in genetic data and to check for outliers.

### Genome-wide association analysis for meat quality traits

Genotyping was performed with the Porcine SNP60 BeadChip (Illumina), which contains 62,163 single nucleotide polymorphisms (SNPs). Quality control of data was carried out with the GenomeStudio software (Illumina), removing SNPs which either (i) mapped to the X chromosome, (ii) had a missing genotypes rate >5%, (iii) did not conform Hardy-Weinberg expectations (threshold set at a P-value ≤ 0.001), (iv) had a minor allele frequency below 0.05, (v) showed a GenCall score <0.15, (vi) had a call rate <95% or (vii) could not be mapped to the Sscrofa 11.1 version of the pig genome. After filtering the raw data, a GWAS was carried out with 20,580 SNPs from 342 individuals. Multi-SNP association analyses were performed with the Bayes B multiple marker regression (MMR) method^25^ implemented in the GenSel software^26^ under an additive genetic model. The kinship matrix was not included in the model because the makers fitted in the Bayesian MMR models capture the kinship structure^27^. We included the two first principal components to avoid possible patterns of population stratification. Nevertheless, Bayesian MMR models seems to be robust to population structure and to relationships^28^.

The statistical model assumed in this analysis was:

$${\bf{y}}={\bf{X}}{\bf{b}}+\sum _{j=1}^{k}{{\bf{z}}}_{j}{\alpha }_{j}{\delta }_{j}+{\bf{e}},$$

where **y** is the phenotype vector *n* x 1; **X** is the incidence matrix of systematic effects *n x p*; **b** is the systematic effect vector that includes the two first principal components (52% and 32% of the variance, respectively) as covariates, sex (2 levels) and batch (12 levels) effects and weight at slaughter as covariate for IMF trait, or the IMF content as covariate for fatty acid compositional data; **z**_j_ is the genotype vector *n* x 1 for each SNP in the *j* locus (*j* = 1 to *k*, where *k* is the SNP number); *α* is the allelic substitution vector for each SNP in the *j* locus; *δ* is a 0/1 random variable that indicates the absence or presence in the model (using π and 1-π as prior probabilities, respectively) of the *j* SNP for each iteration of the Markov-Monte Carlo chain; and **e** is the residual vector assumed to follow a normal distribution. Due to the limited number of animals in this study, the proportion of SNPs without effect in the model (*δ* = 0) was established at π = 0.995. Variance components calculated in a previous study^6^ were used as priors in the analyses. The statistical relevance of the associations between individual markers and traits was assessed by Bayes Factor (BF) for each locus^29^. Evidence of association was considered important for a BF > 5, strong when >10 and decisive when >100^30^. The proportion of variance explained conjointly by SNPs in not overlapped windows was estimated.

### Mapping SNPs to genes and pathways

Positional candidate genes up to 2 Mb around the markers with BF > 10 for at least one compositional trait were retrieved from Ensembl (EMBL-EBI) by Biomart. Functional annotation of the genes was performed with several tools with the aim to catalogue and identify genes with a potential role in fatty acid synthesis, transformation, storing, oxidation or transport. Tools included Enrichr^31^, which integrate several Gene Ontology and Pathway Analysis tools such as KEGG, Reactome and Wikipathways; Generic Gene Ontology Term Mapper (http://go.princeton.edu/cgi-bin/GOTermMapper); DAVID Bioinformatics Resources 6.8^32^; and the Go-Slim functional classification features of Panther (http://pantherdb.org/). More information can be found in Supplementary Table S3.

### Candidate gene analysis

The transcribed region and approximately 2 kb of 5’ flanking genomic sequence was amplified from 5 selected genes (*PLIN4*, *PLIN5*, *RXRB*, *PSRMB8*, *CHGA*). As the *ACACA* coding and promoter regions have been well characterised in Iberian and Duroc pigs, we selected a 1.8-kb fragment in intron 24 of the gene which corresponds to the sequence of an internal human *ACACA* enhancer described in the enhancer-to-gene associations in GeneHancer (embedded in GeneCards). Sequences were retrieved from the Sscrofa 10.2 assembly in Ensembl and primer pairs were designed using Primer3Plus^33^ (Supplementary Table S5). PCR conditions are described in detail Supplementary Table S6. Amplicons were purified with Exonuclease I and Shrimp alkaline phosphatase (ThermoFisher) according to the manufacturer’s intructions prior to Sanger sequencing. Electropherograms were assembled and processed with GeneStudio Pro (2.2.0.0) (GeneStudio, Inc.). Genotyping of polymorphisms was performed by high-resolution melting protocols in QuantStudio QS3 real-time thermocycler (LifeTechnolgies) with primers and conditions described in Supplementary Table S6.

### Association analysis

In order to perform association analysis between IMF content or FA composition and the genotyped SNPs in the five candidate genes, the following standard linear model was used:

$${y}_{ijklm}=\mu +batc{h}_{j}+Se{x}_{k}+Variet{y}_{l}+\beta \ast Weigh{t}_{ijklm}+Genotyp{e}_{m}+{e}_{ijklm}$$

where *y*_*ijklm*_ is the vector of phenotypic observations i.e. (IMF content; fatty acid composition) measured at the *longissimus thoracis* muscles of the *i*^*th*^ individual; *μ* is the population mean of each trait; *batch*_*j*_ is a systematic effect of the *j*^*th*^ fattening batch, with 11 categories; *Sex*_*k*_ is the sex (castrated male/female) of the *i*^*th*^ individual; β is the regression coefficient on the covariate weight at slaughter (for IMF) or IMF content (for fatty acid composition) (*Weight*_*i*__*jklm*_); *Variety*_*l*_ is the effect of the variety, with 3 levels (Retinto, Torbiscal, Entrepelado); *Genotype*_*m*_ is the genotype for each SNP, with three levels; and *e*_*ijklm*_ is the residual effect of the *i*^*th*^ individual. Additionally, the same model was applied to each individual Iberian variety, removing the *Variety*_*l*_ effect from the model. Differences between genotype means were assessed with a Tukey test (or a two-tail t-test when only two genotypes were available). Means were considered different at p-value <0.05.

## Supplementary information

Supplementary Information

## Data Availability

The results from all data analysed during this study are included or displayed in this article (and its Supplementary Information files, such as the Manhattan plots). Associations for individual SNPs with BF > 10 are tabulated (Supplementary Table S2) and regionally displayed (Supplementary Fig. S1). However, other individual SNP results may be made available from the corresponding author on request.
