# Supplementary material for: Five genomic regions have a major impact on fat composition in Iberian pigs
Source: Sci Rep. 2019 Feb 14;9:2031. doi: 10.1038/s41598-019-38622-7 (PMC6375979; doi:10.1038/s41598-019-38622-7)
Supplement: Supplementary file 1 — Supplementary Information [file 41598_2019_38622_MOESM1_ESM.pdf]

Supplementary information to:

Five genomic regions have a major impact on fat composition in Iberian pigs

R.N. Pena[1], J.L. Noguera[2], M.J. García-Santana[3], E. Gonzalez[4], J.F. Tejeda[4], R. Ros-Freixedes[1,5], N. Ibáñez-Escriche[6]

Supplementary Table S1. Means and standard error (±SE) by Iberian varieties and crosses for backfat thickness (BF) measured at 4th rib, intramuscular fat (IMF) of the *longissimus thoracis* and its main fatty acids, as percentages of total fatty acid content.

	Iberian resource population[1]								
	Purebred varieties			Crosses					
**Traits**	**EE**	**RR**	**TT**	**ER**	**ET**	**RE**	**RT**	**TE**	**TR**
**Miristic (C14:0)**	1.50 ± 0.02	1.50 ± 0.01	1.49 ± 0.01	1.58 ± 0.03	1.49 ± 0.03	1.55 ± 0.02	1.52 ± 0.01	1.50 ± 0.03	1.50 ± 0.02
**Palmitic (C16:0)**	26.04 ± 0.16	25.81 ± 0.23	26.30 ± 0.12	26.19 ± 0.46	26.24 ± 0.20	26.51 ± 0.26	25.90 ± 0.09	26.34 ± 0.26	25.68 ± 0.26
**Palmitoleic (C16:1, n-7)**	4.33 ± 0.09	4.36 ± 0.09	3.90 ± 0.04	4.56 ± 0.27	4.23 ± 0.06	4.05 ± 0.11	4.11 ± 0.04	4.11 ± 0.09	4.40 ± 0.10
**Stearic (C18:0)**	11.73 ± 0.14	11.27 ± 0.21	12.64 ± 0.09	11.29 ± 0.59	11.78 ± 0.13	11.81 ± 0.27	12.09 ± 0.09	12.04 ± 0.17	11.49 ± 0.22
**Oleic (C18:1, n-9)**	47.66 ± 0.22	48.22 ± 0.34	47.28 ± 0.19	48.26 ± 0.84	47.24 ± 0.21	47.85 ± 0.32	48.06 ± 0.16	47.78 ± 0.27	48.09 ± 0.35
**SFA**	39.66 ± 0.26	38.99 ± 0.43	40.86 ± 0.20	39.51 ± 0.97	40.05 ± 0.29	40.32 ± 0.49	39.89 ± 0.17	40.34 ± 0.33	39.07 ± 0.46
**MUFA**	53.00 ± 0.26	53.70 ± 0.42	52.27 ± 0.21	53.91 ± 1.08	52.62 ± 0.22	53.03 ± 0.37	53.21 ± 0.18	52.99 ± 0.30	53.59 ± 0.41
**PUFA**	7.34 ± 0.25	7.25 ± 0.13	6.83 ± 0.11	6.58 ± 0.39	7.33 ± 0.31	6.65 ± 0.39	6.88 ± 0.08	6.66 ± 0.33	7.25 ± 0.20
**IMF, % muscle**	7.13 ± 0.37	9.49 ± 0.40	7.73 ± 0.30	9.16 ± 0.81	6.18 ± 0.47	8.44 ± 0.75	8.29 ± 0.19	7.18 ± 0.49	8.56 ± 0.46
**BF, cm**	7.59 ± 0.24	9.27 ± 0.16	9.21 ± 0.13	7.31 ± 0.29	7.36 ± 0.16	8.54 ± 0.33	9.50 ± 0.08	7.85 ± 0.23	9.18 ± 0.15

[1]E – Entrepelado; T – Torbiscal; R – Retinto. [2]SFA – saturated fatty acids; MUFA – monounsaturated fatty acids; PUFA – polyunsaturated fatty acids.

Supplementary Table S2. Genome regions strongly associated (Bayes factor (BF) >10) with *longissimus thoracis* fatty acid content and composition in Iberian pigs. A total of 32 regions were identified involving 65 distinct SNPs.

SSC[1]	Region[2]	n. SNP	Marker with highest BF	Trait[3]	BF	%GV[4]
1	109.6	1	ALGA0119806	MUFA/SFA	15.03	0.61
	173.2-173.8	4	ALGA0007029	C14:0	11.53	4.13
2	20.0-20.9	1	ALGA0109169	PUFA	81.97	4.95
	75.0-75.6	2	MARC0048160	C16:1	624.06	14.44
				MUFA/SFA	14.98	0.37
	84.6	1	MARC0044595	MUFA	11.66	0.64
	90.4-97.9	4	DRGA0003183	C16:1	28.36	2.21
				C18:1	10.97	1.34
				MUFA	13.45	2.25
	100.1-106.0	7	ALGA0014789	C18:1	10.7	1.08
				C16:1	52.33	4.07
				MUFA	12.63	1.33
				MUFA/SFA	24.32	3.3
	115.1-115.7	3	MARC0058544	C14:0	16.54	1.35
	149.4	1	MARC0055611	C14:0	12.86	0.31
3	30.6	1	ALGA0018401	C16:1	12.43	0.25
				MUFA/SFA	14.06	0.32
	100.4-110.2	3	ALGA0020416	C14:0	10.11	0.5
				C18:1	12.11	0.91
				MUFA/SFA	10.93	0.37
4	123.0-127.9	3	MARC0014258	C14:0	18.17	0.51
				MUFA/SFA	31	1.94
6	3.0-4.0	2	ALGA0103513	C16:1	23.91	0.95
	11.9	1	H3GA0017544	PUFA	10.79	0.5
	18.5	1	MARC0026696	C14:0	11.8	0.36
	94.1-94.5	2	ALGA0035978	C14:0	13.17	0.78
7	25.0-25.9	2	H3GA0020505	C18:1	20.12	2.1
				MUFA/SFA	13.15	0.77
	103.3-105.6	3	MARC0033066	C16:1	11.8	1.81
	113.2-115.2	2	INRA0028453	C18:1	117.66	10.4
				MUFA	174.95	15.48
				MUFA/SFA	117.36	6.13
8	13.7-17.4	2	DRGA0008365	PUFA	37.36	3.26
	29.4-40.1	3	ASGA0099168	C14:0	15.45	0.42
				C16:1	17.16	1.21
	116.2	1	ASGA0039708	C14:0	10.39	0.28
9	12.5	1	ALGA0107040	C14:0	33.78	0.88
10	0.56	1	ALGA0056248	MUFA/SFA	11.62	0.36
12	36.9-38.9	9	ALGA0054436	C14:0	77.66	17.13

	56	1	ALGA0122482	C16:1	10.46	0.8
14	137.5	1	DRGA0014761	IMF	16.52	1.42
15	112.3	1	ASGA0070398	C14:0	11.57	0.48
	122.7	1	H3GA0045110	C16:1	21.12	0.76
16	41.4	1	ALGA0090494	C14:0	13.63	0.61
	73.2-78.3	2	MARC0095782	C14:0	14.22	0.44
				C16:1	25.66	0.84
18	37.67	1	MARC0025286	C16:1	11.01	0.22

[1]SSC – *Sus scrofa* chromosome number.
[2]Range and location (in Mb) refer to pig genome assembly 11.1 coordinates.
[3]Traits: Fatty acids, in %; SFA – saturated fatty acid content; MUFA – monounsaturated fatty acid content; MUFA – monounsaturated fatty acid content; MUFA/SFA – MUFA to SFA ratio; IMF – intramuscular fat, %.
[4]%GV - percentage of the genetic variance explained by the SNPs in each region.

Supplementary Table S3. Functional classification of positional candidate genes.

Sheet 1: Full list of 974 genes located up to 1 Mb from the main GWAS peaks (Bayes factor >10).

Sheet 2: Results of analysis in gene ontology and pathway participation with EnrichR, David, Panther and GOTermMapper tools.

Sheet3: List of the 112 genes functionally catalogued as related to the metabolism, transport or storage of fatty acids.

Supplementary Table S3. Sheet 1: Full list of 582 genes located up to 1 Mb from the main GWAS peaks (Bayes factor >10).

hgnc_symbol	ensembl_gene_id	Entrez_id	chromosome name	start position	end position	gene_biotype	ApprovedName
TRIP4	ENSSSCG00000004552	100153164	1	107,585,987	107,655,214	protein_coding	thyroid hormone receptor interactor 4
CSNK1G1	ENSSSCG00000004558	100157196	1	107,690,006	107,884,050	protein_coding	casein kinase 1 gamma 1
PPIB	ENSSSCG00000034137	100737887	1	107,886,612	107,897,098	protein_coding	peptidylprolyl isomerase B
SNX22	ENSSSCG00000037275	100624664	1	107,891,698	107,897,529	protein_coding	sorting nexin 22
SNX1	ENSSSCG00000004556	100153561	1	107,909,671	107,952,265	protein_coding	sorting nexin 1
CIAO2A	ENSSSCG00000034124	100156836	1	107,951,646	107,969,298	protein_coding	cytosolic iron-sulfur assembly component 2A
DAPK2	ENSSSCG00000021588	100155578	1	107,997,285	108,132,782	protein_coding	death associated protein kinase 2
HERC1	ENSSSCG00000004561	100153537	1	108,205,794	108,403,681	protein_coding	HECT and RLD domain containing E3 ubiquitin protein ligase family member 1
USP3	ENSSSCG00000028737	100624494	1	108,414,360	108,515,548	protein_coding	ubiquitin specific peptidase 3
CA12	ENSSSCG00000004565	100152749	1	108,649,274	108,708,185	protein_coding	carbonic anhydrase 12
RAB8B	ENSSSCG00000026571	100153972	1	108,764,304	108,840,218	protein_coding	RAB8B, member RAS oncogene family
LACTB	ENSSSCG00000004569	100152773	1	108,909,702	108,924,511	protein_coding	lactamase beta
TPM1	ENSSSCG00000004570	100037999	1	108,985,778	109,019,044	protein_coding	tropomyosin 1
TLN2	ENSSSCG00000024088	100156660	1	109,208,667	109,584,733	protein_coding	talin 2
VPS13C	ENSSSCG00000036431	100625505	1	109,931,251	110,104,257	protein_coding	vacuolar protein sorting 13 homolog C
RORA	ENSSSCG00000004576	100156637	1	111,375,423	111,477,664	protein_coding	RAR related orphan receptor A
ICE2	ENSSSCG00000004577	100157039	1	111,489,168	111,561,911	protein_coding	interactor of little elongation complex ELL subunit 2
ANXA2	ENSSSCG00000004578	406192	1	111,588,306	111,635,100	protein_coding	annexin A2
LRFN5	ENSSSCG00000004990	100156782	1	171,906,198	172,177,720	protein_coding	leucine rich repeat and fibronectin type III domain containing 5
C14orf28	ENSSSCG00000030679	100736797	1	175,145,913	175,156,595	protein_coding	chromosome 14 open reading frame 28
KLHL28	ENSSSCG00000004996	100156355	1	175,164,040	175,180,525	protein_coding	kelch like family member 28
TOGARAM1	ENSSSCG00000027765	100513707	1	175,198,145	175,274,636	protein_coding	TOG array regulator of axonemal microtubules 1
PRPF39	ENSSSCG00000004997	100153928	1	175,285,243	175,317,242	protein_coding	pre-mRNA processing factor 39
FANCM	ENSSSCG00000005000	100155917	1	175,334,141	175,445,650	protein_coding	FA complementation group M
MIS18BP1	ENSSSCG00000004999	100620483	1	175,399,406	175,448,354	protein_coding	MIS18 binding protein 1
ACCSL	ENSSSCG00000013283	100521486	2	18,271,075	18,448,219	protein_coding	1-aminocyclopropane-1-carboxylate synthase homolog (inactive) like
C11orf96	ENSSSCG00000039182	110259563	2	18,392,916	18,393,633	protein_coding	chromosome 11 open reading frame 96
ALKBH3	ENSSSCG00000040937	100624169	2	18,408,992	18,448,167	protein_coding	alkB homolog 3, alpha-ketoglutaratedependent dioxygenase
HSD17B12	ENSSSCG00000021739	100312972	2	18,458,890	18,611,765	protein_coding	hydroxysteroid 17-beta dehydrogenase 12
TTC17	ENSSSCG00000013284	100739017	2	18,807,530	18,954,667	protein_coding	tetratricopeptide repeat domain 17
API5	ENSSSCG00000013285	100522006	2	18,962,271	18,994,240	protein_coding	apoptosis inhibitor 5
LRRC4C	ENSSSCG00000013286	106509370	2	21,458,998	21,633,681	protein_coding	leucine rich repeat containing 4C
ZNRF4	ENSSSCG00000013519	100511549	2	73,484,988	73,486,265	protein_coding	zinc and ring finger 4
PTPRS	ENSSSCG00000028612	106509452	2	73,622,409	73,679,458	protein_coding	protein tyrosine phosphatase, receptor type S
KDM4B	ENSSSCG00000024570	100738761	2	73,746,494	73,889,865	protein_coding	lysine demethylase 4B
UHRF1	ENSSSCG00000013517	100511003	2	73,897,310	73,931,916	protein_coding	ubiquitin like with PHD and ring finger domains 1
ARRDC5	ENSSSCG00000037183	110259356	2	73,935,530	73,950,980	protein_coding	arrestin domain containing 5
PLIN3	ENSSSCG00000038954	595103	2	73,969,909	73,997,400	protein_coding	perilipin 3
TICAM1	ENSSSCG00000024771	100623776	2	74,004,323	74,021,596	protein_coding	toll like receptor adaptor molecule 1
FEM1A	ENSSSCG00000037153	100625091	2	74,042,989	74,044,983	protein_coding	fem-1 homolog A
MIR7-3	ENSSSCG00000025936	NA	2	74,066,290	74,066,371	miRNA	microRNA 7-3
DPP9	ENSSSCG00000026063	100621098	2	74,134,733	74,179,739	protein_coding	dipeptidyl peptidase 9
MYDGF	ENSSSCG00000034898	110259358	2	74,185,227	74,202,304	protein_coding	myeloid derived growth factor
TNFAIP8L1	ENSSSCG00000034615	110259359	2	74,203,757	74,220,724	protein_coding	TNF alpha induced protein 8 like 1
SEMA6B	ENSSSCG00000035176	110259360	2	74,223,744	74,300,462	protein_coding	semaphorin 6B
LRG1	ENSSSCG00000013514	100524848	2	74,300,664	74,304,559	protein_coding	leucine rich alpha-2-glycoprotein 1
PLIN5	ENSSSCG00000013513	100142670	2	74,304,634	74,314,315	protein_coding	perilipin 5
PLIN4	ENSSSCG00000013512	100524667	2	74,318,628	74,331,930	protein_coding	perilipin 4
HDGFL2	ENSSSCG00000013511	100526035	2	74,331,909	74,357,040	protein_coding	HDGF like 2
UBXN6	ENSSSCG00000013510	100524481	2	74,357,517	74,403,098	protein_coding	UBX domain protein 6
CHAF1A	ENSSSCG00000013509	100524302	2	74,381,075	74,411,659	protein_coding	chromatin assembly factor 1 subunit A
SH3GL1	ENSSSCG00000013508	100523947	2	74,414,448	74,445,447	protein_coding	SH3 domain containing GRB2 like 1, endophilin A2
MPND	ENSSSCG00000013507	100524132	2	74,441,299	74,455,920	protein_coding	MPN domain containing
STAP2	ENSSSCG00000013506	100523772	2	74,462,279	74,471,534	protein_coding	signal transducing adaptor family member 2
FSD1	ENSSSCG00000013505	100523485	2	74,468,739	74,485,807	protein_coding	fibronectin type III and SPRY domain containing 1
TMIGD2	ENSSSCG00000013504	102164394	2	74,487,429	74,497,310	protein_coding	transmembrane and immunoglobulin domain containing 2
SHD	ENSSSCG00000013503	100523303	2	74,497,351	74,504,659	protein_coding	Src homology 2 domain containing transforming protein D
YJU2	ENSSSCG00000013499	100522786	2	74,509,883	74,528,280	protein_coding	YJU2 splicing factor homolog
EBI3	ENSSSCG00000013498	100522599	2	74,530,730	74,539,780	protein_coding	Epstein-Barr virus induced 3
ANKRD24	ENSSSCG00000013497	100525327	2	74,539,688	74,564,902	protein_coding	ankyrin repeat domain 24
CREB3L3	ENSSSCG00000013501	100522979	2	74,577,004	74,601,829	protein_coding	cAMP responsive element binding protein 3 like 3
ZBTB7A	ENSSSCG00000029236	100625057	2	74,678,476	74,696,371	protein_coding	zinc finger and BTB domain containing 7A
PIAS4	ENSSSCG00000022066	100624971	2	74,699,939	74,729,789	protein_coding	protein inhibitor of activated STAT 4
EEF2	ENSSSCG00000025675	100624328	2	74,746,452	74,759,082	protein_coding	eukaryotic translation elongation factor 2
DAPK3	ENSSSCG00000032170	100624415	2	74,758,979	74,775,396	protein_coding	death associated protein kinase 3
NMRK2	ENSSSCG00000040860	100624588	2	74,782,995	74,791,449	protein_coding	nicotinamide riboside kinase 2
ATCAY	ENSSSCG00000026754	100626830	2	74,800,125	74,838,679	protein_coding	ATCAY, caytaxin
ZFR2	ENSSSCG00000013495	100521543	2	74,846,043	74,900,055	protein_coding	zinc finger RNA binding protein 2
MATK	ENSSSCG00000013494	110255242	2	74,875,996	74,918,971	protein_coding	megakaryocyte-associated tyrosine kinase
RAX2	ENSSSCG00000032429	100626562	2	74,922,415	74,928,220	protein_coding	retina and anterior neural fold homeobox 2
MRPL54	ENSSSCG00000024242	100521888	2	74,925,066	74,928,197	protein_coding	mitochondrial ribosomal protein L54
APBA3	ENSSSCG00000013492	100521713	2	74,929,342	74,945,290	protein_coding	amyloid beta precursor protein binding family A member 3
TJP3	ENSSSCG00000013491	100626455	2	74,936,613	74,971,531	protein_coding	tight junction protein 3
PIP5K1C	ENSSSCG00000013490	100521362	2	74,977,266	75,046,895	protein_coding	phosphatidylinositol-4-phosphate 5-kinase type 1 gamma
CACTIN	ENSSSCG00000032085	100521180	2	75,049,315	75,068,260	protein_coding	cactin, spliceosome C complex subunit
TBXA2R	ENSSSCG00000033759	110259364	2	75,068,673	75,081,715	protein_coding	thromboxane A2 receptor
GIPC3	ENSSSCG00000036190	110259363	2	75,082,053	75,089,810	protein_coding	GIPC PDZ domain containing family member 3
HMG20B	ENSSSCG00000040421	110259365	2	75,092,663	75,098,562	protein_coding	high mobility group 20B
MFSD12	ENSSSCG00000026169	100519240	2	75,107,529	75,120,705	protein_coding	major facilitator superfamily domain containing 12
C19orf71	ENSSSCG00000013482	100519760	2	75,118,851	75,121,827	protein_coding	chromosome 19 open reading frame 71
FZR1	ENSSSCG00000013481	497234	2	75,122,595	75,178,272	protein_coding	fizzy and cell division cycle 20 related 1

DOHH	ENSSSCG00000013478	100519411	2	75,164,537	75,175,161	protein_coding	deoxyhypusine hydroxylase
NFIC	ENSSSCG00000036537	100737330	2	75,193,170	75,263,372	protein_coding	nuclear factor I C
CELF5	ENSSSCG00000013476	100518716	2	75,314,834	75,364,165	protein_coding	CUGBP Elav-like family member 5
NCLN	ENSSSCG00000013475	100518176	2	75,372,606	75,390,545	protein_coding	nicalin
S1PR4	ENSSSCG00000013474	100517997	2	75,396,170	75,397,483	protein_coding	sphingosine-1-phosphate receptor 4
GNA15	ENSSSCG00000013473	100518537	2	75,408,726	75,429,903	protein_coding	G protein subunit alpha 15
AES	ENSSSCG00000032265	110259367	2	75,492,233	75,501,542	protein_coding	amino-terminal enhancer of split
TLE2	ENSSSCG00000028995	100622728	2	75,515,115	75,540,289	protein_coding	transducin like enhancer of split 2
ZNF555	ENSSSCG00000013469	110259371	2	75,811,392	75,836,442	protein_coding	zinc finger protein 555
ZNF554	ENSSSCG00000038635	NA	2	75,835,685	75,845,552	protein_coding	zinc finger protein 554
SGTA	ENSSSCG00000013467	100515997	2	75,879,058	75,896,277	protein_coding	small glutamine rich tetratricopeptide repeat containing alpha
SLC39A3	ENSSSCG00000033968	100515818	2	75,908,492	75,916,872	protein_coding	solute carrier family 39 member 3
DIRAS1	ENSSSCG00000038013	100517263	2	75,921,153	75,927,234	protein_coding	DIRAS family GTPase 1
GNG7	ENSSSCG00000033949	100621275	2	76,049,088	76,083,558	protein_coding	G protein subunit gamma 7
GADD45B	ENSSSCG00000022689	100621090	2	76,106,916	76,111,479	protein_coding	growth arrest and DNA damage inducible beta
LMNB2	ENSSSCG00000026784	100620983	2	76,125,920	76,146,733	protein_coding	lamin B2
TIMM13	ENSSSCG00000022757	100620885	2	76,146,950	76,148,593	protein_coding	translocase of inner mitochondrial membrane 13
TMPRSS9	ENSSSCG00000028363	100621666	2	76,158,045	76,227,865	protein_coding	transmembrane serine protease 9
SPPL2B	ENSSSCG00000013464	100515648	2	76,229,177	76,247,101	protein_coding	signal peptide peptidase like 2B
LSM7	ENSSSCG00000013463	NA	2	76,246,933	76,251,695	protein_coding	LSM7 homolog, U6 small nuclear RNA and mRNA degradation associated
LINGO3	ENSSSCG00000013462	100515299	2	76,276,617	76,278,395	protein_coding	leucine rich repeat and Ig domain containing 3
PEAK3	ENSSSCG00000013461	100515125	2	76,285,882	76,291,290	protein_coding	PEAK family member 3
DOT1L	ENSSSCG00000013457	100738665	2	76,339,442	76,403,354	protein_coding	DOT1 like histone lysine methyltransferase
SF3A2	ENSSSCG00000039757	100516539	2	76,404,452	76,419,202	protein_coding	splicing factor 3a subunit 2
AMH	ENSSSCG00000035812	397578	2	76,414,427	76,418,315	protein_coding	anti-Mullerian hormone
IZUMO4	ENSSSCG00000013455	100514821	2	76,472,231	76,475,169	protein_coding	IZUMO family member 4
MOB3A	ENSSSCG00000032366	110259373	2	76,475,392	76,495,931	protein_coding	MOB kinase activator 3A
MKNK2	ENSSSCG00000013448	100517077	2	76,497,190	76,522,877	protein_coding	MAP kinase interacting serine/threonine kinase 2
BTBD2	ENSSSCG00000013444	100513521	2	76,538,439	76,559,919	protein_coding	BTB domain containing 2
CSNK1G2	ENSSSCG00000013445	100525276	2	76,573,985	76,609,575	protein_coding	casein kinase 1 gamma 2
SCAMP4	ENSSSCG00000013446	100738966	2	76,614,984	76,634,274	protein_coding	secretory carrier membrane protein 4
ADAT3	ENSSSCG00000038810	102159223	2	76,628,634	76,634,262	protein_coding	adenosine deaminase, tRNA specific 3
ABHD17A	ENSSSCG00000013442	100524666	2	76,658,068	76,666,559	protein_coding	abhydrolase domain containing 17A
REXO1	ENSSSCG00000040624	100525449	2	76,694,166	76,721,730	protein_coding	RNA exonuclease 1 homolog
ATP8B3	ENSSSCG00000013440	100513137	2	76,722,254	76,746,255	protein_coding	ATPase phospholipid transporting 8B3
ONECUT3	ENSSSCG00000013439	100512947	2	76,751,070	76,769,812	protein_coding	one cut homeobox 3
TCF3	ENSSSCG00000013437	100524070	2	76,859,074	76,898,350	protein_coding	transcription factor 3
MBD3	ENSSSCG00000013435	100523889	2	76,913,905	76,923,431	protein_coding	methyl-CpG binding domain protein 3
MEX3D	ENSSSCG00000021750	100512764	2	76,929,290	76,939,594	protein_coding	mex-3 RNA binding family member D
ADAMTSL5	ENSSSCG00000013433	100512586	2	76,966,739	76,975,544	protein_coding	ADAMTS like 5
REEP6	ENSSSCG00000026387	100626712	2	76,984,234	76,988,761	protein_coding	receptor accessory protein 6
PCSK4	ENSSSCG00000024379	100626523	2	76,989,743	76,997,766	protein_coding	proprotein convertase subtilisin/kexin type 4
C19orf25	ENSSSCG00000022265	100625377	2	76,998,977	77,003,327	protein_coding	chromosome 19 open reading frame 25
DAZAP1	ENSSSCG00000022528	100625005	2	77,048,896	77,070,780	protein_coding	DAZ associated protein 1
GAMT	ENSSSCG00000023142	100625146	2	77,075,894	77,079,759	protein_coding	guanidinoacetate N-methyltransferase
NDUFS7	ENSSSCG00000024144	100624738	2	77,076,573	77,087,126	protein_coding	NADH:ubiquinone oxidoreductase core subunit S7
MUM1	ENSSSCG00000013426	100511862	2	77,086,985	77,111,182	protein_coding	melanoma associated antigen (mutated) 1
EFNA2	ENSSSCG00000038701	100512036	2	77,164,605	77,179,137	protein_coding	ephrin A2
CIRBP	ENSSSCG00000013427	100522851	2	77,185,308	77,194,324	protein_coding	cold inducible RNA binding protein
C19orf24	ENSSSCG00000028040	100523046	2	77,185,309	77,189,514	protein_coding	chromosome 19 open reading frame 24
MIDN	ENSSSCG00000013432	100512395	2	77,204,017	77,211,864	protein_coding	midnolin
ATP5F1D	ENSSSCG00000035532	100523423	2	77,212,625	77,218,645	protein_coding	ATP synthase F1 subunit delta
STK11	ENSSSCG00000013429	100512213	2	77,231,796	77,252,075	protein_coding	serine/threonine kinase 11
SBNO2	ENSSSCG00000030042	100623993	2	77,275,013	77,323,937	protein_coding	strawberry notch homolog 2
POLR2E	ENSSSCG00000021472	100623333	2	77,330,750	77,341,927	protein_coding	RNA polymerase II subunit E
ARHGAP45	ENSSSCG00000027565	100623239	2	77,342,155	77,355,724	protein_coding	Rho GTPase activating protein 45
ABCA7	ENSSSCG00000023121	100623892	2	77,357,212	77,375,170	protein_coding	ATP binding cassette subfamily A member 7
CNN2	ENSSSCG00000039542	397565	2	77,357,214	77,385,576	protein_coding	calponin 2
TMEM259	ENSSSCG00000027779	100622984	2	77,393,611	77,402,262	protein_coding	transmembrane protein 259
GRIN3B	ENSSSCG00000029635	100623799	2	77,402,369	77,411,878	protein_coding	glutamate ionotropic receptor NMDA type subunit 3B
WDR18	ENSSSCG00000013423	100622895	2	77,413,674	77,423,368	protein_coding	WD repeat domain 18
ARID3A	ENSSSCG00000037642	100622803	2	77,431,089	77,465,009	protein_coding	AT-rich interaction domain 3A
FOXD1	ENSSSCG00000035878	110259399	2	82,607,848	82,609,248	protein_coding	forkhead box D1
ANKRA2	ENSSSCG00000014070	100515129	2	82,698,002	82,715,345	protein_coding	ankyrin repeat family A member 2
ARHGEF28	ENSSSCG00000028317	100515652	2	82,761,521	83,102,121	protein_coding	Rho guanine nucleotide exchange factor 28
ENC1	ENSSSCG00000014072	100517459	2	83,685,443	83,687,254	protein_coding	ectodermal-neural cortex 1
GFM2	ENSSSCG00000014074	100517638	2	83,779,427	83,843,827	protein_coding	G elongation factor mitochondrial 2
FAM169A	ENSSSCG00000014076	100518001	2	83,852,220	83,927,154	protein_coding	family with sequence similarity 169 member A
GCNT4	ENSSSCG00000029710	100518180	2	84,105,655	84,135,729	protein_coding	glucosaminyl (N-acetyl) transferase 4, core 2
COL4A3BP	ENSSSCG00000014081	100518474	2	84,417,409	84,537,369	protein_coding	collagen type IV alpha 3 binding protein
POLK	ENSSSCG00000014082	100518650	2	84,531,102	84,690,674	protein_coding	DNA polymerase kappa
ANKDD1B	ENSSSCG00000014083	100518825	2	84,621,347	84,689,341	protein_coding	ankyrin repeat and death domain containing 1B
POC5	ENSSSCG00000014084	100519012	2	84,690,127	84,730,954	protein_coding	POC5 centriolar protein
SV2C	ENSSSCG00000034062	100517268	2	84,996,261	85,235,718	protein_coding	synaptic vesicle glycoprotein 2C
IQGAP2	ENSSSCG00000014088	100521253	2	85,293,937	85,633,641	protein_coding	IQ motif containing GTPase activating protein 2
F2RL2	ENSSSCG00000037582	100519355	2	85,535,662	85,542,145	protein_coding	coagulation factor II thrombin receptor like 2
F2RL1	ENSSSCG00000014091	100519703	2	85,732,082	85,747,621	protein_coding	F2R like trypsin receptor 1
S100Z	ENSSSCG00000014092	100519888	2	85,767,571	85,770,957	protein_coding	S100 calcium binding protein Z
CRHBP	ENSSSCG00000014093	100337682	2	85,814,352	85,831,182	protein_coding	corticotropin releasing hormone binding protein
ZBED3	ENSSSCG00000014095	NA	2	85,948,934	85,949,571	protein_coding	zinc finger BED-type containing 3
WDR41	ENSSSCG00000014098	100520548	2	86,322,955	86,380,571	protein_coding	WD repeat domain 41
OTP	ENSSSCG00000014099	100520724	2	86,529,349	86,537,328	protein_coding	orthopedia homeobox
TBCA	ENSSSCG00000014100	100523593	2	86,581,240	86,664,898	protein_coding	tubulin folding cofactor A
MTX3	ENSSSCG00000014116	100512828	2	88,667,294	88,680,947	protein_coding	metaxin 3
THBS4	ENSSSCG00000014117	100513005	2	88,731,122	88,780,596	protein_coding	thrombospondin 4
SERINC5	ENSSSCG00000014119	100624743	2	88,843,020	88,961,132	protein_coding	serine incorporator 5
SPZ1	ENSSSCG00000014122	100624840	2	89,014,940	89,016,061	protein_coding	spermatogenic leucine zipper 1

ZFYVE16	ENSSSCG00000014121	100511806	2	89,045,085	89,107,255	protein_coding	zinc finger FYVE-type containing 16
FAM151B	ENSSSCG00000014123	100511985	2	89,112,321	89,144,266	protein_coding	family with sequence similarity 151 member B
ANKRD34B	ENSSSCG00000014124	100513200	2	89,155,769	89,171,325	protein_coding	ankyrin repeat domain 34B
MSH3	ENSSSCG00000014126	100512158	2	89,255,554	89,452,223	protein_coding	mutS homolog 3
RASGRF2	ENSSSCG00000014127	100515363	2	89,528,122	89,790,718	protein_coding	Ras protein specific guanine nucleotide releasing factor 2
CKMT2	ENSSSCG00000014128	733602	2	89,796,474	89,832,990	protein_coding	creatine kinase, mitochondrial 2
ACOT12	ENSSSCG00000036095	100738062	2	89,855,166	89,905,197	protein_coding	acyl-CoA thioesterase 12
SSBP2	ENSSSCG00000035757	100515711	2	89,957,682	90,263,803	protein_coding	single stranded DNA binding protein 2
ATG10	ENSSSCG00000025210	100462744	2	90,437,020	90,665,380	protein_coding	autophagy related 10
VCAN	ENSSSCG00000014136	100514895	2	91,287,931	91,811,674	protein_coding	versican
HAPLN1	ENSSSCG00000014137	445513	2	91,857,498	91,935,490	protein_coding	hyaluronan and proteoglycan link protein 1
EDIL3	ENSSSCG00000026780	100516065	2	92,123,346	92,554,322	protein_coding	EGF like repeats and discoidin domains 3
RASA1	ENSSSCG00000014146	100627579	2	94,879,210	94,994,624	protein_coding	RAS p21 protein activator 1
CCNH	ENSSSCG00000014147	100310796	2	94,979,956	95,018,726	protein_coding	cyclin H
TMEM161B	ENSSSCG00000014148	100518540	2	95,579,436	95,700,822	protein_coding	transmembrane protein 161B
MEF2C	ENSSSCG00000014149	733590	2	96,122,044	96,296,902	protein_coding	myocyte enhancer factor 2C
CETN3	ENSSSCG00000014151	574052	2	97,529,763	97,593,898	protein_coding	centrin 3
MBLAC2	ENSSSCG00000014152	100519704	2	97,632,419	97,648,147	protein_coding	metallo-beta-lactamase domain containing 2
POLR3G	ENSSSCG00000026233	100519356	2	97,648,267	97,688,701	protein_coding	RNA polymerase III subunit G
LYSMD3	ENSSSCG00000024193	100519537	2	97,689,376	97,703,742	protein_coding	LysM domain containing 3
ADGRV1	ENSSSCG00000035025	100519889	2	97,775,485	98,002,203	protein_coding	adhesion G protein-coupled receptor V1
ARRDC3	ENSSSCG00000014156	100520233	2	98,430,457	98,444,661	protein_coding	arrestin domain containing 3
NR2F1	ENSSSCG00000014157	100621341	2	100,447,904	100,457,904	protein_coding	nuclear receptor subfamily 2 group F member 1
FAM172A	ENSSSCG00000027608	100521436	2	100,456,787	100,893,668	protein_coding	family with sequence similarity 172 member A
POU5F2	ENSSSCG00000036467	100739766	2	100,604,220	100,605,476	protein_coding	POU domain class 5, transcription factor 2
SLF1	ENSSSCG00000014163	100522125	2	101,313,109	101,375,607	protein_coding	SMC5-SMC6 complex localization factor 1
MCTP1	ENSSSCG00000025286	100522480	2	101,384,911	101,739,336	protein_coding	multiple C2 and transmembrane domain containing 1
FAM81B	ENSSSCG00000026175	100522665	2	102,009,998	102,067,727	protein_coding	family with sequence similarity 81 member B
ARSK	ENSSSCG00000014139	100627205	2	102,164,091	102,209,755	protein_coding	arylsulfatase family member K
GPR150	ENSSSCG00000014140	100516597	2	102,222,131	102,223,426	protein_coding	G protein-coupled receptor 150
RFESD	ENSSSCG00000014141	100516784	2	102,243,472	102,254,255	protein_coding	Rieske Fe-S domain containing
RHOBTB3	ENSSSCG00000029805	100523594	2	102,334,820	102,491,856	protein_coding	Rho related BTB domain containing 3
ELL2	ENSSSCG00000014168	100523777	2	102,519,577	102,591,421	protein_coding	elongation factor for RNA polymerase II 2
LIX1	ENSSSCG00000023396	100511371	2	103,744,330	103,792,382	protein_coding	limb and CNS expressed 1
RGMB	ENSSSCG00000014176	NA	2	105,047,677	105,067,850	protein_coding	repulsive guidance molecule BMP co-receptor b
CHD1	ENSSSCG00000014177	100525390	2	105,113,163	105,203,206	protein_coding	chromodomain helicase DNA binding protein 1
FAM174A	ENSSSCG00000014179	100525564	2	106,555,488	106,584,584	protein_coding	family with sequence similarity 174 member A
SLCO4C1	ENSSSCG00000014182	100737557	2	107,817,177	107,887,399	protein_coding	solute carrier organic anion transporter family member 4C1
SLCO6A1	ENSSSCG00000014183	100512269	2	107,922,002	108,020,311	protein_coding	solute carrier organic anion transporter family member 6A1
FBXL17	ENSSSCG00000014190	100513341	2	113,297,645	113,480,210	protein_coding	F-box and leucine rich repeat protein 17
FER	ENSSSCG00000027074	100513914	2	113,822,532	114,206,930	protein_coding	FER tyrosine kinase
PJA2	ENSSSCG00000022845	100514278	2	114,319,727	114,393,945	protein_coding	praja ring finger ubiquitin ligase 2
MAN2A1	ENSSSCG00000014195	100514631	2	114,675,917	114,853,524	protein_coding	mannosidase alpha class 2A member 1
TMEM232	ENSSSCG00000014196	100515882	2	115,275,700	115,523,659	protein_coding	transmembrane protein 232
SLC25A46	ENSSSCG00000014197	100516066	2	115,529,496	115,555,452	protein_coding	solute carrier family 25 member 46
TSLP	ENSSSCG00000014198	NA	2	115,833,657	115,837,535	protein_coding	thymic stromal lymphopoietin
WDR36	ENSSSCG00000014199	100627271	2	115,835,104	115,907,635	protein_coding	WD repeat domain 36
CAMK4	ENSSSCG00000037019	100515364	2	116,001,146	116,229,759	protein_coding	calcium/calmodulin dependent protein kinase IV
STARD4	ENSSSCG00000031518	100233203	2	116,234,587	116,257,041	protein_coding	StAR related lipid transfer domain containing 4
DCP2	ENSSSCG00000014204	100516785	2	116,743,012	116,804,633	protein_coding	decapping mRNA 2
REEP5	ENSSSCG00000022048	100516959	2	116,845,571	116,886,358	protein_coding	receptor accessory protein 5
SRP19	ENSSSCG00000026230	100517270	2	116,890,852	116,901,444	protein_coding	signal recognition particle 19
LARS	ENSSSCG00000014411	100525981	2	147,480,336	147,547,298	protein_coding	leucyl-tRNA synthetase
POU4F3	ENSSSCG00000014412	100510953	2	147,693,014	147,694,345	protein_coding	POU class 4 homeobox 3
TCERG1	ENSSSCG00000014416	100621703	2	147,792,034	147,852,461	protein_coding	transcription elongation regulator 1
GPR151	ENSSSCG00000014415	100621805	2	147,856,604	147,857,869	protein_coding	G protein-coupled receptor 151
PPP2R2B	ENSSSCG00000028976	397089	2	147,937,442	148,411,731	protein_coding	protein phosphatase 2 regulatory subunit Bbeta
STK32A	ENSSSCG00000014418	100622368	2	148,565,634	148,656,043	protein_coding	serine/threonine kinase 32A
DPYSL3	ENSSSCG00000036549	100525453	2	148,662,051	148,855,649	protein_coding	dihydropyrimidinase like 3
JAKMIP2	ENSSSCG00000014420	100511808	2	148,859,418	149,045,932	protein_coding	janus kinase and microtubule interacting protein 2
SPINK1	ENSSSCG00000034511	100622575	2	149,072,368	149,080,366	protein_coding	serine peptidase inhibitor, Kazal type 1
SCGB3A2	ENSSSCG00000024111	100622733	2	149,120,665	149,123,915	protein_coding	secretoglobin family 3A member 2
C5orf46	ENSSSCG00000034399	100622840	2	149,146,583	149,151,429	protein_coding	chromosome 5 open reading frame 46
SPINK5	ENSSSCG00000014422	100512160	2	149,311,084	149,375,733	protein_coding	serine peptidase inhibitor, Kazal type 5
SPINK14	ENSSSCG00000033173	100303616	2	149,414,655	149,423,768	protein_coding	serine peptidase inhibitor, Kazal type 14 (putative)
SPINK6	ENSSSCG00000039674	NA	2	149,470,402	149,486,649	protein_coding	serine peptidase inhibitor, Kazal type 6
SPINK7	ENSSSCG00000022035	NA	2	149,611,734	149,615,042	protein_coding	serine peptidase inhibitor, Kazal type 7 (putative)
SPINK9	ENSSSCG00000014426	100513074	2	149,630,230	149,634,973	protein_coding	serine peptidase inhibitor, Kazal type 9
FBXO38	ENSSSCG00000014427	100513273	2	149,674,292	149,722,865	protein_coding	F-box protein 38
ADRB2	ENSSSCG00000038598	397357	2	150,033,550	150,035,599	protein_coding	adrenoceptor beta 2
SH3TC2	ENSSSCG00000023279	100624660	2	150,184,182	150,248,174	protein_coding	SH3 domain and tetratricopeptide repeats 2
ABLIM3	ENSSSCG00000014430	100515194	2	150,260,183	150,423,211	protein_coding	actin binding LIM protein family member 3
GRPEL2	ENSSSCG00000014432	100513650	2	150,511,112	150,518,895	protein_coding	GrpE like 2, mitochondrial
PCYOX1L	ENSSSCG00000014433	100513977	2	150,526,229	150,537,242	protein_coding	prenylcysteine oxidase 1 like
IL17B	ENSSSCG00000014434	102162772	2	150,541,317	150,545,367	protein_coding	interleukin 17B
CSNK1A1	ENSSSCG00000014435	100514516	2	150,612,045	150,662,431	protein_coding	casein kinase 1 alpha 1
ARHGEF37	ENSSSCG00000014436	100514827	2	150,678,941	150,738,081	protein_coding	Rho guanine nucleotide exchange factor 37
PPARGC1B	ENSSSCG00000014437	100329221	2	150,823,368	150,919,366	protein_coding	PPARG coactivator 1 beta
PDE6A	ENSSSCG00000014438	100515715	2	150,927,770	150,985,318	protein_coding	phosphodiesterase 6A
SLC26A2	ENSSSCG00000039045	100515885	2	150,995,695	151,027,479	protein_coding	solute carrier family 26 member 2
HMGXB3	ENSSSCG00000014440	100516069	2	151,036,180	151,103,130	protein_coding	HMG-box containing 3
CSF1R	ENSSSCG00000014441	100517086	2	151,099,084	151,129,407	protein_coding	colony stimulating factor 1 receptor
PDGFRB	ENSSSCG00000022741	102166564	2	151,155,754	151,192,816	protein_coding	platelet derived growth factor receptor beta
CDX1	ENSSSCG00000014445	100626275	2	151,202,918	151,216,988	protein_coding	caudal type homeobox 1
SLC6A7	ENSSSCG00000014447	100516251	2	151,223,225	151,240,956	protein_coding	solute carrier family 6 member 7
CAMK2A	ENSSSCG00000014443	100626014	2	151,248,358	151,308,700	protein_coding	calcium/calmodulin dependent protein kinase II alpha
ARSI	ENSSSCG00000014448	NA	2	151,315,508	151,320,814	protein_coding	arylsulfatase family member I
TCOF1	ENSSSCG00000014450	100516425	2	151,368,377	151,402,253	protein_coding	treacle ribosome biogenesis factor 1

CD74	ENSSSCG00000033262	396660	2	151,399,969	151,433,844	protein_coding	CD74 molecule
RPS14	ENSSSCG00000031370	110259542	2	151,430,039	151,433,856	protein_coding	ribosomal protein S14
NDST1	ENSSSCG00000032731	110259541	2	151,463,230	151,522,773	protein_coding	N-deacetylase and N-sulfotransferase 1
PARN	ENSSSCG00000007878	100515887	3	28,616,808	28,802,505	protein_coding	poly(A)-specific ribonuclease
MIR193B	ENSSSCG00000034053	NA	3	28,913,091	28,913,173	miRNA	microRNA 193b
MRTFB	ENSSSCG00000039171	100626101	3	28,964,754	29,239,741	protein_coding	myocardin related transcription factor B
MRTFB	ENSSSCG00000039171	100626101	3	28,964,754	29,239,741	protein_coding	myocardin related transcription factor B
ERCC4	ENSSSCG00000007881	100516427	3	29,269,989	29,306,109	protein_coding	ERCC excision repair 4, endonuclease catalytic subunit
SHISA9	ENSSSCG00000032878	106509691	3	29,969,418	30,274,214	protein_coding	shisa family member 9
SNX29	ENSSSCG00000037232	100626399	3	30,584,713	31,133,305	protein_coding	sorting nexin 29
TNFRSF17	ENSSSCG00000032578	100517087	3	31,133,299	31,141,840	protein_coding	TNF receptor superfamily member 17
GSPT1	ENSSSCG00000021118	100517817	3	31,237,106	31,273,865	protein_coding	G1 to S phase transition 1
RSL1D1	ENSSSCG00000023984	102166714	3	31,278,389	31,291,204	protein_coding	ribosomal L1 domain containing 1
ZC3H7A	ENSSSCG00000007895	100517644	3	31,335,347	31,376,346	protein_coding	zinc finger CCCH-type containing 7A
TXNDC11	ENSSSCG00000007896	100622177	3	31,376,510	31,445,632	protein_coding	thioredoxin domain containing 11
SNN	ENSSSCG00000007897	100518118	3	31,448,340	31,448,603	protein_coding	stannin
LITAF	ENSSSCG00000032381	100518302	3	31,523,434	31,579,082	protein_coding	lipopolysaccharide induced TNF factor
RMI2	ENSSSCG00000022149	100518832	3	31,808,015	31,814,867	protein_coding	RecQ mediated genome instability 2
PRM3	ENSSSCG00000035607	100627438	3	31,866,340	31,866,618	protein_coding	protamine 3
TNP2	ENSSSCG00000027300	406180	3	31,869,720	31,871,564	protein_coding	transition protein 2
SOCS1	ENSSSCG00000027855	100307052	3	31,881,891	31,882,722	protein_coding	suppressor of cytokine signaling 1
CLEC16A	ENSSSCG00000026569	100519828	3	31,938,135	32,158,327	protein_coding	C-type lectin domain containing 16A
DEXI	ENSSSCG00000037224	100519361	3	32,160,742	32,161,029	protein_coding	Dexi homolog
CIITA	ENSSSCG00000007901	106509697	3	32,172,214	32,230,780	protein_coding	class II major histocompatibility complex transactivator
TVP23A	ENSSSCG00000027101	100620244	3	32,275,538	32,314,749	protein_coding	trans-golgi network vesicle protein 23 homolog A
NUBP1	ENSSSCG00000024488	100628026	3	32,311,783	32,331,987	protein_coding	nucleotide binding protein 1
TEKT5	ENSSSCG00000007904	100620428	3	32,350,906	32,392,952	protein_coding	tektin 5
EMP2	ENSSSCG00000037065	100739825	3	32,450,510	32,493,247	protein_coding	epithelial membrane protein 2
ATF7IP2	ENSSSCG00000020675	100520369	3	32,523,055	32,599,255	protein_coding	activating transcription factor 7 interacting protein 2
SLC8A1	ENSSSCG00000036418	100622020	3	99,823,548	100,244,770	protein_coding	solute carrier family 8 member A1
THUMPD2	ENSSSCG00000008470	100517336	3	100,556,833	100,600,909	protein_coding	THUMP domain containing 2
TMEM178A	ENSSSCG00000024293	100738444	3	100,613,881	100,669,651	protein_coding	transmembrane protein 178A
MAP4K3	ENSSSCG00000008475	100622259	3	100,886,834	101,084,677	protein_coding	mitogen-activated protein kinase kinase kinase kinase 3
SOS1	ENSSSCG00000008478	100738632	3	101,089,243	101,362,724	protein_coding	SOS Ras/Rac guanine nucleotide exchange factor 1
ARHGEF33	ENSSSCG00000040621	100739092	3	101,375,322	101,414,450	protein_coding	Rho guanine nucleotide exchange factor 33
MORN2	ENSSSCG00000008480	100519139	3	101,450,344	101,458,986	protein_coding	MORN repeat containing 2
DHX57	ENSSSCG00000008479	100520373	3	101,458,895	101,569,798	protein_coding	DExH-box helicase 57
GEMIN6	ENSSSCG00000008482	100518833	3	101,565,678	101,571,913	protein_coding	gem nuclear organelle associated protein 6
HNRNPLL	ENSSSCG00000020878	100622737	3	101,753,285	101,802,062	protein_coding	heterogeneous nuclear ribonucleoprotein L like
ATL2	ENSSSCG00000008487	100521264	3	101,956,722	102,029,667	protein_coding	atlastin GTPase 2
CYP1B1	ENSSSCG00000033844	100625479	3	102,195,741	102,206,375	protein_coding	cytochrome P450 family 1 subfamily B member 1
BIRC6	ENSSSCG00000008513	100049694	3	107,013,931	107,269,370	protein_coding	baculoviral IAP repeat containing 6
YIPF4	ENSSSCG00000035747	110260040	3	107,306,691	107,325,748	protein_coding	Yip1 domain family member 4
NLRC4	ENSSSCG00000026213	NA	3	107,367,117	107,387,264	protein_coding	NLR family CARD domain containing 4
SLC30A6	ENSSSCG00000008518	100170144	3	107,387,859	107,426,744	protein_coding	solute carrier family 30 member 6
SPAST	ENSSSCG00000008517	396584	3	107,442,593	107,508,619	protein_coding	spastin
DPY30	ENSSSCG00000008516	100514043	3	107,520,642	107,533,502	protein_coding	dpy-30, histone methyltransferase complex regulatory subunit
MEMO1	ENSSSCG00000008515	100513790	3	107,542,131	107,659,006	protein_coding	mediator of cell motility 1
SRD5A2	ENSSSCG00000008521	397048	3	107,840,200	107,918,350	protein_coding	steroid 5 alpha-reductase 2
XDH	ENSSSCG00000008522	100515259	3	107,986,528	108,053,169	protein_coding	xanthine dehydrogenase
EHD3	ENSSSCG00000035987	100621933	3	108,100,401	108,135,166	protein_coding	EH domain containing 3
CAPN14	ENSSSCG00000024781	NA	3	108,151,519	108,174,351	protein_coding	calpain 14
GALNT14	ENSSSCG00000008524	100738063	3	108,223,705	108,439,600	protein_coding	polypeptide N-acetylgalactosaminyltransferase 14
CAPN13	ENSSSCG00000020986	100515606	3	108,531,118	108,620,616	protein_coding	calpain 13
LBH	ENSSSCG00000040332	102160087	3	109,078,282	109,105,526	protein_coding	limb bud and heart development
YPEL5	ENSSSCG00000008529	100620823	3	109,178,094	109,196,645	protein_coding	yippee like 5
ALK	ENSSSCG00000008533	100518423	3	109,922,957	110,102,823	protein_coding	ALK receptor tyrosine kinase
CLIP4	ENSSSCG00000008535	100518607	3	110,111,153	110,181,161	protein_coding	CAP-Gly domain containing linker protein family member 4
PCARE	ENSSSCG00000024725	100622850	3	110,209,535	110,218,889	protein_coding	photoreceptor cilium actin regulator
TOGARAM2	ENSSSCG00000008534	100518963	3	110,231,915	110,276,917	protein_coding	TOG array regulator of axonemal microtubules 2
WDR43	ENSSSCG00000008536	100519832	3	110,316,873	110,365,092	protein_coding	WD repeat domain 43
TRMT61B	ENSSSCG00000030147	100520017	3	110,383,634	110,397,973	protein_coding	tRNA methyltransferase 61B
SPDYA	ENSSSCG00000008538	574061	3	110,395,651	110,433,373	protein_coding	speedy/RINGO cell cycle regulator family member A
PPP1CB	ENSSSCG00000008540	397378	3	110,436,526	110,474,765	protein_coding	protein phosphatase 1 catalytic subunit beta
PLB1	ENSSSCG00000034756	100519306	3	110,605,923	110,741,130	protein_coding	phospholipase B1
FOSL2	ENSSSCG00000032527	100623777	3	110,819,947	110,842,850	protein_coding	FOS like 2, AP-1 transcription factor subunit
BABAM2	ENSSSCG00000026465	100623966	3	110,896,187	111,328,397	protein_coding	BRISC and BRCA1 A complex member 2
RBKS	ENSSSCG00000038001	102163141	3	111,327,821	111,428,737	protein_coding	ribokinase
SLC4A1AP	ENSSSCG00000008549	100521846	3	111,508,424	111,531,608	protein_coding	solute carrier family 4 member 1 adaptor protein
SUPT7L	ENSSSCG00000008548	100521661	3	111,531,665	111,542,000	protein_coding	SPT7 like, STAGA complex gamma subunit
GPN1	ENSSSCG00000008546	100521316	3	111,537,605	111,565,696	protein_coding	GPN-loop GTPase 1
ZNF512	ENSSSCG00000008545	100521129	3	111,572,424	111,605,885	protein_coding	zinc finger protein 512
C2orf16	ENSSSCG00000008544	102158482	3	111,606,121	111,612,082	protein_coding	chromosome 2 open reading frame 16
GCKR	ENSSSCG00000028672	100625192	3	111,646,705	111,683,660	protein_coding	glucokinase regulator
FNDC4	ENSSSCG00000029305	100625689	3	111,678,760	111,683,660	protein_coding	fibronectin type III domain containing 4
IFT172	ENSSSCG00000026367	100625100	3	111,683,822	111,718,451	protein_coding	intraflagellar transport 172
KRTCAP3	ENSSSCG00000040062	102159544	3	111,715,005	111,720,154	protein_coding	keratinocyte associated protein 3
NRBP1	ENSSSCG00000022454	100625594	3	111,718,583	111,733,374	protein_coding	nuclear receptor binding protein 1
PPM1G	ENSSSCG00000027135	100739632	3	111,747,972	111,774,922	protein_coding	protein phosphatase, Mg2+/Mn2+ dependent 1G
ZNF513	ENSSSCG00000038626	100739726	3	111,771,239	111,778,885	protein_coding	zinc finger protein 513
SNX17	ENSSSCG00000025363	100739661	3	111,775,426	111,781,361	protein_coding	sorting nexin 17
EIF2B4	ENSSSCG00000023140	NA	3	111,782,068	111,786,329	protein_coding	eukaryotic translation initiation factor 2B subunit delta
GTF3C2	ENSSSCG00000032798	110260043	3	111,793,624	111,820,574	protein_coding	general transcription factor IIIC subunit 2
MPV17	ENSSSCG00000037201	110260044	3	111,823,089	111,833,927	protein_coding	mitochondrial inner membrane protein MPV17
UCN	ENSSSCG00000039206	110260045	3	111,834,181	111,835,997	protein_coding	urocortin
CAD	ENSSSCG00000028978	100624530	3	111,887,294	111,914,302	protein_coding	carbamoyl-phosphate synthetase 2, aspartate transcarbamylase, and dihydroorotase
SLC5A6	ENSSSCG00000008550	100522014	3	111,919,312	111,976,091	protein_coding	solute carrier family 5 member 6

TCF23	ENSSSCG00000027409	100522370	3	111,975,860	111,983,920	protein_coding	transcription factor 23
PRR30	ENSSSCG00000008552	100522547	3	111,989,375	111,990,568	protein_coding	proline rich 30
ABHD1	ENSSSCG00000008554	100525041	3	111,991,257	112,002,148	protein_coding	abhydrolase domain containing 1
PREB	ENSSSCG00000008553	100522734	3	111,992,031	111,999,913	protein_coding	prolactin regulatory element binding
CGREF1	ENSSSCG00000008555	100522927	3	112,002,144	112,020,208	protein_coding	cell growth regulator with EF-hand domain 1
KHK	ENSSSCG00000008556	100523241	3	112,021,165	112,031,620	protein_coding	ketohexokinase
EMILIN1	ENSSSCG00000008557	100523665	3	112,031,945	112,039,933	protein_coding	elastin microfibril interfacer 1
AGBL5	ENSSSCG00000008558	100523841	3	112,045,310	112,066,977	protein_coding	ATP/GTP binding protein like 5
TMEM214	ENSSSCG00000008559	100524558	3	112,069,384	112,080,653	protein_coding	transmembrane protein 214
MAPRE3	ENSSSCG00000029185	100524857	3	112,083,268	112,136,054	protein_coding	microtubule associated protein RP/EB family member 3
DPYSL5	ENSSSCG00000008560	100511810	3	112,155,542	112,252,968	protein_coding	dihydropyrimidinase like 5
RWDD3	ENSSSCG00000036509	100625281	4	122,184,310	122,203,100	protein_coding	RWD domain containing 3
ALG14	ENSSSCG00000006885	100157203	4	122,343,312	122,460,755	protein_coding	ALG14, UDP-N-acetylglucosaminyltransferase subunit
SLC44A3	ENSSSCG00000006888	100525692	4	122,529,983	122,621,379	protein_coding	solute carrier family 44 member 3
F3	ENSSSCG00000022447	396677	4	122,826,644	122,840,508	protein_coding	coagulation factor III, tissue factor
ABCD3	ENSSSCG00000028620	100626343	4	122,837,525	122,953,257	protein_coding	ATP binding cassette subfamily D member 3
ARHGAP29	ENSSSCG00000006889	100152734	4	123,073,602	123,158,548	protein_coding	Rho GTPase activating protein 29
ABCA4	ENSSSCG00000006890	100155583	4	123,202,753	123,331,144	protein_coding	ATP binding cassette subfamily A member 4
GCLM	ENSSSCG00000037549	100153977	4	123,371,193	123,390,129	protein_coding	glutamate-cysteine ligase modifier subunit
MIR760	ENSSSCG00000018816	NA	4	123,419,778	123,419,890	miRNA	microRNA 760
BCAR3	ENSSSCG00000006893	100153143	4	123,440,571	123,666,600	protein_coding	BCAR3, NSP family adaptor protein
FNBP1L	ENSSSCG00000006894	100157977	4	123,674,697	123,779,615	protein_coding	formin binding protein 1 like
DR1	ENSSSCG00000028310	100152776	4	123,813,838	123,835,840	protein_coding	down-regulator of transcription 1
CCDC18	ENSSSCG00000006897	100518781	4	123,902,320	124,008,748	protein_coding	coiled-coil domain containing 18
MTF2	ENSSSCG00000006898	100156808	4	124,048,806	124,124,440	protein_coding	metal response element binding transcription factor 2
DIPK1A	ENSSSCG00000006900	100157200	4	124,229,560	124,360,027	protein_coding	divergent protein kinase domain 1A
EVI5	ENSSSCG00000006901	100519143	4	124,367,736	124,600,581	protein_coding	ecotropic viral integration site 5
GFI1	ENSSSCG00000006902	110260381	4	124,622,419	124,629,180	protein_coding	growth factor independent 1 transcriptional repressor
RPAP2	ENSSSCG00000006903	100517893	4	124,691,657	124,773,890	protein_coding	RNA polymerase II associated protein 2
GLMN	ENSSSCG00000006904	100153954	4	124,773,964	124,813,323	protein_coding	glomulin, FKBP associated protein
BTBD8	ENSSSCG00000035196	100624686	4	124,813,397	124,950,422	protein_coding	BTB domain containing 8
TGFBR3	ENSSSCG00000006911	397512	4	125,109,966	125,310,783	protein_coding	transforming growth factor beta receptor 3
CDC7	ENSSSCG00000027251	100520671	4	125,429,530	125,459,489	protein_coding	cell division cycle 7
HFM1	ENSSSCG00000006912	100156806	4	125,522,334	125,606,882	protein_coding	HFM1, ATP dependent DNA helicase homolog
ZNF644	ENSSSCG00000022534	100152774	4	125,740,329	125,857,196	protein_coding	zinc finger protein 644
BARHL2	ENSSSCG00000006914	100521190	4	126,030,347	126,036,287	protein_coding	BarH like homeobox 2
ZNF326	ENSSSCG00000038253	100737600	4	126,677,956	126,707,220	protein_coding	zinc finger protein 326
LRRC8D	ENSSSCG00000038417	100737439	4	126,766,227	126,874,257	protein_coding	leucine rich repeat containing 8 VRAC subunit D
LRRC8C	ENSSSCG00000006917	100156403	4	126,957,103	127,079,209	protein_coding	leucine rich repeat containing 8 VRAC subunit C
LRRC8B	ENSSSCG00000037101	100155627	4	127,071,068	127,081,071	protein_coding	leucine rich repeat containing 8 VRAC subunit B
KYAT3	ENSSSCG00000006925	100157176	4	127,451,507	127,508,648	protein_coding	kynurenine aminotransferase 3
GTF2B	ENSSSCG00000006926	100155974	4	127,522,819	127,559,597	protein_coding	general transcription factor IIB
PKN2	ENSSSCG00000006927	100154766	4	127,573,740	127,709,942	protein_coding	protein kinase N2
LMO4	ENSSSCG00000006928	100127155	4	128,894,134	128,910,546	protein_coding	LIM domain only 4
SH3GLB1	ENSSSCG00000006930	100316852	4	129,357,552	129,395,576	protein_coding	SH3 domain containing GRB2 like, endophilin B1
ODF2L	ENSSSCG00000006936	100513149	4	129,476,422	129,804,203	protein_coding	outer dense fiber of sperm tails 2 like
CLCA2	ENSSSCG00000006935	100511753	4	129,668,254	129,705,211	protein_coding	chloride channel accessory 2
ZCCHC14	ENSSSCG00000002656	100512105	6	1,849,979	1,918,693	protein_coding	zinc finger CCHC-type containing 14
FBXO31	ENSSSCG00000002654	100512607	6	1,949,254	1,995,464	protein_coding	F-box protein 31
FOXL1	ENSSSCG00000002658	100511634	6	2,542,675	2,543,691	protein_coding	forkhead box L1
FOXC2	ENSSSCG00000029296	100526107	6	2,552,862	2,554,367	protein_coding	forkhead box C2
MTHFSD	ENSSSCG00000002660	100511815	6	2,561,212	2,586,607	protein_coding	methenyltetrahydrofolate synthetase domain containing
FOXF1	ENSSSCG00000038460	106510450	6	2,591,649	2,597,659	protein_coding	forkhead box F1
IRF8	ENSSSCG00000034980	396645	6	3,041,417	3,060,685	protein_coding	interferon regulatory factor 8
COX4I1	ENSSSCG00000029034	100624067	6	3,131,019	3,137,482	protein_coding	cytochrome c oxidase subunit 4I1
EMC8	ENSSSCG00000029403	100737206	6	3,136,311	3,154,641	protein_coding	ER membrane protein complex subunit 8
C16orf74	ENSSSCG00000002662	NA	6	3,205,946	3,210,734	protein_coding	chromosome 16 open reading frame 74
GINS2	ENSSSCG00000040386	100512784	6	3,218,662	3,250,361	protein_coding	GINS complex subunit 2
GSE1	ENSSSCG00000002664	100513153	6	3,246,730	3,349,122	protein_coding	Gse1 coiled-coil protein
FAM92B	ENSSSCG00000002668	100514288	6	3,710,166	3,721,279	protein_coding	family with sequence similarity 92 member B
KIAA0513	ENSSSCG00000002666	100515140	6	3,724,793	3,774,979	protein_coding	KIAA0513
ZDHHC7	ENSSSCG00000002667	100514104	6	3,786,338	3,817,952	protein_coding	zinc finger DHHC-type containing 7
CRISPLD2	ENSSSCG00000002669	100625354	6	3,880,718	3,944,680	protein_coding	cysteine rich secretory protein LCCL domain containing 2
USP10	ENSSSCG00000002670	100514462	6	3,977,231	4,043,533	protein_coding	ubiquitin specific peptidase 10
KLHL36	ENSSSCG00000038285	110260898	6	4,067,395	4,077,081	protein_coding	kelch like family member 36
COTL1	ENSSSCG00000037645	100621773	6	4,099,336	4,147,469	protein_coding	coactosin like F-actin binding protein 1
MEAK7	ENSSSCG00000002672	100514962	6	4,204,084	4,226,849	protein_coding	MTOR associated protein, eak-7 homolog
ATP2C2	ENSSSCG00000002671	100514770	6	4,240,668	4,313,279	protein_coding	ATPase secretory pathway Ca2+ transporting 2
WFDC1	ENSSSCG00000023837	100514648	6	4,333,730	4,365,650	protein_coding	WAP four-disulfide core domain 1
KCNG4	ENSSSCG00000030446	100516015	6	4,411,865	4,426,619	protein_coding	potassium voltage-gated channel modifier subfamily G member 4
ADAD2	ENSSSCG00000034385	100737909	6	4,437,941	4,445,546	protein_coding	adenosine deaminase domain containing 2
TAF1C	ENSSSCG00000002680	100516196	6	4,451,210	4,457,089	protein_coding	TATA-box binding protein associated factor, RNA polymerase I subunit C
DNAAF1	ENSSSCG00000002675	100517588	6	4,457,291	4,479,828	protein_coding	dynein axonemal assembly factor 1
HSDL1	ENSSSCG00000002681	100516372	6	4,479,917	4,503,474	protein_coding	hydroxysteroid dehydrogenase like 1
MBTPS1	ENSSSCG00000002682	100516674	6	4,503,479	4,558,615	protein_coding	membrane bound transcription factor peptidase, site 1
SLC38A8	ENSSSCG00000039218	110260902	6	4,565,105	4,593,591	protein_coding	solute carrier family 38 member 8
NECAB2	ENSSSCG00000038048	110260903	6	4,599,231	4,641,423	protein_coding	N-terminal EF-hand calcium binding protein 2
OSGIN1	ENSSSCG00000032115	110260904	6	4,641,956	4,653,497	protein_coding	oxidative stress induced growth inhibitor 1
CLEC3A	ENSSSCG00000034604	100519025	6	9,906,440	9,915,428	protein_coding	C-type lectin domain family 3 member A
VAT1L	ENSSSCG00000002696	100520197	6	9,957,503	10,120,510	protein_coding	vesicle amine transport 1 like
NUDT7	ENSSSCG00000002697	100738827	6	10,161,960	10,173,980	protein_coding	nudix hydrolase 7
ADAMTS18	ENSSSCG00000038627	102167408	6	10,488,573	10,650,626	protein_coding	ADAM metallopeptidase with thrombospondin type 1 motif 18
SYCE1L	ENSSSCG00000002700	NA	6	10,701,190	10,707,625	protein_coding	synaptonemal complex central element protein 1 like
MON1B	ENSSSCG00000002701	100520385	6	10,713,713	10,725,470	protein_coding	MON1 homolog B, secretory trafficking associated
CNTNAP4	ENSSSCG00000023125	100626866	6	11,317,846	11,589,630	protein_coding	contactin associated protein like 4
KARS	ENSSSCG00000002704	100521452	6	12,056,567	12,077,051	protein_coding	lysyl-tRNA synthetase
ADAT1	ENSSSCG00000002706	100521793	6	12,076,383	12,107,992	protein_coding	adenosine deaminase, tRNA specific 1

GABARAPL2	ENSSSCG00000002707	100521968	6	12,122,057	12,132,991	protein_coding	GABA type A receptor associated protein like 2
CFDP1	ENSSSCG00000033842	100144464	6	12,223,696	12,354,403	protein_coding	craniofacial development protein 1
BCAR1	ENSSSCG00000028878	100621541	6	12,369,733	12,404,809	protein_coding	BCAR1, Cas family scaffold protein
ZFP1	ENSSSCG00000022668	100622002	6	12,482,182	12,531,529	protein_coding	ZFP1 zinc finger protein
LDHD	ENSSSCG00000002712	100523064	6	12,548,131	12,555,648	protein_coding	lactate dehydrogenase D
ZNRF1	ENSSSCG00000031462	NA	6	12,555,659	12,668,810	protein_coding	zinc and ring finger 1
WDR59	ENSSSCG00000002714	100521092	6	12,682,184	12,771,481	protein_coding	WD repeat domain 59
FA2H	ENSSSCG00000002718	100523966	6	12,855,421	12,909,499	protein_coding	fatty acid 2-hydroxylase
MLKL	ENSSSCG00000002716	100736836	6	12,924,904	12,948,362	protein_coding	mixed lineage kinase domain like pseudokinase
RFWD3	ENSSSCG00000002715	100736590	6	12,952,241	12,994,052	protein_coding	ring finger and WD repeat domain 3
GLG1	ENSSSCG00000030420	100622584	6	13,011,888	13,160,379	protein_coding	golgi glycoprotein 1
PDPR	ENSSSCG00000002724	100525224	6	13,187,422	13,230,598	protein_coding	pyruvate dehydrogenase phosphatase regulatory subunit
EXOSC6	ENSSSCG00000002719	100737302	6	13,296,055	13,296,873	protein_coding	exosome component 6
DDX19B	ENSSSCG00000002722	100524746	6	13,328,202	13,389,884	protein_coding	DEAD-box helicase 19B
ST3GAL2	ENSSSCG00000038843	100627772	6	13,402,345	13,456,455	protein_coding	ST3 beta-galactoside alpha-2,3-sialyltransferase 2
FUK	ENSSSCG00000025425	100625448	6	13,471,833	13,493,749	protein_coding	fucokinase
SF3B3	ENSSSCG00000025323	100512659	6	13,524,716	13,578,845	protein_coding	splicing factor 3b subunit 3
IL34	ENSSSCG00000040629	100737468	6	13,588,787	13,662,753	protein_coding	interleukin 34
MTSS1L	ENSSSCG00000040037	100513016	6	13,659,346	13,686,106	protein_coding	MTSS1L, I-BAR domain containing
HYDIN	ENSSSCG00000040973	100513208	6	13,895,493	14,233,941	protein_coding	HYDIN, axonemal central pair apparatus protein
PSMD7	ENSSSCG00000032908	100626274	6	17,046,650	17,056,691	protein_coding	proteasome 26S subunit, non-ATPase 7
WWP2	ENSSSCG00000022609	100626361	6	17,068,783	17,228,800	protein_coding	WW domain containing E3 ubiquitin protein ligase 2
NOB1	ENSSSCG00000002753	100516270	6	17,233,846	17,247,471	protein_coding	NIN1 (RPN12) binding protein 1 homolog
NFAT5	ENSSSCG00000002755	100516737	6	17,313,413	17,434,951	protein_coding	nuclear factor of activated T cells 5
TERF2	ENSSSCG00000002762	100517762	6	17,605,871	17,634,604	protein_coding	telomeric repeat binding factor 2
SNTB2	ENSSSCG00000028109	100620497	6	17,681,896	17,774,496	protein_coding	syntrophin beta 2
UTP4	ENSSSCG00000028638	414432	6	17,782,199	17,818,173	protein_coding	UTP4, small subunit processome component
HAS3	ENSSSCG00000035601	408053	6	17,844,974	17,856,082	protein_coding	hyaluronan synthase 3
TANGO6	ENSSSCG00000031767	110260922	6	17,870,616	18,051,377	protein_coding	transport and golgi organization 6 homolog
CDH3	ENSSSCG00000023162	100187727	6	18,191,664	18,238,129	protein_coding	cadherin 3
ZFP90	ENSSSCG00000032372	100620865	6	18,312,936	18,335,962	protein_coding	ZFP90 zinc finger protein
BBS2	ENSSSCG00000025417	100622382	6	18,542,387	18,575,514	protein_coding	Bardet-Biedl syndrome 2
MT4	ENSSSCG00000031359	110260925	6	18,610,593	18,613,967	protein_coding	metallothionein 4
NUP93	ENSSSCG00000025830	100626040	6	18,707,989	18,827,890	protein_coding	nucleoporin 93
MIR138-2	ENSSSCG00000022561	NA	6	18,840,224	18,840,306	miRNA	microRNA 138-2
SLC12A3	ENSSSCG00000032643	110260927	6	18,844,799	18,885,179	protein_coding	solute carrier family 12 member 3
HERPUD1	ENSSSCG00000040663	100622579	6	18,877,492	18,916,601	protein_coding	homocysteine inducible ER protein with ubiquitin like domain 1
NLRC5	ENSSSCG00000039751	100135667	6	18,932,637	19,036,843	protein_coding	NLR family CARD domain containing 5
CPNE2	ENSSSCG00000021519	100627365	6	19,046,799	19,098,915	protein_coding	copine 2
FAM192A	ENSSSCG00000034414	106508675	6	19,098,096	19,154,210	protein_coding	family with sequence similarity 192 member A
RSPRY1	ENSSSCG00000002820	100515835	6	19,154,419	19,204,824	protein_coding	ring finger and SPRY domain containing 1
ARL2BP	ENSSSCG00000002819	100515548	6	19,207,008	19,215,025	protein_coding	ADP ribosylation factor like GTPase 2 binding protein
PLLP	ENSSSCG00000002818	100516614	6	19,211,772	19,241,256	protein_coding	plasmolipin
CX3CL1	ENSSSCG00000024759	100621027	6	19,312,447	19,324,088	protein_coding	C-X3-C motif chemokine ligand 1
CCL17	ENSSSCG00000023852	780408	6	19,345,764	19,348,965	protein_coding	C-C motif chemokine ligand 17
CIAPIN1	ENSSSCG00000029519	100620929	6	19,356,589	19,373,812	protein_coding	cytokine induced apoptosis inhibitor 1
COQ9	ENSSSCG00000025284	100620830	6	19,373,047	19,390,735	protein_coding	coenzyme Q9
POLR2C	ENSSSCG00000025454	100620737	6	19,393,564	19,402,212	protein_coding	RNA polymerase II subunit C
DOK4	ENSSSCG00000029165	100620637	6	19,401,070	19,413,641	protein_coding	docking protein 4
CCDC102A	ENSSSCG00000028723	100620539	6	19,437,399	19,457,130	protein_coding	coiled-coil domain containing 102A
ADGRG5	ENSSSCG00000025503	100621610	6	19,463,024	19,489,761	protein_coding	adhesion G protein-coupled receptor G5
ADGRG1	ENSSSCG00000002815	100515029	6	19,527,450	19,570,893	protein_coding	adhesion G protein-coupled receptor G1
ADGRG3	ENSSSCG00000002814	100514842	6	19,574,585	19,601,100	protein_coding	adhesion G protein-coupled receptor G3
DRC7	ENSSSCG00000002817	100515380	6	19,603,448	19,629,173	protein_coding	dynein regulatory complex subunit 7
KATNB1	ENSSSCG00000002813	100514649	6	19,630,350	19,661,010	protein_coding	katanin regulatory subunit B1
KIFC3	ENSSSCG00000002812	100514463	6	19,659,991	19,696,081	protein_coding	kinesin family member C3
ZNF319	ENSSSCG00000002808	102160889	6	19,845,193	19,851,325	protein_coding	zinc finger protein 319
MMP15	ENSSSCG00000002806	100514105	6	19,890,884	19,918,244	protein_coding	matrix metallopeptidase 15
CFAP20	ENSSSCG00000002805	100513209	6	19,948,918	19,969,347	protein_coding	cilia and flagella associated protein 20
CSNK2A2	ENSSSCG00000002804	100737256	6	19,974,666	20,016,754	protein_coding	casein kinase 2 alpha 2
CCDC113	ENSSSCG00000002803	100512660	6	20,052,735	20,088,815	protein_coding	coiled-coil domain containing 113
PRSS54	ENSSSCG00000028587	NA	6	20,085,891	20,099,726	protein_coding	serine protease 54
GINS3	ENSSSCG00000002802	100512472	6	20,162,537	20,171,485	protein_coding	GINS complex subunit 3
NDRG4	ENSSSCG00000021911	100513927	6	20,222,226	20,297,225	protein_coding	NDRG family member 4
SETD6	ENSSSCG00000026505	100626287	6	20,267,048	20,270,442	protein_coding	SET domain containing 6
CNOT1	ENSSSCG00000002799	100511993	6	20,271,594	20,372,920	protein_coding	CCR4-NOT transcription complex subunit 1
SLC38A7	ENSSSCG00000040881	100511635	6	20,387,792	20,401,701	protein_coding	solute carrier family 38 member 7
AGO3	ENSSSCG00000003630	100499509	6	91,967,494	92,212,455	protein_coding	argonaute 3, RISC catalytic component
TEKT2	ENSSSCG00000003633	100525228	6	92,233,586	92,237,900	protein_coding	tektin 2
ADPRHL2	ENSSSCG00000003634	100525405	6	92,238,473	92,244,514	protein_coding	ADP-ribosylhydrolase like 2
COL8A2	ENSSSCG00000033641	100525585	6	92,244,005	92,262,808	protein_coding	collagen type VIII alpha 2 chain
TRAPPC3	ENSSSCG00000037213	100525755	6	92,279,643	92,293,561	protein_coding	trafficking protein particle complex 3
MAP7D1	ENSSSCG00000003637	100511265	6	92,298,780	92,322,693	protein_coding	MAP7 domain containing 1
THRAP3	ENSSSCG00000029954	100627402	6	92,350,072	92,414,023	protein_coding	thyroid hormone receptor associated protein 3
STK40	ENSSSCG00000036832	100625215	6	92,410,292	92,484,336	protein_coding	serine/threonine kinase 40
SH3D21	ENSSSCG00000028892	100625427	6	92,412,690	92,429,696	protein_coding	SH3 domain containing 21
EVA1B	ENSSSCG00000033892	100625314	6	92,430,205	92,432,137	protein_coding	eva-1 homolog B
OSCP1	ENSSSCG00000036709	100622140	6	92,489,923	92,537,537	protein_coding	organic solute carrier partner 1
LSM10	ENSSSCG00000038916	110261147	6	92,489,927	92,493,665	protein_coding	LSM10, U7 small nuclear RNA associated
MRPS15	ENSSSCG00000034647	100622235	6	92,537,599	92,547,750	protein_coding	mitochondrial ribosomal protein S15
CSF3R	ENSSSCG00000025795	100622336	6	92,550,075	92,562,431	protein_coding	colony stimulating factor 3 receptor
GRIK3	ENSSSCG00000003640	100511641	6	92,886,610	93,128,582	protein_coding	glutamate ionotropic receptor kainate type subunit 3
ZC3H12A	ENSSSCG00000037815	110261148	6	93,541,334	93,551,193	protein_coding	zinc finger CCCH-type containing 12A
MEAF6	ENSSSCG00000034611	100627675	6	93,558,899	93,589,066	protein_coding	MYST/Esa1 associated factor 6
SNIP1	ENSSSCG00000029991	100620923	6	93,597,059	93,612,150	protein_coding	Smad nuclear interacting protein 1
DNALI1	ENSSSCG00000039034	100623248	6	93,612,197	93,622,081	protein_coding	dynein axonemal light intermediate chain 1
GNL2	ENSSSCG00000031631	100623150	6	93,616,575	93,646,019	protein_coding	G protein nucleolar 2
RSPO1	ENSSSCG00000032240	100622113	6	93,665,749	93,690,660	protein_coding	R-spondin 1

C1orf109	ENSSSCG00000039905	110261150	6	93,741,012	93,750,598	protein_coding	chromosome 1 open reading frame 109
CDCA8	ENSSSCG00000032909	110261149	6	93,750,662	93,765,483	protein_coding	cell division cycle associated 8
EPHA10	ENSSSCG00000038675	110261151	6	93,767,723	93,803,855	protein_coding	EPH receptor A10
MANEAL	ENSSSCG00000032960	110261152	6	93,821,734	93,829,456	protein_coding	mannosidase endo-alpha like
YRDC	ENSSSCG00000040425	110261153	6	93,829,321	93,833,643	protein_coding	yrdC N6-threonylcarbamoyltransferase domain containing
C1orf122	ENSSSCG00000038888	110261154	6	93,833,760	93,834,916	protein_coding	chromosome 1 open reading frame 122
MTF1	ENSSSCG00000037516	100511998	6	93,839,200	93,884,417	protein_coding	metal regulatory transcription factor 1
INPP5B	ENSSSCG00000003642	100512110	6	93,885,459	93,944,384	protein_coding	inositol polyphosphate-5-phosphatase B
SF3A3	ENSSSCG00000003643	100512288	6	93,948,335	93,976,158	protein_coding	splicing factor 3a subunit 3
FHL3	ENSSSCG00000003644	396986	6	93,980,635	93,988,573	protein_coding	four and a half LIM domains 3
POU3F1	ENSSSCG00000003646	397513	6	94,026,675	94,028,030	protein_coding	POU class 3 homeobox 1
RRAGC	ENSSSCG00000003648	100513990	6	94,785,910	94,814,537	protein_coding	Ras related GTP binding C
GJA9	ENSSSCG00000003650	110261155	6	94,815,600	94,817,147	protein_coding	gap junction protein alpha 9
RHBDL2	ENSSSCG00000003651	100514296	6	94,821,350	94,864,101	protein_coding	rhomboid like 2
MACF1	ENSSSCG00000003654	106504077	6	95,150,836	95,304,767	protein_coding	microtubule-actin crosslinking factor 1
HEYL	ENSSSCG00000038956	100518256	6	95,445,150	95,453,073	protein_coding	hes related family bHLH transcription factor with YRPW motif-like
NT5C1A	ENSSSCG00000003662	100516203	6	95,480,716	95,496,905	protein_coding	5'-nucleotidase, cytosolic IA
HPCAL4	ENSSSCG00000023460	100144585	6	95,502,684	95,515,871	protein_coding	hippocalcin like 4
PPIE	ENSSSCG00000003664	100516562	6	95,548,663	95,583,408	protein_coding	peptidylprolyl isomerase E
MYCL	ENSSSCG00000039761	100518432	6	95,696,089	95,702,648	protein_coding	MYCL proto-oncogene, bHLH transcription factor
MFSD2A	ENSSSCG00000003669	100518612	6	95,738,805	95,756,002	protein_coding	major facilitator superfamily domain containing 2A
CAP1	ENSSSCG00000003672	100621359	6	95,811,553	95,840,732	protein_coding	cyclase associated actin cytoskeleton regulatory protein 1
PPT1	ENSSSCG00000036673	100517533	6	95,839,159	95,861,692	protein_coding	palmitoyl-protein thioesterase 1
RLF	ENSSSCG00000003670	100620967	6	95,900,086	95,994,043	protein_coding	rearranged L-myc fusion
TMCO2	ENSSSCG00000036395	100621068	6	96,000,864	96,003,985	protein_coding	transmembrane and coiled-coil domains 2
ZMPSTE24	ENSSSCG00000027902	106507628	6	96,008,304	96,057,123	protein_coding	zinc metallopeptidase STE24
MC2R	ENSSSCG00000039227	100739231	6	96,265,764	96,266,657	protein_coding	melanocortin 2 receptor
MC5R	ENSSSCG00000028411	NA	6	96,322,422	96,323,387	protein_coding	melanocortin 5 receptor
RNMT	ENSSSCG00000025855	100621823	6	96,342,914	96,360,169	protein_coding	RNA guanine-7 methyltransferase
FAM210A	ENSSSCG00000030438	100622095	6	96,384,381	96,393,999	protein_coding	family with sequence similarity 210 member A
LDLRAD4	ENSSSCG00000027417	100622189	6	96,401,652	96,577,621	protein_coding	low density lipoprotein receptor class A domain containing 4
TRIM39	ENSSSCG00000001342	100151742	7	23,050,195	23,064,498	protein_coding	tripartite motif containing 39
RPP21	ENSSSCG00000021130	100152493	7	23,064,366	23,066,287	protein_coding	ribonuclease P/MRP subunit p21
PRR3	ENSSSCG00000025892	NA	7	23,113,563	23,118,907	protein_coding	proline rich 3
ABCF1	ENSSSCG00000001346	100144452	7	23,126,307	23,143,994	protein_coding	ATP binding cassette subfamily F member 1
MIR877	ENSSSCG00000025435	NA	7	23,136,783	23,136,882	miRNA	microRNA 877
PPP1R10	ENSSSCG00000001347	100144450	7	23,149,933	23,165,456	protein_coding	protein phosphatase 1 regulatory subunit 10
ATAT1	ENSSSCG00000024604	100144480	7	23,173,461	23,185,458	protein_coding	alpha tubulin acetyltransferase 1
C6orf136	ENSSSCG00000025943	100153952	7	23,184,700	23,190,299	protein_coding	chromosome 6 open reading frame 136
DHX16	ENSSSCG00000001376	100144456	7	23,186,470	23,208,507	protein_coding	DEAH-box helicase 16
PPP1R18	ENSSSCG00000031492	100151743	7	23,209,172	23,221,917	protein_coding	protein phosphatase 1 regulatory subunit 18
NRM	ENSSSCG00000023639	100144454	7	23,222,312	23,225,519	protein_coding	nurim
MDC1	ENSSSCG00000001362	100144453	7	23,230,097	23,243,494	protein_coding	mediator of DNA damage checkpoint 1
FLOT1	ENSSSCG00000022725	100151746	7	23,249,124	23,265,109	protein_coding	flotillin 1
IER3	ENSSSCG00000027607	100154770	7	23,265,564	23,266,827	protein_coding	immediate early response 3
DDR1	ENSSSCG00000001382	100144462	7	23,373,583	23,394,965	protein_coding	discoidin domain receptor tyrosine kinase 1
GTF2H4	ENSSSCG00000001383	100144458	7	23,398,385	23,404,128	protein_coding	general transcription factor IIH subunit 4
VARS2	ENSSSCG00000001384	100144457	7	23,405,023	23,549,747	protein_coding	valyl-tRNA synthetase 2, mitochondrial
SFTA2	ENSSSCG00000037154	100520390	7	23,420,558	23,421,136	protein_coding	surfactant associated 2
C6orf15	ENSSSCG00000001388	100144463	7	23,515,531	23,517,110	protein_coding	chromosome 6 open reading frame 15
PSORS1C2	ENSSSCG00000034471	396560	7	23,540,744	23,541,913	protein_coding	psoriasis susceptibility 1 candidate 2
CCHCR1	ENSSSCG00000001391	100153563	7	23,544,129	23,558,270	protein_coding	coiled-coil alpha-helical rod protein 1
TCF19	ENSSSCG00000001392	100152381	7	23,558,128	23,562,074	protein_coding	transcription factor 19
DDX39B	ENSSSCG00000001400	448813	7	23,654,600	23,669,155	protein_coding	DExD-box helicase 39B
MCCD1	ENSSSCG00000023018	100157598	7	23,656,903	23,657,791	protein_coding	mitochondrial coiled-coil domain 1
NFKBIL1	ENSSSCG00000001402	100154350	7	23,668,778	23,685,087	protein_coding	NFKB inhibitor like 1
ATP6V1G2-DDX39	ENSSSCG00000001401	100152358	7	23,670,965	23,673,279	protein_coding	ATP6V1G2-DDX39B readthrough (NMD candidate)
ATP6V1G2	ENSSSCG00000001401	100152358	7	23,670,965	23,673,279	protein_coding	ATPase H+ transporting V1 subunit G2
LTB	ENSSSCG00000001405	100155581	7	23,687,548	23,706,738	protein_coding	lymphotoxin beta
AIF1	ENSSSCG00000032341	397271	7	23,729,215	23,734,949	protein_coding	allograft inflammatory factor 1
APOM	ENSSSCG00000001411	692188	7	23,770,896	23,774,428	protein_coding	apolipoprotein M
GPANK1	ENSSSCG00000001413	100156389	7	23,777,104	23,780,346	protein_coding	G-patch domain and ankyrin repeats 1
LY6G5C	ENSSSCG00000001416	100521803	7	23,791,553	23,795,734	protein_coding	lymphocyte antigen 6 family member G5C
ABHD16A	ENSSSCG00000034923	100155979	7	23,799,337	23,824,299	protein_coding	abhydrolase domain containing 16A
DDAH2	ENSSSCG00000038514	110261496	7	23,802,617	23,842,498	protein_coding	dimethylarginine dimethylaminohydrolase 2
LY6G6F	ENSSSCG00000033590	100271883	7	23,810,258	23,828,758	protein_coding	lymphocyte antigen 6 family member G6F
LY6G6E	ENSSSCG00000040885	110261493	7	23,822,741	23,825,399	protein_coding	lymphocyte antigen 6 family member G6E
LY6G6C	ENSSSCG00000036566	110261495	7	23,829,363	23,833,540	protein_coding	lymphocyte antigen 6 family member G6C
MPIG6B	ENSSSCG00000034664	110261494	7	23,829,506	23,838,637	protein_coding	megakaryocyte and platelet inhibitory receptor G6b
SAPCD1	ENSSSCG00000036076	NA	7	23,873,287	23,874,733	protein_coding	suppressor APC domain containing 1
HSPA1L	ENSSSCG00000030368	100144518	7	23,909,960	23,914,517	protein_coding	heat shock protein family A (Hsp70) member 1 like
SLC44A4	ENSSSCG00000001419	100124377	7	23,958,941	23,978,188	protein_coding	solute carrier family 44 member 4
ZBTB12	ENSSSCG00000001421	100124380	7	23,994,530	23,996,980	protein_coding	zinc finger and BTB domain containing 12
NELFE	ENSSSCG00000001423	100124384	7	24,040,368	24,046,983	protein_coding	negative elongation factor complex member E
SKIV2L	ENSSSCG00000001424	100124376	7	24,046,965	24,060,446	protein_coding	Ski2 like RNA helicase
DXO	ENSSSCG00000001425	100124385	7	24,057,670	24,060,446	protein_coding	decapping exoribonuclease
CYP21A2	ENSSSCG00000001428	403337	7	24,086,258	24,089,371	protein_coding	cytochrome P450 family 21 subfamily A member 2
ATF6B	ENSSSCG00000001430	100144516	7	24,161,053	24,171,742	protein_coding	activating transcription factor 6 beta
PPT2	ENSSSCG00000001433	100144537	7	24,188,608	24,202,622	protein_coding	palmitoyl-protein thioesterase 2
EGFL8	ENSSSCG00000001434	100144521	7	24,201,651	24,204,978	protein_coding	EGF like domain multiple 8
AGER	ENSSSCG00000001437	396591	7	24,217,772	24,221,082	protein_coding	advanced glycosylation end-product specific receptor
GPSM3	ENSSSCG00000001439	100144533	7	24,227,573	24,229,776	protein_coding	G protein signaling modulator 3
BTNL2	ENSSSCG00000025071	100158014	7	24,786,953	24,810,227	protein_coding	butyrophilin like 2
HLA-DRA	ENSSSCG00000001453	100135040	7	24,825,011	24,830,844	protein_coding	major histocompatibility complex, class II, DR alpha
HLA-DOB	ENSSSCG00000001459	100135049	7	24,984,834	25,037,892	protein_coding	major histocompatibility complex, class II, DO beta
TAP1	ENSSSCG00000025618	733649	7	25,062,771	25,072,038	protein_coding	transporter 1, ATP binding cassette subfamily B member
PSMB8	ENSSSCG00000001463	654294	7	25,071,712	25,077,233	protein_coding	proteasome subunit beta 9
BRD2	ENSSSCG00000001471	100141307	7	25,150,658	25,165,433	protein_coding	bromodomain containing 2

COL11A2	ENSSSCG00000001473	100520915	7	25,203,600	25,234,855	protein_coding	collagen type XI alpha 2 chain
RXRB	ENSSSCG00000001474	100156801	7	25,236,009	25,243,176	protein_coding	retinoid X receptor beta
SLC39A7	ENSSSCG00000001475	100155600	7	25,242,292	25,247,685	protein_coding	solute carrier family 39 member 7
HSD17B8	ENSSSCG00000001476	100154372	7	25,247,608	25,249,786	protein_coding	hydroxysteroid 17-beta dehydrogenase 8
HMGCLL1	ENSSSCG00000001479	100627473	7	25,596,716	25,783,612	protein_coding	3-hydroxymethyl-3-methylglutaryl-CoA lyase like 1
GFRAL	ENSSSCG00000001480	100521501	7	25,751,185	25,808,637	protein_coding	GDNF family receptor alpha like
FAM83B	ENSSSCG00000001483	100155972	7	26,058,144	26,141,831	protein_coding	family with sequence similarity 83 member B
TINAG	ENSSSCG00000001484	100153159	7	26,447,977	26,523,010	protein_coding	tubulointerstitial nephritis antigen
MLIP	ENSSSCG00000001485	100623023	7	26,561,359	26,793,726	protein_coding	muscular LMNA interacting protein
LRRC1	ENSSSCG00000001486	100154780	7	26,860,140	26,989,670	protein_coding	leucine rich repeat containing 1
KLHL31	ENSSSCG00000001487	100152767	7	27,062,044	27,106,117	protein_coding	kelch like family member 31
CEP128	ENSSSCG00000002410	100520033	7	103,353,895	103,760,323	protein_coding	centrosomal protein 128
TSHR	ENSSSCG00000031771	397560	7	103,768,260	103,926,287	protein_coding	thyroid stimulating hormone receptor
GTF2A1	ENSSSCG00000002411	100154605	7	103,963,245	104,009,302	protein_coding	general transcription factor IIA subunit 1
STON2	ENSSSCG00000002412	100157426	7	104,039,514	104,190,377	protein_coding	stonin 2
SEL1L	ENSSSCG00000002414	100520751	7	104,218,296	104,283,256	protein_coding	SEL1L, ERAD E3 ligase adaptor subunit
FOXN3	ENSSSCG00000002429	733584	7	111,036,504	111,453,936	protein_coding	forkhead box N3
EFCAB11	ENSSSCG00000002430	100738763	7	111,577,724	111,766,344	protein_coding	EF-hand calcium binding domain 11
TDP1	ENSSSCG00000002431	100153247	7	111,767,429	111,854,907	protein_coding	tyrosyl-DNA phosphodiesterase 1
KCNK13	ENSSSCG00000002432	100623906	7	111,866,983	111,980,485	protein_coding	potassium two pore domain channel subfamily K member 13
PSMC1	ENSSSCG00000002433	100155274	7	112,027,866	112,045,308	protein_coding	proteasome 26S subunit, ATPase 1
NRDE2	ENSSSCG00000002434	100154479	7	112,039,780	112,090,542	protein_coding	NRDE-2, necessary for RNA interference, domain containing
TTC7B	ENSSSCG00000002436	100154890	7	112,268,301	112,519,208	protein_coding	tetratricopeptide repeat domain 7B
RPS6KA5	ENSSSCG00000024377	100623751	7	112,561,988	112,753,617	protein_coding	ribosomal protein S6 kinase A5
GPR68	ENSSSCG00000034114	100153615	7	112,885,163	112,904,246	protein_coding	G protein-coupled receptor 68
PPP4R3A	ENSSSCG00000002441	100157672	7	113,111,303	113,148,986	protein_coding	protein phosphatase 4 regulatory subunit 3A
CATSPERB	ENSSSCG00000002442	100512175	7	113,197,964	113,293,597	protein_coding	cation channel sperm associated auxiliary subunit beta
TC2N	ENSSSCG00000002443	100156455	7	113,337,825	113,392,337	protein_coding	tandem C2 domains, nuclear
FBLN5	ENSSSCG00000002444	100155248	7	113,423,632	113,512,182	protein_coding	fibulin 5
TRIP11	ENSSSCG00000002445	100154452	7	113,525,678	113,600,581	protein_coding	thyroid hormone receptor interactor 11
ATXN3	ENSSSCG00000002446	100144422	7	113,608,781	113,645,844	protein_coding	ataxin 3
CPSF2	ENSSSCG00000002447	106507753	7	113,659,452	113,691,241	protein_coding	cleavage and polyadenylation specific factor 2
SLC24A4	ENSSSCG00000002450	100156914	7	113,836,230	114,014,949	protein_coding	solute carrier family 24 member 4
RIN3	ENSSSCG00000002451	100155709	7	114,035,383	114,541,592	protein_coding	Ras and Rab interactor 3
LGMN	ENSSSCG00000002452	100154477	7	114,175,183	114,217,319	protein_coding	legumain
GOLGA5	ENSSSCG00000002455	100151911	7	114,210,205	114,277,813	protein_coding	golgin A5
CHGA	ENSSSCG00000002456	397540	7	114,345,091	114,358,571	protein_coding	chromogranin 1
ITPK1	ENSSSCG00000002457	100152339	7	114,361,968	114,520,179	protein_coding	inositol-tetrakisphosphate 1-kinase
UBR7	ENSSSCG00000002460	100153874	7	114,590,945	114,612,481	protein_coding	ubiquitin protein ligase E3 component n-recognin 7 (putative)
BTBD7	ENSSSCG00000002461	100152664	7	114,619,396	114,707,051	protein_coding	BTB domain containing 7
COX8C	ENSSSCG00000002462	100157935	7	114,727,655	114,729,235	protein_coding	cytochrome c oxidase subunit 8C
PRIMA1	ENSSSCG00000002464	102167492	7	115,038,479	115,100,924	protein_coding	proline rich membrane anchor 1
FAM181A	ENSSSCG00000040253	100627495	7	115,193,032	115,207,054	protein_coding	family with sequence similarity 181 member A
ASB2	ENSSSCG00000002467	100153068	7	115,208,701	115,258,835	protein_coding	ankyrin repeat and SOCS box containing 2
CCDC197	ENSSSCG00000039415	NA	7	115,281,434	115,293,295	protein_coding	coiled-coil domain containing 197
OTUB2	ENSSSCG00000002469	100151873	7	115,304,815	115,326,047	protein_coding	OTU deubiquitinase, ubiquitin aldehyde binding 2
DDX24	ENSSSCG00000002470	100157901	7	115,328,119	115,348,745	protein_coding	DEAD-box helicase 24
IFI27L2	ENSSSCG00000002472	NA	7	115,367,850	115,369,991	protein_coding	interferon alpha inducible protein 27 like 2
PPP4R4	ENSSSCG00000002474	100737576	7	115,412,469	115,522,683	protein_coding	protein phosphatase 4 regulatory subunit 4
SERPINA6	ENSSSCG00000002475	396736	7	115,535,471	115,554,817	protein_coding	serpin family A member 6
SERPINA11	ENSSSCG00000002479	100155953	7	115,665,949	115,682,043	protein_coding	serpin family A member 11
SERPINA12	ENSSSCG00000002478	106504546	7	115,720,677	115,734,816	protein_coding	serpin family A member 12
SERPINA4	ENSSSCG00000039378	100157117	7	115,783,931	115,792,110	protein_coding	serpin family A member 4
SERPINA5	ENSSSCG00000002481	100153513	7	115,799,415	115,810,991	protein_coding	serpin family A member 5
GSC	ENSSSCG00000002490	397342	7	116,099,047	116,100,966	protein_coding	goosecoid homeobox
DICER1	ENSSSCG00000028225	100505387	7	116,361,678	116,436,396	protein_coding	dicer 1, ribonuclease III
SYNE3	ENSSSCG00000002495	100525411	7	116,665,429	116,770,312	protein_coding	spectrin repeat containing nuclear envelope family member 3
GLRX5	ENSSSCG00000002496	100154744	7	116,787,341	116,797,488	protein_coding	glutaredoxin 5
TCL1B	ENSSSCG00000002497	100525932	7	116,912,976	116,917,934	protein_coding	T cell leukemia/lymphoma 1B
TCL1A	ENSSSCG00000002498	100156364	7	116,934,941	116,939,261	protein_coding	T cell leukemia/lymphoma 1A
C14orf132	ENSSSCG00000033807	100154278	7	117,242,671	117,290,347	protein_coding	chromosome 14 open reading frame 132
LDB2	ENSSSCG00000029227	100513100	8	11,641,064	12,038,210	protein_coding	LIM domain binding 2
CLRN2	ENSSSCG00000023941	100739515	8	12,551,892	12,564,379	protein_coding	clarin 2
LAP3	ENSSSCG00000023604	100739583	8	12,590,330	12,619,107	protein_coding	leucine aminopeptidase 3
MED28	ENSSSCG00000029989	100627415	8	12,624,104	12,633,248	protein_coding	mediator complex subunit 28
FAM184B	ENSSSCG00000026232	100627969	8	12,634,377	12,741,488	protein_coding	family with sequence similarity 184 member B
DCAF16	ENSSSCG00000040393	100513483	8	12,751,313	12,751,963	protein_coding	DDB1 and CUL4 associated factor 16
NCAPG	ENSSSCG00000008747	100513670	8	12,764,210	12,810,050	protein_coding	non-SMC condensin I complex subunit G
LCORL	ENSSSCG00000008748	100337668	8	12,806,881	12,969,994	protein_coding	ligand dependent nuclear receptor corepressor like
SLIT2	ENSSSCG00000008749	100620577	8	14,989,795	15,147,974	protein_coding	slit guidance ligand 2
PACRGL	ENSSSCG00000008750	100515674	8	15,192,019	15,215,330	protein_coding	parkin coregulated like
KCNIP4	ENSSSCG00000023934	100516499	8	15,210,618	15,447,856	protein_coding	potassium voltage-gated channel interacting protein 4
ADGRA3	ENSSSCG00000008753	100516144	8	16,666,606	16,787,940	protein_coding	adhesion G protein-coupled receptor A3
GBA3	ENSSSCG00000008754	100737183	8	16,897,270	17,014,061	protein_coding	glucosylceramidase beta 3 (gene/pseudogene)
PPARGC1A	ENSSSCG00000029275	397013	8	17,841,844	17,961,834	protein_coding	PPARG coactivator 1 alpha
SOD3	ENSSSCG00000028814	780439	8	18,796,636	18,804,064	protein_coding	superoxide dismutase 3
CCDC149	ENSSSCG00000023001	100621948	8	18,807,732	18,914,011	protein_coding	coiled-coil domain containing 149
LGI2	ENSSSCG00000028927	100623207	8	18,987,047	19,017,500	protein_coding	leucine rich repeat LGI family member 2
SEPSECS	ENSSSCG00000025315	100622027	8	19,097,990	19,162,141	protein_coding	Sep (O-phosphoserine) tRNA:Sec (selenocysteine) tRNA synthase
ZCCHC4	ENSSSCG00000028581	100623403	8	19,252,097	19,295,252	protein_coding	zinc finger CCHC-type containing 4
ANAPC4	ENSSSCG00000025902	100518619	8	19,301,472	19,343,716	protein_coding	anaphase promoting complex subunit 4
ARAP2	ENSSSCG00000008768	100521564	8	27,771,337	28,100,520	protein_coding	ArfGAP with RhoGAP domain, ankyrin repeat and PH domain 2
DTHD1	ENSSSCG00000028471	100521908	8	27,998,300	28,072,201	protein_coding	death domain containing 1
NWD2	ENSSSCG00000008770	100522449	8	28,801,926	29,009,165	protein_coding	NACHT and WD repeat domain containing 2
C4orf19	ENSSSCG00000008771	100738623	8	29,013,082	29,103,934	protein_coding	chromosome 4 open reading frame 19
RELL1	ENSSSCG00000008772	100522085	8	29,118,512	29,190,567	protein_coding	RELT like 1
PGM2	ENSSSCG00000008773	100522261	8	29,307,613	29,381,571	protein_coding	phosphoglucomutase 2
TBC1D1	ENSSSCG00000028983	100524447	8	29,370,681	29,590,571	protein_coding	TBC1 domain family member 1

KLF3	ENSSSCG00000036488	100174960	8	30,024,830	30,060,982	protein_coding	Kruppel like factor 3
FAM114A1	ENSSSCG00000008785	100525173	8	30,114,602	30,274,166	protein_coding	family with sequence similarity 114 member A1
TLR1	ENSSSCG00000026583	396607	8	30,147,588	30,180,651	protein_coding	toll like receptor 1
TMEM156	ENSSSCG00000023785	100525349	8	30,287,162	30,342,780	protein_coding	transmembrane protein 156
KLHL5	ENSSSCG00000008787	100739193	8	30,361,386	30,440,991	protein_coding	kelch like family member 5
WDR19	ENSSSCG00000008789	100525651	8	30,463,781	30,553,703	protein_coding	WD repeat domain 19
RFC1	ENSSSCG00000008788	100511271	8	30,552,948	30,635,896	protein_coding	replication factor C subunit 1
KLB	ENSSSCG00000026297	100525825	8	30,653,556	30,691,344	protein_coding	klotho beta
LIAS	ENSSSCG00000029385	100627627	8	30,699,351	30,717,002	protein_coding	lipoic acid synthetase
UGDH	ENSSSCG00000030150	100627727	8	30,725,289	30,759,350	protein_coding	UDP-glucose 6-dehydrogenase
SMIM14	ENSSSCG00000021793	100627474	8	30,765,428	30,840,455	protein_coding	small integral membrane protein 14
UBE2K	ENSSSCG00000033602	780416	8	30,887,524	30,963,762	protein_coding	ubiquitin conjugating enzyme E2 K
PDS5A	ENSSSCG00000023112	NA	8	30,988,370	31,134,033	protein_coding	PDS5 cohesin associated factor A
N4BP2	ENSSSCG00000008792	100512116	8	31,231,943	31,284,621	protein_coding	NEDD4 binding protein 2
RHOH	ENSSSCG00000008794	100513215	8	31,323,264	31,372,065	protein_coding	ras homolog family member H
CHRNA9	ENSSSCG00000024428	100513414	8	31,441,444	31,456,384	protein_coding	cholinergic receptor nicotinic alpha 9 subunit
TEC	ENSSSCG00000008820	404277	8	38,051,061	38,181,874	protein_coding	tec protein tyrosine kinase
SLAIN2	ENSSSCG00000008821	100519161	8	38,241,501	38,321,142	protein_coding	SLAIN motif family member 2
SLC10A4	ENSSSCG00000008823	100520624	8	38,378,798	38,386,567	protein_coding	solute carrier family 10 member 4
ZAR1	ENSSSCG00000008824	574050	8	38,386,643	38,391,611	protein_coding	zygote arrest 1
FRYL	ENSSSCG00000008826	100520794	8	38,397,687	38,697,194	protein_coding	FRY like transcription coactivator
OCIAD1	ENSSSCG00000008828	100738601	8	38,745,425	38,769,267	protein_coding	OCIA domain containing 1
OCIAD2	ENSSSCG00000008829	100738638	8	38,769,952	38,793,370	protein_coding	OCIA domain containing 2
CWH43	ENSSSCG00000008830	100521324	8	38,869,125	38,943,483	protein_coding	cell wall biogenesis 43 C-terminal homolog
DCUN1D4	ENSSSCG00000008831	100521504	8	38,982,680	39,060,713	protein_coding	defective in cullin neddylation 1 domain containing 4
LRRC66	ENSSSCG00000008832	100522021	8	39,104,074	39,126,800	protein_coding	leucine rich repeat containing 66
SPATA18	ENSSSCG00000008834	100415808	8	39,148,874	39,203,956	protein_coding	spermatogenesis associated 18
RASL11B	ENSSSCG00000008835	100521670	8	39,703,362	39,709,817	protein_coding	RAS like family 11 member B
SCFD2	ENSSSCG00000008836	100738927	8	39,978,274	40,146,464	protein_coding	sec1 family domain containing 2
FIP1L1	ENSSSCG00000031636	100523128	8	40,157,270	40,235,093	protein_coding	factor interacting with PAPOLA and CPSF1
LNX1	ENSSSCG00000008838	100523319	8	40,232,845	40,473,087	protein_coding	ligand of numb-protein X 1
CHIC2	ENSSSCG00000032645	100524031	8	40,790,992	40,846,666	protein_coding	cysteine rich hydrophobic domain 2
GSX2	ENSSSCG00000008840	100524448	8	40,881,751	40,883,371	protein_coding	GS homeobox 2
KDR	ENSSSCG00000008844	397311	8	41,809,115	41,856,379	protein_coding	kinase insert domain receptor
PAPSS1	ENSSSCG00000031901	106504748	8	114,252,081	114,369,315	protein_coding	3'-phosphoadenosine 5'-phosphosulfate synthase 1
DKK2	ENSSSCG00000032094	100519672	8	114,862,075	114,974,316	protein_coding	dickkopf WNT signaling pathway inhibitor 2
GIMD1	ENSSSCG00000039563	100624542	8	115,413,515	115,429,130	protein_coding	GIMAP family P-loop NTPase domain containing 1
AIMP1	ENSSSCG00000024168	100135670	8	115,432,088	115,477,576	protein_coding	aminoacyl tRNA synthetase complex interacting multifunctional protein 1
TBCK	ENSSSCG00000021784	100738532	8	115,476,956	115,701,468	protein_coding	TBC1 domain containing kinase
NPNT	ENSSSCG00000038801	100624725	8	115,779,839	115,861,137	protein_coding	nephronectin
GSTCD	ENSSSCG00000023548	100624822	8	115,902,994	116,031,858	protein_coding	glutathione S-transferase C-terminal domain containing
INTS12	ENSSSCG00000030617	100625394	8	116,031,834	116,058,647	protein_coding	integrator complex subunit 12
ARHGEF38	ENSSSCG00000021764	100738766	8	116,063,745	116,199,330	protein_coding	Rho guanine nucleotide exchange factor 38
PPA2	ENSSSCG00000022788	100521105	8	116,158,074	116,334,013	protein_coding	pyrophosphatase (inorganic) 2
TET2	ENSSSCG00000009157	100521465	8	116,415,272	116,545,514	protein_coding	tet methylcytosine dioxygenase 2
CXXC4	ENSSSCG00000025621	100521810	8	117,097,853	117,121,034	protein_coding	CXXC finger protein 4
TACR3	ENSSSCG00000009160	100521983	8	117,661,792	117,721,591	protein_coding	tachykinin receptor 3
CENPE	ENSSSCG00000023296	100626792	8	117,888,970	117,965,792	protein_coding	centromere protein E
BDH2	ENSSSCG00000022282	100522692	8	117,969,992	117,995,216	protein_coding	3-hydroxybutyrate dehydrogenase 2
SLC9B2	ENSSSCG00000028101	100522336	8	117,996,843	118,079,194	protein_coding	solute carrier family 9 member B2
CISD2	ENSSSCG00000009164	100524158	8	118,152,701	118,164,654	protein_coding	CDGSH iron sulfur domain 2
GVQW3	ENSSSCG00000032157	102162715	9	10,566,782	10,572,435	protein_coding	GVQW motif containing 3
EMSY	ENSSSCG00000014868	100621182	9	10,628,053	10,716,398	protein_coding	EMSY, BRCA2 interacting transcriptional repressor
LRRC32	ENSSSCG00000014869	100522452	9	10,802,233	10,817,930	protein_coding	leucine rich repeat containing 32
TSKU	ENSSSCG00000014871	100522628	9	10,927,343	10,940,830	protein_coding	tsukushi, small leucine rich proteoglycan
ACER3	ENSSSCG00000032723	100524160	9	10,981,544	11,162,353	protein_coding	alkaline ceramidase 3
B3GNT6	ENSSSCG00000014874	100524328	9	11,176,045	11,178,111	protein_coding	UDP-GlcNAc:betaGal beta-1,3-N-acetylglucosaminyltransferase 6
CAPN5	ENSSSCG00000014875	100524513	9	11,194,297	11,246,712	protein_coding	calpain 5
PAK1	ENSSSCG00000014878	100525766	9	11,826,805	11,972,555	protein_coding	p21 (RAC1) activated kinase 1
CLNS1A	ENSSSCG00000014881	100623383	9	12,092,584	12,113,682	protein_coding	chloride nucleotide-sensitive channel 1A
INTS4	ENSSSCG00000014884	100512005	9	12,313,482	12,432,126	protein_coding	integrator complex subunit 4
ALG8	ENSSSCG00000014887	100511030	9	12,499,772	12,532,378	protein_coding	ALG8, alpha-1,3-glucosyltransferase
KCTD21	ENSSSCG00000033506	102158168	9	12,570,591	12,585,862	protein_coding	potassium channel tetramerization domain containing 21
USP35	ENSSSCG00000014892	100625395	9	12,592,675	12,662,023	protein_coding	ubiquitin specific peptidase 35
GAB2	ENSSSCG00000014891	100513609	9	12,663,661	12,851,374	protein_coding	GRB2 associated binding protein 2
NARS2	ENSSSCG00000014893	100514245	9	12,869,339	13,010,887	protein_coding	asparaginyl-tRNA synthetase 2, mitochondrial
TENM4	ENSSSCG00000014894	100514784	9	13,045,272	13,282,261	protein_coding	teneurin transmembrane protein 4
CDC73	ENSSSCG00000010801	100526068	10	575,075	667,480	protein_coding	cell division cycle 73
B3GALT2	ENSSSCG00000010800	100525885	10	613,586	621,439	protein_coding	beta-1,3-galactosyltransferase 2
GLRX2	ENSSSCG00000031991	100511215	10	679,309	687,349	protein_coding	glutaredoxin 2
TROVE2	ENSSSCG00000032055	NA	10	694,350	725,346	protein_coding	TROVE domain family member 2
RGS13	ENSSSCG00000040300	106505070	10	1,053,860	1,082,239	protein_coding	regulator of G protein signaling 13
RGS1	ENSSSCG00000032416	397336	10	1,144,392	1,152,122	protein_coding	regulator of G protein signaling 1
RGS21	ENSSSCG00000040323	100511580	10	1,338,922	1,394,229	protein_coding	regulator of G protein signaling 21
TEX14	ENSSSCG00000017645	100737915	12	34,815,476	34,912,269	protein_coding	testis expressed 14, intercellular bridge forming factor
RAD51C	ENSSSCG00000023955	100518577	12	34,975,979	35,019,019	protein_coding	RAD51 paralog C
PPM1E	ENSSSCG00000038932	100518751	12	35,051,954	35,262,698	protein_coding	protein phosphatase, Mg2+/Mn2+ dependent 1E
TRIM37	ENSSSCG00000023727	106505457	12	35,236,660	35,393,430	protein_coding	tripartite motif containing 37
SKA2	ENSSSCG00000034217	100520695	12	35,396,569	35,433,299	protein_coding	spindle and kinetochore associated complex subunit 2
MIR454	ENSSSCG00000018920	NA	12	35,419,607	35,419,718	miRNA	microRNA 454
PRR11	ENSSSCG00000024145	100624544	12	35,433,628	35,466,032	protein_coding	proline rich 11
YPEL2	ENSSSCG00000035400	100524105	12	35,573,363	35,644,194	protein_coding	yippee like 2
DHX40	ENSSSCG00000017665	100519272	12	35,781,578	35,828,501	protein_coding	DEAH-box helicase 40
CLTC	ENSSSCG00000022659	100271929	12	35,848,535	35,926,019	protein_coding	clathrin heavy chain
PTRH2	ENSSSCG00000017667	100519562	12	35,924,039	35,933,226	protein_coding	peptidyl-tRNA hydrolase 2
VMP1	ENSSSCG00000017668	NA	12	35,933,140	36,065,956	protein_coding	vacuole membrane protein 1
TUBD1	ENSSSCG00000017669	100520097	12	36,080,081	36,111,907	protein_coding	tubulin delta 1

RPS6KB1	ENSSSCG00000017670	100127158	12	36,111,579	36,160,383	protein_coding	ribosomal protein S6 kinase B1
MED13	ENSSSCG00000017672	100628203	12	36,333,191	36,429,291	protein_coding	mediator complex subunit 13
INTS2	ENSSSCG00000017673	100525182	12	36,435,812	36,494,591	protein_coding	integrator complex subunit 2
BRIP1	ENSSSCG00000021384	100525359	12	36,498,142	36,641,339	protein_coding	BRCA1 interacting protein C-terminal helicase 1
BCAS3	ENSSSCG00000017676	100620226	12	36,920,487	37,475,545	protein_coding	BCAS3, microtubule associated cell migration factor
PPM1D	ENSSSCG00000026665	100525714	12	37,485,588	37,536,164	protein_coding	protein phosphatase, Mg2+/Mn2+ dependent 1D
APPBP2	ENSSSCG00000017678	100525889	12	37,613,770	37,678,712	protein_coding	amyloid beta precursor protein binding protein 2
C17orf64	ENSSSCG00000017679	100511040	12	37,685,488	37,696,222	protein_coding	chromosome 17 open reading frame 64
ZNHIT3	ENSSSCG00000023373	100512308	12	38,013,944	38,027,921	protein_coding	zinc finger HIT-type containing 3
MYO19	ENSSSCG00000017682	100514070	12	38,017,343	38,062,020	protein_coding	myosin XIX
PIGW	ENSSSCG00000035006	110256038	12	38,061,955	38,065,708	protein_coding	phosphatidylinositol glycan anchor biosynthesis class W
GGNBP2	ENSSSCG00000017688	100512984	12	38,074,053	38,111,746	protein_coding	gametogenetin binding protein 2
MRM1	ENSSSCG00000017691	100513493	12	38,124,407	38,143,823	protein_coding	mitochondrial rRNA methyltransferase 1
LHX1	ENSSSCG00000017692	100513681	12	38,451,144	38,457,907	protein_coding	LIM homeobox 1
AATF	ENSSSCG00000017693	100513881	12	38,461,807	38,563,539	protein_coding	apoptosis antagonizing transcription factor
ACACA	ENSSSCG00000017694	397324	12	38,581,451	38,875,134	protein_coding	acetyl-CoA carboxylase alpha
C17orf78	ENSSSCG00000017696	100623319	12	38,841,882	38,853,575	protein_coding	chromosome 17 open reading frame 78
TADA2A	ENSSSCG00000025775	100737761	12	38,875,213	38,928,635	protein_coding	transcriptional adaptor 2A
DUSP14	ENSSSCG00000029874	100623524	12	38,941,124	38,974,912	protein_coding	dual specificity phosphatase 14
SYNRG	ENSSSCG00000026596	100624012	12	38,976,023	39,069,868	protein_coding	synergin gamma
DDX52	ENSSSCG00000021275	100623826	12	39,072,312	39,095,454	protein_coding	DExD-box helicase 52
HNF1B	ENSSSCG00000022417	397002	12	39,143,121	39,201,601	protein_coding	HNF1 homeobox B
HEATR6	ENSSSCG00000038478	100624283	12	39,307,465	39,347,406	protein_coding	HEAT repeat containing 6
CCL16	ENSSSCG00000040940	100516396	12	39,618,321	39,622,574	protein_coding	C-C motif chemokine ligand 16
HEATR9	ENSSSCG00000017706	100518148	12	39,663,199	39,679,669	protein_coding	HEAT repeat containing 9
MMP28	ENSSSCG00000034440	110256050	12	39,739,516	39,768,556	protein_coding	matrix metallopeptidase 28
C17orf50	ENSSSCG00000033580	NA	12	39,766,763	39,768,997	protein_coding	chromosome 17 open reading frame 50
GAS2L2	ENSSSCG00000039395	100516757	12	39,768,014	39,782,552	protein_coding	growth arrest specific 2 like 2
RASL10B	ENSSSCG00000036746	100516934	12	39,783,729	39,795,069	protein_coding	RAS like family 10 member B
AP2B1	ENSSSCG00000017712	100620457	12	39,800,965	39,940,780	protein_coding	adaptor related protein complex 2 subunit beta 1
PEX12	ENSSSCG00000017713	100517306	12	39,932,527	39,940,708	protein_coding	peroxisomal biogenesis factor 12
SLFN14	ENSSSCG00000031114	110256053	12	39,950,502	39,964,565	protein_coding	schlafen family member 14
NLE1	ENSSSCG00000025796	100626208	12	40,035,529	40,047,588	protein_coding	notchless homolog 1
FNDC8	ENSSSCG00000021617	100626291	12	40,046,095	40,052,597	protein_coding	fibronectin type III domain containing 8
RAD51D	ENSSSCG00000026989	100626849	12	40,054,181	40,068,821	protein_coding	RAD51 paralog D
RFFL	ENSSSCG00000032552	100627054	12	40,075,281	40,151,048	protein_coding	ring finger and FYVE like domain containing E3 ubiquitin protein ligase
LIG3	ENSSSCG00000024770	100626381	12	40,152,346	40,205,082	protein_coding	DNA ligase 3
CCT6B	ENSSSCG00000022196	100627253	12	40,205,096	40,230,006	protein_coding	chaperonin containing TCP1 subunit 6B
TMEM132E	ENSSSCG00000022987	100519732	12	40,481,784	40,497,809	protein_coding	transmembrane protein 132E
NTN1	ENSSSCG00000017993	733599	12	54,060,573	54,288,675	protein_coding	netrin 1
STX8	ENSSSCG00000034964	NA	12	54,296,537	54,544,918	protein_coding	syntaxin 8
CFAP52	ENSSSCG00000017994	100516337	12	54,544,590	54,581,846	protein_coding	cilia and flagella associated protein 52
USP43	ENSSSCG00000017995	100515447	12	54,584,353	54,642,376	protein_coding	ubiquitin specific peptidase 43
DHRS7C	ENSSSCG00000017996	100515625	12	54,639,773	54,661,662	protein_coding	dehydrogenase/reductase 7C
GLP2R	ENSSSCG00000017998	100515966	12	54,680,275	54,736,619	protein_coding	glucagon like peptide 2 receptor
RCVRN	ENSSSCG00000037991	100516153	12	54,740,644	54,750,355	protein_coding	recoverin
GAS7	ENSSSCG00000039056	100518690	12	54,752,497	54,973,527	protein_coding	growth arrest specific 7
MYH8	ENSSSCG00000018005	110255887	12	55,135,773	55,166,854	protein_coding	myosin heavy chain 8
MYH4	ENSSSCG00000018003	100144306	12	55,190,601	55,215,951	protein_coding	myosin heavy chain 4
MYH2	ENSSSCG00000029441	100125538	12	55,225,686	55,278,278	protein_coding	myosin heavy chain 2
SCO1	ENSSSCG00000018008	100517855	12	55,405,651	55,423,822	protein_coding	SCO1, cytochrome c oxidase assembly protein
ADPRM	ENSSSCG00000018009	100519388	12	55,430,702	55,436,067	protein_coding	ADP-ribose/CDP-alcohol diphosphatase, manganese dependent
TMEM220	ENSSSCG00000036454	100518038	12	55,438,849	55,454,585	protein_coding	transmembrane protein 220
PIRT	ENSSSCG00000037179	110255890	12	55,515,952	55,516,359	protein_coding	phosphoinositide interacting regulator of transient receptor potential channels
SHISA6	ENSSSCG00000024467	100624731	12	55,724,184	56,019,172	protein_coding	shisa family member 6
DNAH9	ENSSSCG00000018015	100518397	12	56,045,448	56,358,016	protein_coding	dynein axonemal heavy chain 9
ZNF18	ENSSSCG00000018014	100518217	12	56,359,738	56,385,098	protein_coding	zinc finger protein 18
MAP2K4	ENSSSCG00000018016	100520100	12	56,401,973	56,516,238	protein_coding	mitogen-activated protein kinase kinase 4
ARHGAP44	ENSSSCG00000018019	100520977	12	57,050,264	57,205,847	protein_coding	Rho GTPase activating protein 44
HS3ST3A1	ENSSSCG00000038622	100625836	12	57,631,469	57,729,589	protein_coding	heparan sulfate-glucosamine 3-sulfotransferase 3A1
ADAM12	ENSSSCG00000010746	100157228	14	135,302,758	135,682,281	protein_coding	ADAM metallopeptidase domain 12
C10orf90	ENSSSCG00000010747	100156286	14	135,719,517	135,802,615	protein_coding	chromosome 10 open reading frame 90
DOCK1	ENSSSCG00000035045	100524831	14	136,199,845	136,744,387	protein_coding	dedicator of cytokinesis 1
NPS	ENSSSCG00000010751	106506150	14	136,836,460	136,839,510	protein_coding	neuropeptide S
FOXI2	ENSSSCG00000010752	100157475	14	136,985,046	136,988,550	protein_coding	forkhead box I2
CLRN3	ENSSSCG00000010753	100156267	14	137,069,384	137,089,471	protein_coding	clarin 3
PTPRE	ENSSSCG00000010755	100155079	14	137,183,575	137,264,236	protein_coding	protein tyrosine phosphatase, receptor type E
MGMT	ENSSSCG00000010756	100155050	14	138,499,161	138,771,540	protein_coding	O-6-methylguanine-DNA methyltransferase
EBF3	ENSSSCG00000010757	100153848	14	138,819,298	139,004,257	protein_coding	EBF transcription factor 3
GLRX3	ENSSSCG00000010759	100511783	14	139,072,575	139,111,249	protein_coding	glutaredoxin 3
KLF7	ENSSSCG00000029998	100038003	15	110,286,490	110,379,145	protein_coding	Kruppel like factor 7
CREB1	ENSSSCG00000034395	100736562	15	110,705,580	110,772,401	protein_coding	cAMP responsive element binding protein 1
METTL21A	ENSSSCG00000036466	100518583	15	110,778,756	110,789,444	protein_coding	methyltransferase like 21A
CCNYL1	ENSSSCG00000024696	100520645	15	110,874,181	110,910,877	protein_coding	cyclin Y like 1
FZD5	ENSSSCG00000016140	100519058	15	110,916,008	110,921,353	protein_coding	frizzled class receptor 5
PLEKHM3	ENSSSCG00000016141	100520986	15	110,938,240	111,185,697	protein_coding	pleckstrin homology domain containing M3
CRYGC	ENSSSCG00000039620	110257071	15	111,256,725	111,258,161	protein_coding	crystallin gamma C
C2orf80	ENSSSCG00000032040	100623528	15	111,276,760	111,304,249	protein_coding	chromosome 2 open reading frame 80
PIKFYVE	ENSSSCG00000016147	100521692	15	111,371,992	111,467,899	protein_coding	phosphoinositide kinase, FYVE-type zinc finger containing
PTH2R	ENSSSCG00000016148	100170146	15	111,508,910	111,597,828	protein_coding	parathyroid hormone 2 receptor
MAP2	ENSSSCG00000016151	100153306	15	112,156,590	112,463,028	protein_coding	microtubule associated protein 2
UNC80	ENSSSCG00000016153	100522957	15	112,491,722	112,713,885	protein_coding	unc-80 homolog, NALCN channel complex subunit
KANSL1L	ENSSSCG00000022830	NA	15	112,763,186	112,896,614	protein_coding	KAT8 regulatory NSL complex subunit 1 like
LANCL1	ENSSSCG00000016158	100153701	15	113,111,368	113,149,061	protein_coding	LanC like 1
CPS1	ENSSSCG00000016159	100157716	15	113,209,631	113,334,672	protein_coding	carbamoyl-phosphate synthase 1
ERBB4	ENSSSCG00000016160	100525789	15	113,974,697	114,696,336	protein_coding	erb-b2 receptor tyrosine kinase 4

CFAP65	ENSSSCG00000016203	100155984	15	120,426,097	121,064,908	protein_coding	cilia and flagella associated protein 65
RNF25	ENSSSCG00000016202	100626865	15	120,709,741	120,720,171	protein_coding	ring finger protein 25
STK36	ENSSSCG00000026964	100519396	15	120,720,229	120,747,598	protein_coding	serine/threonine kinase 36
TTLL4	ENSSSCG00000016201	106506366	15	120,773,185	120,790,784	protein_coding	tubulin tyrosine ligase like 4
WNT6	ENSSSCG00000036932	100622044	15	120,901,283	120,917,398	protein_coding	Wnt family member 6
WNT10A	ENSSSCG00000026958	100623646	15	120,925,698	120,938,430	protein_coding	Wnt family member 10A
CDK5R2	ENSSSCG00000021584	100623529	15	120,985,257	120,986,354	protein_coding	cyclin dependent kinase 5 regulatory subunit 2
FEV	ENSSSCG00000035988	110257031	15	121,005,279	121,008,748	protein_coding	FEV, ETS transcription factor
CRYBA2	ENSSSCG00000040776	100519572	15	121,013,612	121,020,454	protein_coding	crystallin beta A2
IHH	ENSSSCG00000016204	397174	15	121,080,072	121,090,469	protein_coding	indian hedgehog
NHEJ1	ENSSSCG00000016205	110255182	15	121,100,628	121,194,048	protein_coding	non-homologous end joining factor 1
SLC23A3	ENSSSCG00000029694	100153546	15	121,195,249	121,203,986	protein_coding	solute carrier family 23 member 3
CNPPD1	ENSSSCG00000016206	100157604	15	121,204,268	121,212,893	protein_coding	cyclin Pas1/PHO80 domain containing 1
RETREG2	ENSSSCG00000016207	100156393	15	121,213,034	121,219,005	protein_coding	reticulophagy regulator family member 2
ABCB6	ENSSSCG00000016210	100155585	15	121,241,632	121,254,212	protein_coding	ATP binding cassette subfamily B member 6 (Langereis blood group)
ZFAND2B	ENSSSCG00000016208	100157982	15	121,243,114	121,247,531	protein_coding	zinc finger AN1-type containing 2B
GLB1L	ENSSSCG00000016213	100154356	15	121,273,915	121,283,303	protein_coding	galactosidase beta 1 like
TUBA4A	ENSSSCG00000016216	100151951	15	121,283,404	121,293,915	protein_coding	tubulin alpha 4a
STK16	ENSSSCG00000016214	100153147	15	121,284,920	121,292,244	protein_coding	serine/threonine kinase 16
DNAJB2	ENSSSCG00000016217	100522168	15	121,319,915	121,327,389	protein_coding	DnaJ heat shock protein family (Hsp40) member B2
PTPRN	ENSSSCG00000016218	100155609	15	121,329,882	121,349,136	protein_coding	protein tyrosine phosphatase, receptor type N
DNPEP	ENSSSCG00000016220	100153171	15	121,387,337	121,404,164	protein_coding	aspartyl aminopeptidase
DES	ENSSSCG00000020785	396725	15	121,427,532	121,444,938	protein_coding	desmin
ASIC4	ENSSSCG00000029968	100737498	15	121,522,569	121,547,917	protein_coding	acid sensing ion channel subunit family member 4
CHPF	ENSSSCG00000021610	100623047	15	121,547,669	121,553,595	protein_coding	chondroitin polymerizing factor
TMEM198	ENSSSCG00000027541	100623329	15	121,553,694	121,562,268	protein_coding	transmembrane protein 198
OBSL1	ENSSSCG00000028052	100624019	15	121,557,240	121,581,710	protein_coding	obscurin like 1
INHA	ENSSSCG00000020771	397386	15	121,582,044	121,586,845	protein_coding	inhibin subunit alpha
STK11IP	ENSSSCG00000032964	110256991	15	121,604,132	121,619,069	protein_coding	serine/threonine kinase 11 interacting protein
SLC4A3	ENSSSCG00000022846	100623083	15	121,630,316	121,643,296	protein_coding	solute carrier family 4 member 3
EPHA4	ENSSSCG00000016230	100188979	15	123,358,605	123,503,130	protein_coding	EPH receptor A4
PAX3	ENSSSCG00000028418	414901	15	124,095,296	124,192,625	protein_coding	paired box 3
SGPP2	ENSSSCG00000025993	100524832	15	124,318,271	124,463,060	protein_coding	sphingosine-1-phosphate phosphatase 2
MOGAT1	ENSSSCG00000016225	100524113	15	124,568,575	124,602,662	protein_coding	monoacylglycerol O-acyltransferase 1
DEPDC1B	ENSSSCG00000016932	100524052	16	39,466,500	39,557,985	protein_coding	DEP domain containing 1B
ELOVL7	ENSSSCG00000035012	100524592	16	39,588,124	39,680,488	protein_coding	ELOVL fatty acid elongase 7
ERCC8	ENSSSCG00000016934	100524405	16	39,704,605	39,770,315	protein_coding	ERCC excision repair 8, CSA ubiquitin ligase complex subunit
NDUFAF2	ENSSSCG00000027763	100738470	16	39,770,439	39,950,781	protein_coding	NADH:ubiquinone oxidoreductase complex assembly factor 2
ZSWIM6	ENSSSCG00000025194	100620165	16	40,105,426	40,305,375	protein_coding	zinc finger SWIM-type containing 6
KIF2A	ENSSSCG00000030186	100625826	16	40,971,713	41,102,545	protein_coding	kinesin family member 2A
DIMT1	ENSSSCG00000031990	100625553	16	41,097,818	41,111,257	protein_coding	DIM1 dimethyladenosine transferase 1 homolog
IPO11	ENSSSCG00000016938	100525544	16	41,121,007	41,522,669	protein_coding	importin 11
LRRC70	ENSSSCG00000037608	NA	16	41,292,987	41,295,640	protein_coding	leucine rich repeat containing 70
HTR1A	ENSSSCG00000016940	100526081	16	42,482,483	42,483,751	protein_coding	5-hydroxytryptamine receptor 1A
RNF180	ENSSSCG00000016941	100526254	16	42,686,965	42,952,053	protein_coding	ring finger protein 180
RGS7BP	ENSSSCG00000033581	100511050	16	43,088,792	43,192,884	protein_coding	regulator of G protein signaling 7 binding protein
SHISAL2B	ENSSSCG00000024514	100622319	16	43,273,284	43,297,243	protein_coding	shisa like 2B
CWC27	ENSSSCG00000016942	100511965	16	43,345,584	43,593,474	protein_coding	CWC27 spliceosome associated protein homolog
G3BP1	ENSSSCG00000035676	100513766	16	71,236,096	71,276,084	protein_coding	G3BP stress granule assembly factor 1
FAT2	ENSSSCG00000017084	102161049	16	71,469,847	71,530,501	protein_coding	FAT atypical cadherin 2
SLC36A1	ENSSSCG00000035442	100514926	16	71,504,776	71,589,545	protein_coding	solute carrier family 36 member 1
SLC36A2	ENSSSCG00000017086	100515104	16	71,662,109	71,689,217	protein_coding	solute carrier family 36 member 2
SLC36A3	ENSSSCG00000038877	106625371	16	71,686,370	71,734,972	protein_coding	solute carrier family 36 member 3
GM2A	ENSSSCG00000017087	100515283	16	71,740,319	71,754,975	protein_coding	GM2 ganglioside activator
CCDC69	ENSSSCG00000040411	100515590	16	71,778,420	71,820,841	protein_coding	coiled-coil domain containing 69
ANXA6	ENSSSCG00000017089	100515934	16	71,867,413	71,913,379	protein_coding	annexin A6
TNIP1	ENSSSCG00000017091	100271903	16	71,920,093	71,983,527	protein_coding	TNFAIP3 interacting protein 1
MARCH6	ENSSSCG00000017094	100170772	16	72,046,870	72,105,945	protein_coding	membrane associated ring-CH-type finger 6
CMBL	ENSSSCG00000039763	100625451	16	72,170,737	72,206,592	protein_coding	carboxymethylenebutenolidase homolog
CCT5	ENSSSCG00000024126	100627652	16	72,210,296	72,223,131	protein_coding	chaperonin containing TCP1 subunit 5
FAM173B	ENSSSCG00000036387	110257273	16	72,223,499	72,245,116	protein_coding	family with sequence similarity 173 member B
TAS2R1	ENSSSCG00000028554	106508396	16	72,720,854	72,721,753	protein_coding	taste 2 receptor member 1
SEMA5A	ENSSSCG00000017095	100737194	16	72,796,449	73,271,224	protein_coding	semaphorin 5A
MTRR	ENSSSCG00000017100	100516580	16	74,242,466	74,270,897	protein_coding	5-methyltetrahydrofolate-homocysteine methyltransferase reductase
FASTKD3	ENSSSCG00000017099	100516766	16	74,270,912	74,295,877	protein_coding	FAST kinase domains 3
C5orf49	ENSSSCG00000017098	106516406	16	74,288,049	74,302,047	protein_coding	chromosome 5 open reading frame 49
ADCY2	ENSSSCG00000017101	100517503	16	74,307,481	74,524,295	protein_coding	adenylate cyclase 2
TENT4A	ENSSSCG00000017102	100517864	16	75,217,219	75,260,149	protein_coding	terminal nucleotidyltransferase 4A
ADAMTS16	ENSSSCG00000017109	100519115	16	76,327,750	76,490,894	protein_coding	ADAM metallopeptidase with thrombospondin type 1 motif 16
IRX1	ENSSSCG00000034884	110257264	16	77,696,072	77,701,037	protein_coding	iroquois homeobox 1
IRX2	ENSSSCG00000032074	100620587	16	78,305,974	78,313,662	protein_coding	iroquois homeobox 2
IRX4	ENSSSCG00000017112	100519749	16	78,940,313	78,944,268	protein_coding	iroquois homeobox 4
NDUFS6	ENSSSCG00000017114	100520115	16	78,993,913	79,000,006	protein_coding	NADH:ubiquinone oxidoreductase subunit S6
MRPL36	ENSSSCG00000040154	100519932	16	79,001,508	79,002,777	protein_coding	mitochondrial ribosomal protein L36
LPCAT1	ENSSSCG00000017116	100217397	16	79,134,368	79,176,787	protein_coding	lysophosphatidylcholine acyltransferase 1
SLC6A3	ENSSSCG00000017121	100521346	16	79,186,053	79,220,411	protein_coding	solute carrier family 6 member 3
CLPTM1L	ENSSSCG00000017119	100520811	16	79,236,949	79,248,500	protein_coding	CLPTM1 like
SLC6A18	ENSSSCG00000017117	100520648	16	79,278,067	79,292,503	protein_coding	solute carrier family 6 member 18
SLC6A19	ENSSSCG00000017120	641346	16	79,292,962	79,310,904	protein_coding	solute carrier family 6 member 19
SLC12A7	ENSSSCG00000034302	110257347	16	79,335,324	79,399,155	protein_coding	solute carrier family 12 member 7
NKD2	ENSSSCG00000037791	110257348	16	79,399,139	79,420,313	protein_coding	naked cuticle homolog 2
TRIP13	ENSSSCG00000034522	100337674	16	79,458,603	79,473,727	protein_coding	thyroid hormone receptor interactor 13
BRD9	ENSSSCG00000035118	110257346	16	79,469,307	79,494,194	protein_coding	bromodomain containing 9
TPPP	ENSSSCG00000032698	100144410	16	79,535,009	79,556,382	protein_coding	tubulin polymerization promoting protein
CEP72	ENSSSCG00000031800	106508683	16	79,559,049	79,591,259	protein_coding	centrosomal protein 72
SLC9A3	ENSSSCG00000039413	397358	16	79,625,262	79,703,497	protein_coding	solute carrier family 9 member A3

EXOC3	ENSSSCG00000039607	100624948	16	79,705,744	79,730,277	protein_coding	exocyst complex component 3
AHRR	ENSSSCG00000032843	100624847	16	79,730,926	79,810,188	protein_coding	aryl-hydrocarbon receptor repressor
PDCD6	ENSSSCG00000032090	100620798	16	79,818,911	79,832,260	protein_coding	programmed cell death 6
SDHA	ENSSSCG00000020686	780433	16	79,838,154	79,906,500	protein_coding	succinate dehydrogenase complex flavoprotein subunit A
CCDC127	ENSSSCG00000036783	102163440	16	79,862,667	79,870,755	protein_coding	coiled-coil domain containing 127
LRRC14B	ENSSSCG00000033375	102161292	16	79,873,731	79,883,064	protein_coding	leucine rich repeat containing 14B
DNAJB9	ENSSSCG00000016653	100521593	18	36,578,516	36,584,732	protein_coding	DnaJ heat shock protein family (Hsp40) member B9
THAP5	ENSSSCG00000038158	100521761	18	36,584,386	36,592,905	protein_coding	THAP domain containing 5
GPR141	ENSSSCG00000016655	100521938	18	36,611,203	36,662,070	protein_coding	G protein-coupled receptor 141
ELMO1	ENSSSCG00000016656	100522109	18	36,786,422	37,338,970	protein_coding	engulfment and cell motility 1
AOAH	ENSSSCG00000016657	100522290	18	37,377,924	37,599,931	protein_coding	acyloxyacyl hydrolase
ANLN	ENSSSCG00000016658	100523340	18	37,645,004	37,708,565	protein_coding	anillin actin binding protein
KIAA0895	ENSSSCG00000016659	100522963	18	37,724,469	37,793,150	protein_coding	KIAA0895
EEPD1	ENSSSCG00000039703	100738248	18	37,824,452	37,948,860	protein_coding	endonuclease/exonuclease/phosphatase family domain containing 1
SEPT7	ENSSSCG00000016661	100523875	18	38,082,569	38,183,916	protein_coding	septin 7
HERPUD2	ENSSSCG00000027389	100738688	18	38,284,520	38,326,392	protein_coding	HERPUD family member 2
TBX20	ENSSSCG00000016662	100524471	18	38,635,526	38,677,132	protein_coding	T-box 20
DPY19L2	ENSSSCG00000016663	100524658	18	38,693,003	38,764,024	protein_coding	dpy-19 like 2
DPY19L1	ENSSSCG00000027063	100525550	18	38,800,720	38,906,316	protein_coding	dpy-19 like C-mannosyltransferase 1
NPSR1	ENSSSCG00000016664	100217399	18	38,942,887	39,212,434	protein_coding	neuropeptide S receptor 1
BMPER	ENSSSCG00000016665	100525197	18	39,417,837	39,663,999	protein_coding	BMP binding endothelial regulator

Supplementary Table S3. Sheet 2: Results of analysis in gene ontology and pathway participation with EnrichR, David, Panther and GOTermMapper tools.

TOOL	DATABASE	TERM/PATHWAY	GENES
**ENRICHER** http://amp.pharm.mssm.edu/Enrichr/	KEGG_2016	Fatty acid elongation_Homo sapiens_hsa00062	*PPT1;ELOVL7;PPT2;HSD17B12*
		Fatty acid metabolism_Homo sapiens_hsa01212	*PPT1;PPT2;HSD17B12;ACACA*
		Non-alcoholic fatty liver disease (NAFLD)_Homo sapiens_hsa04932	*COX8C;NDUFS7;NDUFS6;COX4I1;SDHA*
		Sphingolipid signaling pathway_Homo sapiens_hsa04071	*PPP2R2B;GAB2;S1PR4;SGPP2*
		Glycerophospholipid metabolism_Homo sapiens_hsa00564	*LPCAT1;ADPRM;PLB1*
		Regulation of lipolysis in adipocytes_Homo sapiens_hsa04923	*ADCY2;ADRB2;TSHR*
		Adipocytokine signaling pathway_Homo sapiens_hsa04920	*RXRB;STK11;PPARGC1A*
		Ether lipid metabolism_Homo sapiens_hsa00565	*LPCAT1;PLB1*
		Sphingolipid metabolism_Homo sapiens_hsa00600	*ACER3;SGPP2*
		Glycosphingolipid biosynthesis - lacto and neolacto series_Homo sapiens_hsa00601	*B3GALT2*
		Biosynthesis of unsaturated fatty acids_Homo sapiens_hsa01040	*HSD17B12*
		Fatty acid biosynthesis_Homo sapiens_hsa00061	*ACACA*
	REACTOME_2016	ABC transporters in lipid homeostasis_Homo sapiens_R-HSA-1369062	*ABCD3;ABCA7*
		Acyl chain remodelling of PC_Homo sapiens_R-HSA-1482788	*LPCAT1;PLB1*
		Fatty acid, triacylglycerol, and ketone body metabolism_Homo sapiens_R-HSA-535734	*MOGAT1;ACOT12;LPCAT1;RORA;ELOVL7;HSD17B12;ACACA;MED28;MED13;BDH2;MTF1;PPT1;PLIN3;PPT2;PPARGC1A;HMGCLL1;PPARGC1B*
		Fatty Acids bound to GPR40 (FFAR1) regulate insulin secretion_Homo sapiens_R-HSA-434316	*GNA15*
		Fatty Acyl-CoA Biosynthesis_Homo sapiens_R-HSA-75105	*ACOT12;PPT1;ELOVL7;PPT2;HSD17B12;ACACA*
		Free fatty acids regulate insulin secretion_Homo sapiens_R-HSA-400451	*GNA15*
		Glycerophospholipid biosynthesis_Homo sapiens_R-HSA-1483206	*SLC44A3;SLC44A4;LPCAT1;PLB1*
		Glycosphingolipid metabolism_Homo sapiens_R-HSA-1660662	*GLB1L;GM2A;ARSK;ARSI;GBA3*
		Hormone-sensitive lipase (HSL)-mediated triacylglycerol hydrolysis_Homo sapiens_R-HSA-163560	*PPP1CB*
		Lipid digestion, mobilization, and transport_Homo sapiens_R-HSA-73923	*PPP1CB*
		Lysosphingolipid and LPA receptors_Homo sapiens_R-HSA-419408	*S1PR4*
		Metabolism of fat-soluble vitamins_Homo sapiens_R-HSA-6806667	*APOM;PLB1;LGMN*
		Metabolism of lipids and lipoproteins_Homo sapiens_R-HSA-556833	*SLC44A3;SLC44A4;MOGAT1;LPCAT1;RORA;HSD17B12;GBA3;ACACA;PLB1;PPP1CB;COL4A3BP;MED13;GM2A;ARSK;CYP1B1;ARSI;PIP5K1C;PPARGC1A;HMGCLL1;PPARGC1B;MBTPS1;STARD4;SRD5A2;ACOT12;ELOVL7;SGPP2;MED28;FA2H;NUDT7;PIKFYVE;BDH2;GLB1L;CYP21A2;MTF1;ACER3;PPT1;PLIN3;PPT2;CSNK1G2*
		Peroxisomal lipid metabolism_Homo sapiens_R-HSA-390918	*NUDT7*
		Phospholipid metabolism_Homo sapiens_R-HSA-1483257	*PIKFYVE;SLC44A3;SLC44A4;LPCAT1;PIP5K1C;PLB1*
		Regulation of lipid metabolism by Peroxisome proliferator-activated receptor alpha (PPARalpha)_Homo sapiens_R-HSA-400206	*MED13;MTF1;RORA;PPARGC1A;PPARGC1B;MED28*
		Sphingolipid de novo biosynthesis_Homo sapiens_R-HSA-1660661	*FA2H;COL4A3BP;ACER3;SGPP2;CSNK1G2*
		Sphingolipid metabolism_Homo sapiens_R-HSA-428157	*FA2H;COL4A3BP;GLB1L;GM2A;ACER3;ARSK;ARSI;GBA3;SGPP2;CSNK1G2*
		Synthesis of very long-chain fatty acyl-CoAs_Homo sapiens_R-HSA-75876	*PPT1;ELOVL7;HSD17B12;PPT2*

	WikiPathways	Adipogenesis_Homo sapiens_WP236	*FOXC2;KLF7;MEF2C;CREB1;SOCS1;GADD45B;NR2F1;RORA;PPARGC1A;ZMPSTE24*
		Differentiation of white and brown adipocyte _Homo sapiens_WP2895	*EBF3;PPARGC1A;PPARGC1B*
		Nuclear Receptors in Lipid Metabolism and Toxicity_Homo	*ABCD3*
		Fatty Acid Biosynthesis_Homo sapiens_WP357	*ACACA*
		Transcription factor regulation in adipogenesis_Homo sapiens_WP3599	*CREB1;PPARGC1A*
		Triacylglyceride Synthesis_Homo sapiens_WP325	*MOGAT1*
		Globo Sphingolipid Metabolism_Homo sapiens_WP1424	*ST3GAL2*
		Sphingolipid Metabolism_Homo sapiens_WP1422	*SGPP2*
		SREBF and miR33 in cholesterol and lipid homeostasis_Homo sapiens_WP2011	*PPARGC1A*
		Ganglio Sphingolipid Metabolism_Homo sapiens_WP1423	*ST3GAL2*
		Leptin and adiponectin_Mus musculus_WP683	*ACACA*
**GENERIC GENE ONTOLOGY (GO) TERM MAPPER http://go.princeton.edu/cgi-bin/GOTermMapper**	GO: BIOLOGICAL PROCESS	lipid metabolic process (GO:0006629)	*ABCA4, ABCD3, ABHD1, ABHD16A, ACACA, ACER3, ACOT12, ALG14, ALG8, AOAH, APOM, B3GALT2, BDH2, CD74, CHGA, COL4A3BP, CPS1, CREB1, CSF1R, CSNK1G2, CWH43, CYP1B1, CYP21A2, ELOVL7, ERBB4, FA2H, GBA3, GFI1, GM 2A, HMGCLL1, HSD17B12, HSD17B8, INPP5B, KLB, LACTB, LGMN, LIAS, LPCAT1, MBTPS1, MFSD2A, MOGAT1, NDUFS 6, NUDT7, PDGFRB, PDPR, PIAS4, PIGW, PIKFYVE, PIP5K1C, PLB1, PLIN5, PPARGC1A, PPT1, PSMB8, RORA, SEL1L, SERINC5, SERPINA12, SERPINA6, SGPP2, SLC44A3, SLC44A4, SNX17, SOCS1, SRD5A2, ST3GAL2, STARD4, STK11, TTC7B*
**DAVID 6.8** https://david.ncifcrf.gov/summary.jsp	GOTERM_BP_DIRECT	GO:0035338~long-chain fatty-acyl-CoA biosynthetic process	*HSD17B12, ACACA, ACOT12, PPT2, ELOVL7, PPT1*
	GOTERM_BP_DIRECT	GO:0030148~sphingolipid biosynthetic process	*ACER3, CSNK1G2, COL4A3BP, FA2H, SGPP2, ELOVL7*
	UP_KEYWORDS	Lipid droplet	*PLIN5, LPCAT1, PLIN4, PLIN3, SPAST*
**PANTHER** http://pantherdb.org/	GO-Slim Biological Process	fatty acid metabolic process: (GO:0006631)	*LIAS, ANXA2, ELOVL7, CAD, ACOT12, ABHD1, FA2H, ANXA6*
		coenzyme metabolic process (GO:0006732)	*COL4A3BP, ABCA4, ABCD3, ABCA7, UCN*
		acyl-CoA metabolic process (GO:0006637)	*ACOT12, MOGAT1*
		lipid metabolic process (GO:0006629)	*LIAS, ANXA2, ELOVL7, CAD, ACOT12, ABHD1, FA2H, ANXA6, COL4A3BP, ABCA4, ABCD3, ABCA7, UCN, ACOT12, MOGAT1, CHGA*
		fatty acid biosynthetic process (GO:0006633)	*LIAS, ELOVL7, ACOT12,ABHD1*
	GO-Slim Molecular Function	lipid transporter activity (GO:0005319)	*ATP8B3, SLC10A4*
		phospholipase activity (GO:0004620)	*PLB1, GNA15, F2RL1, F2RL2*
		lipase activity (go;0016298)	*PLB1, GNA15, F2RL1, F2RL2, ABHD1*
		lipid binding (GO:0008289)	*SNX1, SNX17, TLR1, COQ9*

Supplementary Table S3. Sheet 3: List of the 85 genes functionally catalogued as related to the metabolism, transport or storage of fatty acids

hgnc_symbol	ensembl_gene_id	chromosome name	start position	end position	gene_biotype	Approved Name
SNX1	ENSSSCG00000004556	1	107,909,671	107,952,265	protein_coding	sorting nexin 1
LACTB	ENSSSCG00000004569	1	108,909,702	108,924,511	protein_coding	lactamase beta
RORA	ENSSSCG00000004576	1	111,375,423	111,477,664	protein_coding	RAR related orphan receptor A
ANXA2	ENSSSCG00000004578	1	111,588,306	111,635,100	protein_coding	annexin A2
HSD17B12	ENSSSCG00000021739	2	18,458,890	18,611,765	protein_coding	hydroxysteroid 17-beta dehydrogenase 12
PLIN3	ENSSSCG00000038954	2	73,969,909	73,997,400	protein_coding	perilipin 3
PLIN5	ENSSSCG00000013513	2	74,304,634	74,314,315	protein_coding	perilipin 5
PLIN4	ENSSSCG00000013512	2	74,318,628	74,331,930	protein_coding	perilipin 4
PIAS4	ENSSSCG00000022066	2	74,699,939	74,729,789	protein_coding	protein inhibitor of activated STAT 4
PIP5K1C	ENSSSCG00000013490	2	74,977,266	75,046,895	protein_coding	phosphatidylinositol-4-phosphate 5-kinase type 1 gamma
S1PR4	ENSSSCG00000013474	2	75,396,170	75,397,483	protein_coding	sphingosine-1-phosphate receptor 4
GNA15	ENSSSCG00000013473	2	75,408,726	75,429,903	protein_coding	G protein subunit alpha 15
GADD45B	ENSSSCG00000022689	2	76,106,916	76,111,479	protein_coding	growth arrest and DNA damage inducible beta
CSNK1G2	ENSSSCG00000013445	2	76,573,985	76,609,575	protein_coding	casein kinase 1 gamma 2
ATP8B3	ENSSSCG00000013440	2	76,722,254	76,746,255	protein_coding	ATPase phospholipid transporting 8B3
NDUFS7	ENSSSCG00000024144	2	77,076,573	77,087,126	protein_coding	NADH:ubiquinone oxidoreductase core subunit S7
STK11	ENSSSCG00000013429	2	77,231,796	77,252,075	protein_coding	serine/threonine kinase 11
ABCA7	ENSSSCG00000023121	2	77,357,212	77,375,170	protein_coding	ATP binding cassette subfamily A member 7
COL4A3BP	ENSSSCG00000014081	2	84,417,409	84,537,369	protein_coding	collagen type IV alpha 3 binding protein
F2RL2	ENSSSCG00000037582	2	85,535,662	85,542,145	protein_coding	coagulation factor II thrombin receptor like 2
F2RL1	ENSSSCG00000014091	2	85,732,082	85,747,621	protein_coding	F2R like trypsin receptor 1
SERINC5	ENSSSCG00000014119	2	88,843,020	88,961,132	protein_coding	serine incorporator 5
ACOT12	ENSSSCG00000036095	2	89,855,166	89,905,197	protein_coding	acyl-CoA thioesterase 12
MEF2C	ENSSSCG00000014149	2	96,122,044	96,296,902	protein_coding	myocyte enhancer factor 2C
NR2F1	ENSSSCG00000014157	2	100,447,904	100,457,904	protein_coding	nuclear receptor subfamily 2 group F member 1
ARSK	ENSSSCG00000014139	2	102,164,091	102,209,755	protein_coding	arylsulfatase family member K
STARD4	ENSSSCG00000031518	2	116,234,587	116,257,041	protein_coding	StAR related lipid transfer domain containing 4
PPP2R2B	ENSSSCG00000028976	2	147,937,442	148,411,731	protein_coding	protein phosphatase 2 regulatory subunit Bbeta
ADRB2	ENSSSCG00000038598	2	150,033,550	150,035,599	protein_coding	adrenoceptor beta 2
PPARGC1B	ENSSSCG00000014437	2	150,823,368	150,919,366	protein_coding	PPARG coactivator 1 beta
CSF1R	ENSSSCG00000014441	2	151,099,084	151,129,407	protein_coding	colony stimulating factor 1 receptor
PDGFRB	ENSSSCG00000022741	2	151,155,754	151,192,816	protein_coding	platelet derived growth factor receptor beta
ARSI	ENSSSCG00000014448	2	151,315,508	151,320,814	protein_coding	arylsulfatase family member I
CD74	ENSSSCG00000033262	2	151,399,969	151,433,844	protein_coding	CD74 molecule
SOCS1	ENSSSCG00000027855	3	31,881,891	31,882,722	protein_coding	suppressor of cytokine signaling 1
CYP1B1	ENSSSCG00000033844	3	102,195,741	102,206,375	protein_coding	cytochrome P450 family 1 subfamily B member 1
SPAST	ENSSSCG00000008517	3	107,442,593	107,508,619	protein_coding	spastin
SRD5A2	ENSSSCG00000008521	3	107,840,200	107,918,350	protein_coding	steroid 5 alpha-reductase 2
PPP1CB	ENSSSCG00000008540	3	110,436,526	110,474,765	protein_coding	protein phosphatase 1 catalytic subunit beta
PLB1	ENSSSCG00000034756	3	110,605,923	110,741,130	protein_coding	phospholipase B1
SNX17	ENSSSCG00000025363	3	111,775,426	111,781,361	protein_coding	sorting nexin 17
UCN	ENSSSCG00000039206	3	111,834,181	111,835,997	protein_coding	urocortin
CAD	ENSSSCG00000028978	3	111,887,294	111,914,302	protein_coding	carbamoyl-phosphate synthetase 2, aspartate transcarbamylase, and dihydroorotase
ABHD1	ENSSSCG00000008554	3	111,991,257	112,002,148	protein_coding	abhydrolase domain containing 1
ALG14	ENSSSCG00000006885	4	122,343,312	122,460,755	protein_coding	ALG14, UDP-N-acetylglucosaminyltransferase subunit
SLC44A3	ENSSSCG00000006888	4	122,529,983	122,621,379	protein_coding	solute carrier family 44 member 3
ABCD3	ENSSSCG00000028620	4	122,837,525	122,953,257	protein_coding	ATP binding cassette subfamily D member 3
ABCA4	ENSSSCG00000006890	4	123,202,753	123,331,144	protein_coding	ATP binding cassette subfamily A member 4
GFI1	ENSSSCG00000006902	4	124,622,419	124,629,180	protein_coding	growth factor independent 1 transcriptional repressor
FOXC2	ENSSSCG00000029296	6	2,552,862	2,554,367	protein_coding	forkhead box C2
COX4I1	ENSSSCG00000029034	6	3,131,019	3,137,482	protein_coding	cytochrome c oxidase subunit 4I1
MBTPS1	ENSSSCG00000002682	6	4,503,479	4,558,615	protein_coding	membrane bound transcription factor peptidase, site 1
NUDT7	ENSSSCG00000002697	6	10,161,960	10,173,980	protein_coding	nudix hydrolase 7
FA2H	ENSSSCG00000002718	6	12,855,421	12,909,499	protein_coding	fatty acid 2-hydroxylase
PDPR	ENSSSCG00000002724	6	13,187,422	13,230,598	protein_coding	pyruvate dehydrogenase phosphatase regulatory subunit
ST3GAL2	ENSSSCG00000038843	6	13,402,345	13,456,455	protein_coding	ST3 beta-galactoside alpha-2,3-sialyltransferase 2
COQ9	ENSSSCG00000025284	6	19,373,047	19,390,735	protein_coding	coenzyme Q9
MTF1	ENSSSCG00000037516	6	93,839,200	93,884,417	protein_coding	metal regulatory transcription factor 1
INPP5B	ENSSSCG00000003642	6	93,885,459	93,944,384	protein_coding	inositol polyphosphate-5-phosphatase B
MFSD2A	ENSSSCG00000003669	6	95,738,805	95,756,002	protein_coding	major facilitator superfamily domain containing 2A
PPT1	ENSSSCG00000036673	6	95,839,159	95,861,692	protein_coding	palmitoyl-protein thioesterase 1
ZMPSTE24	ENSSSCG00000027902	6	96,008,304	96,057,123	protein_coding	zinc metallopeptidase STE24
APOM	ENSSSCG00000001411	7	23,770,896	23,774,428	protein_coding	apolipoprotein M
ABHD16A	ENSSSCG00000034923	7	23,799,337	23,824,299	protein_coding	abhydrolase domain containing 16A
SLC44A4	ENSSSCG00000001419	7	23,958,941	23,978,188	protein_coding	solute carrier family 44 member 4
CYP21A2	ENSSSCG00000001428	7	24,086,258	24,089,371	protein_coding	cytochrome P450 family 21 subfamily A member 2
PPT2	ENSSSCG00000001433	7	24,188,608	24,202,622	protein_coding	palmitoyl-protein thioesterase 2
PSMB8	ENSSSCG00000026951	7	25,053,132	25,073,046	protein_coding	proteasome subunit beta 8
RXRB	ENSSSCG00000001474	7	25,236,009	25,243,176	protein_coding	retinoid X receptor beta
HSD17B8	ENSSSCG00000001476	7	25,247,608	25,249,786	protein_coding	hydroxysteroid 17-beta dehydrogenase 8
HMGCLL1	ENSSSCG00000001479	7	25,596,716	25,783,612	protein_coding	3-hydroxymethyl-3-methylglutaryl-CoA lyase like 1
TSHR	ENSSSCG00000031771	7	103,768,260	103,926,287	protein_coding	thyroid stimulating hormone receptor
SEL1L	ENSSSCG00000002414	7	104,218,296	104,283,256	protein_coding	SEL1L, ERAD E3 ligase adaptor subunit
TTC7B	ENSSSCG00000002436	7	112,268,301	112,519,208	protein_coding	tetratricopeptide repeat domain 7B

LGMN	ENSSSCG00000002452	7	114,175,183	114,217,319	protein_coding	legumain
CHGA	ENSSSCG00000002456	7	114,345,091	114,358,571	protein_coding	chromogranin 1
COX8C	ENSSSCG00000002462	7	114,727,655	114,729,235	protein_coding	cytochrome c oxidase subunit 8C
SERPINA6	ENSSSCG00000002475	7	115,535,471	115,554,817	protein_coding	serpin family A member 6
SERPINA12	ENSSSCG00000002478	7	115,720,677	115,734,816	protein_coding	serpin family A member 12
MED28	ENSSSCG00000029989	8	12,624,104	12,633,248	protein_coding	mediator complex subunit 28
GBA3	ENSSSCG00000008754	8	16,897,270	17,014,061	protein_coding	glucosylceramidase beta 3 (gene/pseudogene)
PPARGC1A	ENSSSCG00000029275	8	17,841,844	17,961,834	protein_coding	PPARG coactivator 1 alpha
TLR1	ENSSSCG00000026583	8	30,147,588	30,180,651	protein_coding	toll like receptor 1
KLB	ENSSSCG00000026297	8	30,653,556	30,691,344	protein_coding	klotho beta
LIAS	ENSSSCG00000029385	8	30,699,351	30,717,002	protein_coding	lipoic acid synthetase
SLC10A4	ENSSSCG00000008823	8	38,378,798	38,386,567	protein_coding	solute carrier family 10 member 4
CWH43	ENSSSCG00000008830	8	38,869,125	38,943,483	protein_coding	cell wall biogenesis 43 C-terminal homolog
BDH2	ENSSSCG00000022282	8	117,969,992	117,995,216	protein_coding	3-hydroxybutyrate dehydrogenase 2
ACER3	ENSSSCG00000032723	9	10,981,544	11,162,353	protein_coding	alkaline ceramidase 3
ALG8	ENSSSCG00000014887	9	12,499,772	12,532,378	protein_coding	ALG8, alpha-1,3-glucosyltransferase
GAB2	ENSSSCG00000014891	9	12,663,661	12,851,374	protein_coding	GRB2 associated binding protein 2
B3GALT2	ENSSSCG00000010800	10	613,586	621,439	protein_coding	beta-1,3-galactosyltransferase 2
MED13	ENSSSCG00000017672	12	36,333,191	36,429,291	protein_coding	mediator complex subunit 13
PIGW	ENSSSCG00000035006	12	38,061,955	38,065,708	protein_coding	phosphatidylinositol glycan anchor biosynthesis class W
ACACA	ENSSSCG00000017694	12	38,581,451	38,875,134	protein_coding	acetyl-CoA carboxylase alpha
ADPRM	ENSSSCG00000018009	12	55,430,702	55,436,067	protein_coding	ADP-ribose/CDP-alcohol diphosphatase, manganese dependent
EBF3	ENSSSCG00000010757	14	138,819,298	139,004,257	protein_coding	EBF transcription factor 3
KLF7	ENSSSCG00000029998	15	110,286,490	110,379,145	protein_coding	Kruppel like factor 7
CREB1	ENSSSCG00000034395	15	110,705,580	110,772,401	protein_coding	cAMP responsive element binding protein 1
PIKFYVE	ENSSSCG00000016147	15	111,371,992	111,467,899	protein_coding	phosphoinositide kinase, FYVE-type zinc finger containing
CPS1	ENSSSCG00000016159	15	113,209,631	113,334,672	protein_coding	carbamoyl-phosphate synthase 1
ERBB4	ENSSSCG00000016160	15	113,974,697	114,696,336	protein_coding	erb-b2 receptor tyrosine kinase 4
GLB1L	ENSSSCG00000016213	15	121,273,915	121,283,303	protein_coding	galactosidase beta 1 like
SGPP2	ENSSSCG00000025993	15	124,318,271	124,463,060	protein_coding	sphingosine-1-phosphate phosphatase 2
MOGAT1	ENSSSCG00000016225	15	124,568,575	124,602,662	protein_coding	monoacylglycerol O-acyltransferase 1
ELOVL7	ENSSSCG00000035012	16	39,588,124	39,680,488	protein_coding	ELOVL fatty acid elongase 7
GM2A	ENSSSCG00000017087	16	71,740,319	71,754,975	protein_coding	GM2 ganglioside activator
ADCY2	ENSSSCG00000017101	16	74,307,481	74,524,295	protein_coding	adenylate cyclase 2
NDUFS6	ENSSSCG00000017114	16	78,993,913	79,000,006	protein_coding	NADH:ubiquinone oxidoreductase subunit S6
LPCAT1	ENSSSCG00000017116	16	79,134,368	79,176,787	protein_coding	lysophosphatidylcholine acyltransferase 1
SDHA	ENSSSCG00000020686	16	79,838,154	79,906,500	protein_coding	succinate dehydrogenase complex flavoprotein subunit A
AOAH	ENSSSCG00000016657	18	37,377,924	37,599,931	protein_coding	acyloxyacyl hydrolase

Supplementary Table S4. Effect of the polymorphisms in 5 candidate genes on *longissimus thoracis* intramuscular fatty acid composition in a resource population of three pure Iberian varieties.

Gene	SNP	Trait[1]	Genotypes compared	Difference in trait content	SE	95% CI	p-value
RXRB	**rs327226101**	C16:0	TT-GG	0.80	0.27	0.15, 1.44	**0.011**
			TT-GT	0.56	0.20	0.08, 1.04	**0.018**
		C18:0	TT-GG	0.67	0.28	0.01, 1.32	**0.045**
		C18:1	TT-GG	-0.85	0.40	-1.78, 0.09	0.086
			TT-GT	-0.84	0.29	-1.54, -0.14	**0.013**
		SFA	TT-GG	1.47	0.49	0.32, 2.63	**0.008**
			TT-GT	0.97	0.36	0.11, 1.83	**0.023**
		MUFA	TT-GG	-1.12	0.47	-2.22, -0.02	**0.046**
			TT-GT	-0.94	0.35	-1.77, -0.12	**0.020**
		PUFA	TT-GG	-0.37	0.15	-0.72, -0.03	**0.028**
			GT-GG	-0.35	0.13	-0.67, -0.03	**0.026**
		MUFA/SFA	TT-GG	-0.09	0.03	-0.16, -0.02	**0.013**
			TT-GT	-0.06	0.02	-0.12, -0.01	**0.014**
	**rs80789331**	C14:0	CC-CA	0.13	0.04	0.03, 0.23	**0.008**
			CC-AA	0.10	0.05	-0.01, 0.21	0.075
		C16:0	CC-AA	0.94	0.26	0.31, 1.56	**0.001**
			CC-CA	0.86	0.24	0.30, 1.42	**0.001**
		C18:1	CC-CA	-1.25	0.34	-2.06, -0.43	**0.001**
			CC-AA	-1.08	0.38	-1.98, -0.17	**0.016**
		SFA	CC-AA	1.47	0.48	0.34, 2.60	**0.007**
			CC-CA	1.24	0.43	0.23, 2.26	**0.012**
		MUFA	CC-CA	-1.33	0.41	-2.30, -0.37	**0.004**
			CC-AA	-1.24	0.45	-2.31, -0.17	**0.019**
		PUFA	CA-AA	-0.33	0.11	-0.60, -0.06	**0.011**
		MUFA/SFA	CC-AA	-0.09	0.03	-0.16, -0.02	**0.009**
			CC-CA	-0.08	0.03	-0.14, -0.02	**0.005**
PSMB8	**rs80801731**	C16:0	GG-AG	-0.58	0.23	-1.12, -0.03	**0.036**
		C16:1	AG-AA	0.32	0.15	-0.02, 0.66	0.074
		C18:1	GG-AG	0.85	0.33	0.06, 1.63	**0.031**
		MUFA	GG-AG	0.82	0.39	-0.11, 1.74	0.096
CHGA	**rs322866746**	C18:0	GG-AG	1.21	0.44	0.18, 2.24	**0.017**

		C18:1	GG-AG	-1.54	0.64	-3.04, -0.04	**0.043**
		SFA	GG-AG	2.09	0.78	0.24, 3.94	**0.023**
		MUFA	GG-AG	-1.92	0.75	-3.68, -0.15	**0.030**
		MUFA/SFA	GG-AG	-0.13	0.05	-0.24, -0.02	**0.021**
ACACA	**rs340781986**	C18:1	CC-TT	0.82	0.38	-0.07, 1.71	0.080
	**12:38727103G>A**	C16:0	GG-AA	-1.16	0.35	-1.99, -0.34	**0.003**
			GG-AG	-1.09	0.33	-1.86, -0.31	**0.003**
		C18:0	GG-AG	-0.84	0.33	-1.61. -0.06	**0.030**
		C18:1	GG-AG	1.67	0.47	0.56, 2.79	**0.001**
			GG-AA	1.66	0.50	0.48, 2.84	**0.003**
		SFA	GG-AG	-1.98	0.58	-3.36, -0.60	**0.003**
			GG-AA	-1.86	0.62	-3.32, -0.40	**0.008**
		MUFA	GG-AG	1.90	0.55	0.59, 3.21	**0.002**
			GG-AA	1.77	0.59	0.38, 3.16	**0.008**
		MUFA/SFA	GG-AG	0.13	0.04	0.04, 0.21	**0.001**
			GG-AA	0.12	0.04	0.03, 0.21	**0.003**

[1]All traits as percentage of total fatty acid content. SFA - saturated fatty acids; MUFA - monounsaturated fatty acids; PUFA - polyunsaturated fatty acids; SE - Standard error

Supplementary Table S5. Effect of the polymorphisms in 5 candidate genes on *longissimus thoracis* intramuscular fatty acid composition in three distinct Iberian varieties.

Gene	Iberian variety	SNP	Trait[1]	Genotypes compared	Difference in trait content	SE	95% CI	p-value
RXRB	Retinto	rs327226101	C16:0	TT-GG	1.99	0.53	0.71, 3.28	**0.001**
				TT-GT	1.51	0.43	0.48, 2.54	**0.002**
			C16:1	TT-GG	-0.80	0.31	-1.53, -0.06	**0.032**
			C18:0	TT-GG	1.93	0.55	0.62, 3.25	**0.002**
				TT-GT	0.95	0.44	-0.11, 2.00	0.086
			C18:1	TT-GG	-2.28	0.78	-4.14, -0.41	**0.013**
				TT-GT	-1.98	0.62	-3.47, -0.48	**0.007**
			SFA	TT-GG	3.90	1.02	1.44, 6.36	**0.001**
				TT-GT	2.52	0.82	0.55, 4.49	**0.009**
			MUFA	TT-GG	-3.18	0.98	-5.53, -0.83	**0.005**
				TT-GT	-2.40	0.78	-4.28, -0.53	**0.009**
			PUFA	TT-GG	-0.75	0.21	-1.25, -0.25	**0.002**
				TT-GT	-0.62	0.19	-1.09, -0.16	**0.006**
			MUFA/SFA	TT-GG	-0.23	0.07	-0.39, -0.07	**0.002**
				TT-GT	-0.16	0.05	-0.29, -0.04	**0.008**
		rs80789331	C16:0	CC-AA	1.89	0.53	0.62, 3.16	**0.002**
				CC-CA	1.56	0.43	0.52, 2.60	**0.002**
			C16:1	CC-AA	-0.82	0.30	-1.54, -0.09	**0.023**
			C18:0	CC-AA	2.02	0.53	0.74, 3.30	**0.001**
				CA-AA	1.06	0.49	-0.11, 2.24	0.084
				CC-CA	0.96	0.44	-0.09, 2.00	0.081
			C18:1	CC-AA	-2.30	0.76	-4.12, -0.47	**0.010**
				CC-CA	-2.08	0.62	-3.58, -0.59	**0.004**
			SFA	CC-AA	3.88	1.01	1.47, 6.30	**0.001**
				CC-CA	2.59	0.82	0.60, 4.57	**0.007**
			MUFA	CC-AA	-3.25	0.95	-5.54, -0.95	**0.003**
				CC-CA	-2.52	0.78	-4.40, -0.64	**0.006**
			PUFA	CC-AA	-0.66	0.21	-1.16, -0.15	**0.007**
				CC-CA	-0.58	0.19	-1.04, -0.12	**0.010**
			MUFA/SFA	CC-AA	-0.23	0.06	-0.38, -0.08	**0.002**
				CC-CA	-0.17	0.05	-0.29, -0.04	**0.006**

CHGA	Retinto	rs318914549	C16:0	CT-TT	-1.54	0.59	-2.97, -0.12	**0.030**
			C18:1	CT-TT	2.60	0.81	0.66, 4.54	**0.006**
				CC-TT	2.45	0.91	0.27, 4.64	**0.024**
			SFA	CT-TT	-2.78	1.13	-5.50, -0.05	**0.045**
			MUFA	CT-TT	3.02	1.04	0.51, 5.52	**0.015**
				CC-TT	2.74	1.17	-0.08, 5.56	0.058
			MUFA/SFA	CT-TT	0.17	0.07	0.00, 0.35	**0.047**
	Entrepelado	rs322866746	C16:0	AG-AA	-0.37	0.11	-0.60, -0.14	**0.003**
			C18:0	AG-AA	-0.67	0.20	-1.09, -0.25	**0.004**
			C18:1	AG-AA	0.68	0.22	0.22, 1.14	**0.007**
			SFA	AG-AA	-1.06	0.30	-1.69, -0.43	**0.002**
			MUFA	AG-AA	0.80	0.29	0.19, 1.41	**0.014**
			PUFA	AG-AA	0.26	0.14	-0.03, 0.55	0.071
			MUFA/SFA	AG-AA	0.06	0.02	0.02, 0.09	**0.003**
ACACA	Retinto	12:38727103G>A	C16:0	AG-GG	1.24	0.52	-0.01, 2.49	0.052
			C18:1	GG-AG	1.99	0.71	0.27, 3.71	**0.020**
				GG-AA	1.81	0.86	-0.25, 3.87	0.096
			SFA	GG-AG	-2.28	0.99	-4.67, 0.10	0.063
			MUFA	GG-AG	2.30	0.92	0.09, 4.50	**0.040**
			MUFA/SFA	GG-AG	0.15	0.06	0.00, 0.30	**0.043**

[1]All traits as percentage of total fatty acid content. SFA - saturated fatty acids; MUFA - monounsaturated fatty acids; PUFA - polyunsaturated fatty acids; SE - Standard error

Supplementary Table S6. (A) Primers used for the amplification of coding and regulatory regions of candidate genes selected from the most prominent GWAS regions. Main PCR conditions were: 1×Buffer, 2 mM $MgCl_2$, 0.16 mM dNTPs, 0.5 uM primers and 0.5 U Taq Polymerase (Bioline) in 25 ul reaction volume. Deviation from these concentrations are indicated in the table. **(B)** Primers used to genotype selected SNP mutations by high resolution melting (HRM) analysis in a QS3 thermocycler (LifeTechnologies) under supplier's recommended conditions. Luminaris HRM Master Mix (ThermoFisher) was used in the HRM reactions with 0.4 uM of each primer pair and 10-15 ng of genomic DNA.

Table S6.A.

Gene	Region[1] SSC(Mb)	Position	Primer name	Primers sequence (5'→ 3')	From	Fragment size	Annealing T°	PCR conditions
PLIN5	**2(74.3)**	promoter	PLIN5_prom_F	GCTCAATCCTTCGGTATCCA	genomic	1300 bp	58ºC	
			PLIN5_prom_R	TTCTTCTGACATGCCTGCTG				
		cds	PLIN5_exon1_F	GGAGCAGGACCAGCAGAA	cDNA	915 bp	58ºC	1U of Taq polymerase
			PLIN5_exon7_R	CTGGTCTCCAGCAAGTCCAC				
			PLIN5_exon7_F	GTGGACTTGCTGGAGACCA	cDNA	958 bp	60ºC	1U of Taq polymerase
			PLIN5_exon8_R	CTGGAGATCAAATCCCAAGC				
PSMB8	**7(25.0)**	promoter	PSMB8_prom_F	GGTGTGGCTAGAACCCTGAA	genomic	1350 bp	58ºC	
			PSMB8_prom_R	CTCGCAGGAGGAAGTGAAAG				
		cds	PSMB8_cDNA_F	CGCGGGGTGGAAGATTGAG	cDNA	1060 bp	58ºC	1U of Taq polymerase
			PSMB8_cDNA_R	GCTCCAGTGCTTGCCGC				
RXRB	**7(25.2)**	promoter	RXRB_prom_F	ATGATCGTGACCGTGGGTAT	genomic	1007 bp	58ºC	
			RXRB_prom_R	GAGTGTAGGGATGCCGAGAG				
		cds	RXRB_cDNA_F1	GGGGGTGCGAAAAGAAATGC	cDNA	1192 bp	60ºC	
			RXRB_cDNA_R1	CACCCGGTCAAAGATGGCT				
			RXRB_cDNA_F2	AGCCATCTTTGACCGGGTG	cDNA	1286 bp	60ºC	
			RXRB_cDNA_R2	CAGAGCTGTGACGGTTAGGG				
CHGA	**7(114.3)**	promoter	promCHGA_F	TGTGCCTCCCTAAAACAGCC	genomic	1187 bp	60ºC	2.5 mM $MgCl_2$
			promCHGA_R	GCGCCCCACTTATATAGCCC				

		cds	CHGA_exon1-2_F	AGGGATGGGAAAGGGGTAG		1229 bp	58ºC	1U of Taq polymerase
			CHGA_exon1-2_R	ATCCCACACCCCGTCTTAC				
			CHGA_exon3-4_F	CAACCTGCCCCACTCTCTT		1087 bp	58ºC	1U of Taq polymerase
			CHGA_exon3-4_R	CTGGAATTTCCCCAGATTCA				
			CHGA_exon6_F	GGCCTGAGTTTACTCCGTTG		550 bp	54ºC	
			CHGA_exon6_R	AGGGTTGTTGTGAAGGTGGA				
			CHGA_exon6	AGAAGAGGCGTCCTCCAAG		1600 bp	58ºC	1U of Taq polymerase
			CHGA_exon8	CCTTCTTCTCCTCCAGGTAGC				
			CHGA_exon9_F	ATGACCCTTGGCATTCTGTC		280 bp	58ºC	
			CHGA_exon9_R	AGCAGGAGCAGGAGGAAAG				
ACACA	**12(38.5)**	promoter	ACACA_prom_F	GAGCTTAGAGGATGGCTTGG	genomic	1117 bp	58ºC	1U of Taq polymerase
			ACACA_prom_R	TCCCATTGTTGCAGAAGACA				
		enhancer	ACACA_enh_F1	CAGCTGACTGCCAGGAGAC		924 bp	56ºC	
			ACACA_enh_R1	TTCTGGGGCTTTTGAAGTTG				
			ACACA_enh_F2	CAACTTCAAAAGCCCCAGAA		980 bp	56ºC	
			ACACA_enh_R2	GGAGCTCAGGCCTTCAATTT				

[1]Sscrofa 11.1 assembly

Table S6.B

Gene	Region	SNP	Position[1]	SNP_id[2]	Prediction	Primer name	Primers sequence (5'→ 3')	PCR size
PLIN5	exon 3	C>A	2:74306608		p.Ala75His (non-conservative)	PLIN5_exon2_F	TATAGCGCCGCCAAGGAC	120 bp
						PLIN5_exon2_R	CAGGTGGCTGAGCAGTGG	
RXRB	promoter	T>G	7: 25243210	rs327226101	potential Sp1 binding site	RXRBprom_K2_F	TCTTTATCCCGAACCCCTTC	139 bp
						RXRBprom_K2_R	TCTCTGCTGTCCTTTCCAAG	
	promoter/5'UTR	G>A	7:25243071	rs331605500	potential C/EBPa binding site	RXRBprom_R2_F	AAAAGCGGGCTATTCTCACC	102 bp
						RXRBprom_R2_R	AACCGAATTCCCTGTTCTCG	
	exon 9	C>A	7:25237419	rs80789331	Synonymous/Splice region variant	RXRB_exon9_AS_F	GGAGACCTATTGCAAACAGAAG	74 bp
						RXRB_exon9_AS_R	AACCCCTCAGACCCACAG	
PSMB8	promoter	G>A	7:25062476	rs80801731	potential AP2alpha binding site	PSMB8prom_R1_F	ATACAGCGTGGTCGTCTGAG	62 bp
						PSMB8prom_R1_R	AGGTGGTGAAGCGTGAGC	
CHGA	exon 6	G>A	7:114353907	rs322866746	p.Thr167Ala	CHGA ex6-T167A Fw	AACGATGAAGACGCAGACG	98 bp
						CHGA ex6-T167A Rv	CAGCCTCCTCCTCCCCCG	
	exon 6	C>T	7:114354144	rs318914549	p.Pro246Ser	CHGA ex6-P246S Fw	CGCAGCGAAGCATTTGAC	76 bp
						CHGA ex6-P246S Rv	AGGCATCACACATACTCTCACC	
ACACA	putative enhancer (intron 24)	G>A	12:38727103		putative enhancer; intron 24.3387	ACACA_i25.3387_Fw	TGGGCATATGGCATTACTCC	95 bp
						ACACA_i25.3387_Rv	GCCTTCCCCTCACTCACTTAC	
	putative enhancer (intron 24)	T>G	12:38726462		putative enhancer; intron 24.2746	ACACA_i25.2746_Fw	ACCTCTCCCCATCCTGCTC	59 bp
						ACACA_i25.2746_Rv	AACTTTCTTTCCAGGGCTGAG	
	exon 49	C>T	12:340781986	rs340781986	Synonymous variant	ACACA-5634_F	GCATCCAGATCATGCACAAC	105 bp
						ACACA-5634_R	TCACCTTGGGCATGTAAGAC	

[1]Sscrofa 11.1 assembly
[2]dbSNP; https://www.ncbi.nlm.nih.gov/snp/

Supplementary Figures

Supplementary Figure S1. Manhanttan plot showing genome-wide Bayes factor (BF) values of association with *longissimus thoracis* intramuscular fat (IMF) content and composition. Dotted variety indicates threshold of BF>10. **(A)** IMF; **(B)** palmitoleic fatty acid content; **(C)** oleic fatty acid content; **(D)** saturated fatty acid (SFA) content; **(E)** monounsaturated fatty acid (MUFA) content; **(F)** MUFA to SFA ratio.

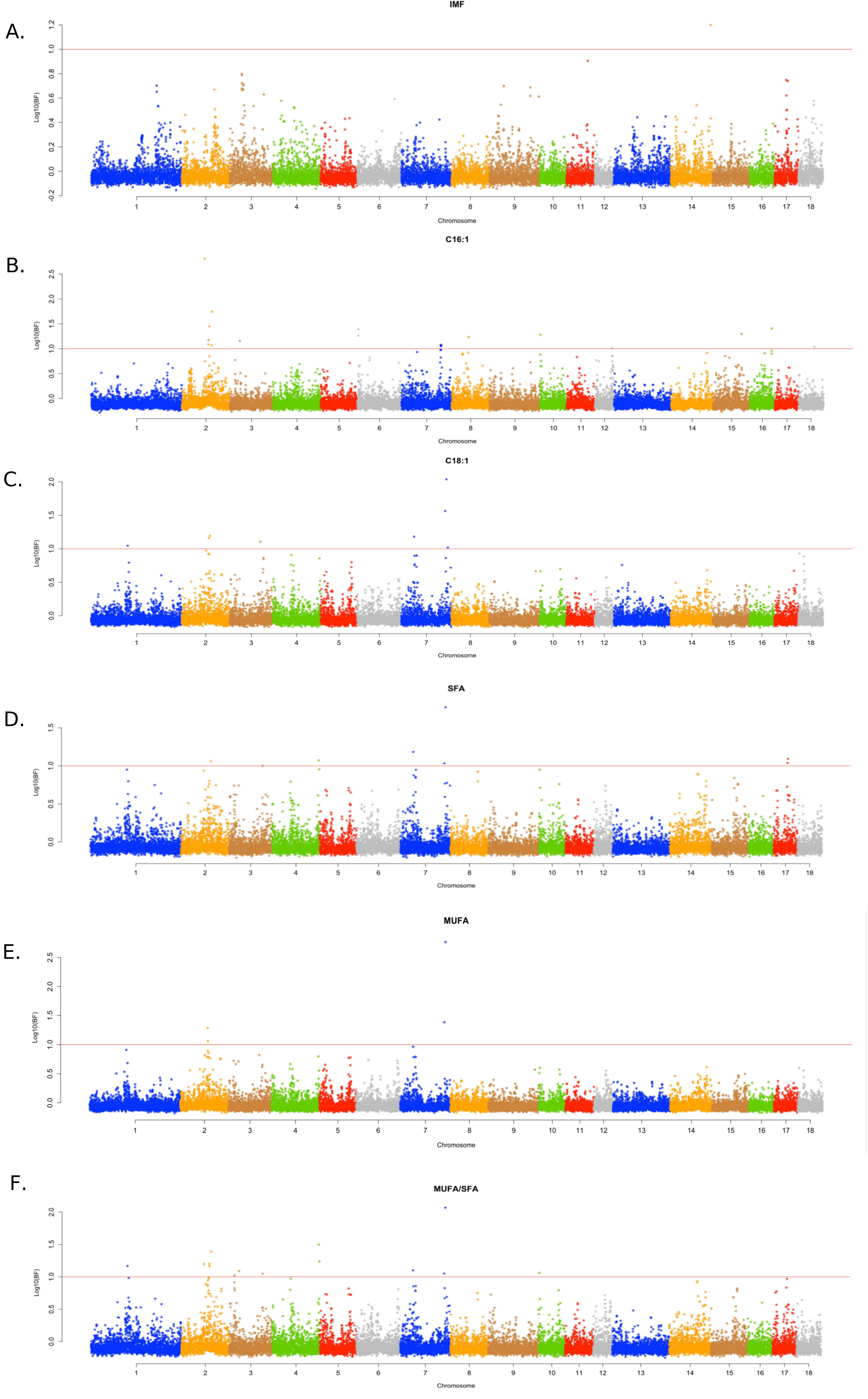
A.
IMF
B.
C16:1
C.
C18:1
D.
SFA
E.
MUFA
F.
MUFA/SFA
Log10(BF)
Chromosome
1 2 3 4 5 6 7 8 9 10 11 12 13 14 15 16 17 18
